# Microbes Saving Lives and Reducing Suffering

**DOI:** 10.1111/1751-7915.70068

**Published:** 2025-01-22

**Authors:** Kenneth Timmis, Zeynep Ceren Karahan, Juan Luis Ramos, Omry Koren, Ana Elena Pérez‐Cobas, Karen Steward, Victor de Lorenzo, Elisabetta Caselli, Margaret Douglas, Clarissa Schwab, Virginia Rivero, Rafael Giraldo, Junkal Garmendia, Raymond J. Turner, Jessamyn Perlmutter, José M. Borrero de Acuña, Pablo Ivan Nikel, Jerome Bonnet, Angela Sessitsch, James K. Timmis, Carla Pruzzo, M. Auxiliadora Prieto, Siavash Isazadeh, Wei E. Huang, Gerard Clarke, Danilo Ercolini, Max Häggblom

**Affiliations:** ^1^ Institute of Microbiology Technical University Braunschweig Braunschweig Germany; ^2^ Department of Medical Microbiology and Ibn‐i Sina Hospital Central Microbiology Laboratory Ankara University School of Medicine Ankara Turkey; ^3^ Consejo Superior de Investigaciones Científicas, Estación Experimental del Zaidín Granada Spain; ^4^ Azrieli Faculty of Medicine Bar‐Ilan University Safed Israel; ^5^ Department of Microbiology, Ramón y Cajal Institute for Health Research (IRYCIS) Ramón y Cajal University Hospital Madrid Spain; ^6^ CIBER in Infectious Diseases (CIBERINFEC) Madrid Spain; ^7^ Technology Networks Sudbury Suffolk UK; ^8^ Department of Systems Biology National Centre of Biotechnology CSIC Madrid Spain; ^9^ Section of Microbiology, Department of Environmental and Prevention Sciences University of Ferrara Ferrara Italy; ^10^ Usher Institute University of Edinburgh Medical School, and Public Health Scotland Edinburgh UK; ^11^ Department of Biological and Chemical Engineering Aarhus University Aarhus Denmark; ^12^ Polymer Biotechnology Lab, Biological Research Center Margarita Salas Spanish National Research Council (CIB‐CSIC) Madrid Spain; ^13^ Department of Microbial Biotechnology National Centre for Biotechnology (CNB‐CSIC) Madrid Spain; ^14^ Instituto de Agrobiotecnología Consejo Superior de Investigaciones Científicas (IdAB‐CSIC)‐Gobierno de Navarra, Mutilva Madrid Spain; ^15^ Centro de Investigación Biomédica en Red de Enfermedades Respiratorias (CIBERES) Madrid Spain; ^16^ Department of Biological Sciences University of Calgary Calgary Alberta Canada; ^17^ Department of Molecular Biosciences University of Kansas Lawrence Kansas USA; ^18^ Department of Microbiology Universidad de Sevilla Sevilla Spain; ^19^ The Novo Nordisk Foundation Center for Biosustainability Technical University of Denmark Lyngby Denmark; ^20^ Centre de Biochimie Structurale, INSERM/CNRS University of Montpellier Montpellier France; ^21^ Bioresources Unit AIT Austrian Institute of Technology Vienna Austria; ^22^ Department of Political Science University of Freiburg Freiburg Germany; ^23^ Athena Institute for Research on Innovation and Communication in Health and Life Sciences Vrije Universiteit Amsterdam The Netherlands; ^24^ Department of Earth, Environmental and Life Sciences (DISTAV) University of Genoa Genova Italy; ^25^ Corporate Technical & Performance Veolia North America Paramus New Jersey USA; ^26^ Department of Engineering Science University of Oxford Oxford UK; ^27^ APC Microbiome Ireland University College Cork Cork Ireland; ^28^ Department of Psychiatry & Neurobehavioral Sciences University College Cork Cork Ireland; ^29^ Department of Agricultural Sciences University of Naples Federico II Naples Italy; ^30^ Department of Biochemistry and Microbiology, Rutgers The State University of New Jersey New Brunswick New Jersey USA

## 1 Introduction

Given the overexploitation of the resources of planet Earth, due in large part to the ever‐increasing human population (https://www.un.org/sustainabledevelopment/sustainable‐consumption‐production/), which has already compromised vital planetary processes (https://reports.weforum.org/docs/WEF_Business_on_the_Edge_2024.pdf), the limitations of which are encapsulated in planetary boundaries (Richardson et al. 2023; Gupta et al. 2024; https://www.pik‐potsdam.de/en/news/latest‐news/earth‐exceed‐safe‐limits‐first‐planetary‐health‐check‐issues‐red‐alert) and climate tipping points (Wunderling et al. 2023; Wunderling, von der Heydt, and Aksenov 2024), it would not be unexpected that a visitor from Mars might well be confused, or at least bemused, by our efforts to save lives and reduce morbidity. The Martian might be similarly bemused when it learned that although warfare is a constant feature of biosphere ecology, including human behaviour, with military personnel of opposing armies doing their best to kill one another, military physicians will try their best to save the lives of injured prisoners of the opposing side. But warfare and other activities of individuals and groups aimed at harming others notwithstanding, saving lives and preventing/reducing human suffering is an ingrained moral‐ethical‐humanitarian imperative (https://www.ohchr.org/sites/default/files/Documents/Publications/Factsheet31.pdf). While we cannot prevent death, we try hard to prevent avoidable, premature death and disease. But trying hard is not the same as succeeding (Kruk et al. 2018). This is reflected in the United Nations Sustainable Development Goal (SDG) 3 *Ensure healthy lives and promote well‐being for all at all ages* which identifies major deficits in global healthcare and provides a roadmap to correct these deficits (https://sdgs.un.org/2030agenda).

The pursuit of saving lives and ameliorating human suffering is arguably the highest calling of humankind. Though generally considered to be the domain of clinicians—the healers—it clearly includes the endeavours of other health professionals, emergency responders, carers, parents–family–friends, the pharmaceutical industry, international organisations and a variety of non‐governmental organisations. More indirectly it includes *inter alia* those of engineers, educators, the body politic and financial services. Microbial technologies, exemplified by vaccines and microbially inspired and produced pharmaceuticals and diagnostics, play a central role in the prevention, amelioration and curing of disease, saving millions of lives and reducing billions of cases of suffering every year (https://immunizationdata.who.int). Moreover, life‐saving microbial technologies play out not only in the healthcare sector but also in wastewater and drinking water monitoring and treatment (Fowler and Smets 2017), food provision, bioremediation, etc. As a consequence, they rank very high among human endeavours to prevent and counter disease. Microbial technologies are thus central to the aims of SDG 3. Moreover, given that new life‐threatening problems, such as diverse impacts of global warming (Lenton et al. 2023), have arisen and appropriate microbial technologies either exist or can be developed to contribute to their mitigation, the scope and scale of life‐saving/−prolonging/−improving microbial solutions will continue to grow (Verstraete et al. 2022).

Despite this, microbes, if at all discussed in strategy documents, are usually mentioned only in the context of problems they pose (causing disease, food deterioration, materials corrosion, etc.), rarely as solutions they can provide for problems, and consequently are massively underexploited. Reasons for this include *germophobia* (the prevalent view of microbes as being dangerous *germs*, to be feared and therefore killed), their invisibility (*out of sight, out of mind*, which means they are not on the radar screens of most decision‐makers) and the fact that their vital importance to the well‐being of humanity, food plants and animals, climate, the biosphere is a relatively recent realisation that has not yet permeated the body of general knowledge. While this is slowly changing, time is not on our side in confronting pressing issues and crises which demand immediate implementation of effective solutions. We need to accelerate appreciation of the power of microbes to address problems and the deployment of relevant microbial technologies (Timmis, de Lorenzo, et al. 2017; Timmis, de Vos, et al. 2017).

In exhorting leaders and policy decision‐makers to exploit microbial technologies to mitigate and solve major problems and global crises, we ask them to step outside of their comfort zones, enter the (for them) new information world of the microbiologist, see the bigger picture and engage in systems thinking. However, in order to be effective in this endeavour, we, the scientists and microbiologists, must also appreciate the wider context, be systems thinkers and communicate the bigger picture. But most of us only feel authoritative in discussing our own narrow field of activity, because our scientific training inhibits us from expressing opinions about issues the rigour of which we are not able to verify. This key element of scientific training, which guides our personal academic activities, can in fact promote ‘silo’ rather than systems thinking, and a reluctance to step outside of the comfort zones of our own specialities. The constant and justified exhortation to engage in inter‐ and trans‐disciplinary research in which some of the most significant discoveries are to be made is only modestly successful, partly because of this and partly because of the difficulty of finding willing and capable assessors of grant applications for such projects and subsequent manuscript submissions, which as a consequence often result in unwarranted rejection, disappointment and discouragement to engage further in such research. Therefore, if microbiologists want society to take full advantage of the power and potential of currently unfamiliar microbial technologies, and for leaders and policy‐makers to step outside their comfort zones and take the risk (for them) of implementing new solutions they only incompletely understand, we must ourselves step outside of our comfort zones and take the risk of engaging in conversations of broader issues.

This Editorial seeks:

- To highlight the diversity, range, interconnectedness and interdependencies of the wide range of very different causes of human suffering and mortality, on the necessity of consideration of individual problems within broader contexts, of viewing them in the context of systems healthcare and of the necessity of systems thinking for solving systems problems, and on the need to address causes and remedies at their roots, not only in the hospital (see also https://www.goinvo.com/features/determinants‐of‐health/#:~:text=Health%20is%20More%20Than%20Medical,the%20social%20determinants%20of%20health),
- To outline and map on the spectrum of health challenges the exceptional range of available and emerging microbial technology solutions that raise barriers to preventable human suffering, morbidity and premature mortality, that is, that *directly address the aims of SDG 3*, and thereby raise awareness of some vital problem‐solving options that may be inadequately appreciated by decision‐makers,
- To present human suffering in the wider context, including policy, lifestyles and behaviour, environment‐climate change‐planetary boundaries, conflicts and technological potentials, in order to identify bottlenecks to progress and focus on plausible countermeasures, and
- To provide a framework for the *Microbial Biotechnology* Special Issue *The Contribution of Microbial Biotechnology to Sustainable Development Goal 3: Ensure healthy lives and promote well‐being for all at all ages*, of which this Editorial is a part.

## 2 Principal Causes of Human Disease and Key Risk Factors

The burden of disease is usually expressed in terms of disability‐adjusted life years (DALYs): one DALY represents the loss of the equivalent of 1 year of full health. ‘DALYs for a disease or health condition are the sum of years of life lost (YLLs) due to premature mortality and years of healthy life lost due to disability (YLDs) due to prevalent cases of the disease or health condition in a population’. (https://www.who.int/data/gho/indicator‐metadata‐registry/imr‐details/158#:~:text=DALYs%20for%20a%20disease%20or,%2C%20Sex%2C%20Cause%2C%20Risk%20factors). According to the World Health Organisation (WHO) report for 2020–21 (https://www.who.int/news‐room/fact‐sheets/detail/the‐top‐10‐causes‐of‐death), cardiovascular, respiratory and infectious diseases were leading causes of death globally, with significant differences between low/medium income countries (LMICs) and high‐income countries (HICs) (see also https://ourworldindata.org/burden‐of‐disease; https://www.healthdata.org/research‐analysis/library/global‐burden‐disease‐2021‐findings‐gbd‐2021‐study). Cancer and dementia are also important in HICs.

Microbes are centrally involved in initiation and progression of disease in some of these classes. However, and crucially, microbes and their activities can be harnessed to reduce disease so, for our discourse, the lens of predisposing parameters/risk factors of disease is of greatest interest because this reveals intervention options for detection‐monitoring, prevention and treatment (Ezzati et al. 2002). Often, risk factors fall into the classes of too much (e.g., exposure to air pollution, untreated drinking water sources and unhealthy food) or too little (e.g., food, micronutrients and exercise). According to Ezzati et al. (2002), ‘In the poorest regions of the world, childhood and maternal underweight, unsafe sex, unsafe water, sanitation, and hygiene, indoor smoke from solid fuels, and various micronutrient deficiencies were major contributors to loss of healthy life. In both developing and developed regions, alcohol, tobacco, high blood pressure, and high cholesterol were major causes of disease burden’. It is also important to keep in mind that preventable human suffering also has many other causes, including poverty, abuse, warfare, migrations, trafficking, accidents, lack of education and, especially, global warming, some of which can be addressed with microbial technologies. In this discourse, we review these diverse health risk factors in the context of microbial causes, solutions and mitigation strategies with the aim of providing an integrated health‐environment‐humanitarian ecosystem perspective to promote a more systems approach to reducing human suffering.

## 3 Microbial Barriers to Infectious Diseases

### 3.1 The Scale of the Problem of Infectious Diseases

Infectious diseases constitute nearly 30% of global disease burden (https://ourworldindata.org/burden‐of‐disease). This amounts to an estimated 830 million DALYs globally, 704 million of which are associated with 85 pathogens (IHME Pathogen Core Group 2024). Approximately 15–17 million deaths/year—25% of deaths due to all causes are estimated to be due to communicable diseases. Infectious illnesses also have considerable impacts on individual earnings and workplace productivity and costs. 50%–60% of all workplace absenteeism is estimated to be due to respiratory infections or gastroenteritis. Approximately 500 million non‐influenza viral respiratory tract infections are estimated to occur each year in the USA, resulting in 70 million lost workdays. In France and Germany, lost productivity related to infectious illnesses in the workplace was found to have cost an estimated $US10–15 billion per year (Blanchet Zumofen, Frimpter, and Hansen 2023; Hansen, Zimmerman, and van de Mortel 2018). Gastroenteritis and respiratory infections in children are the major causes of school absenteeism. Moreover, infections, especially of children, often engender the need for caregivers, with the corresponding loss of their earnings and workplace productivity. Some infections can be either chronic or have sequelae that exacerbate all of these burdens.

### 3.2 Infectious Diseases Are Typical Ecological Interactions: Healthcare Consists of Ecological Interventions and Raising Barriers

Infections are normal ecological interactions in the biosphere: competition for resources, organismal wars, the nature of food chains/webs and predator–prey interactions, and the cycle of birth and death that recycles biological resources along the generations that occur among all free‐living organisms (see also Anderson 1991; Pitlik and Koren 2017; https://asm.org/articles/2024/june/pathogenesis‐not‐a‐trait‐its‐an‐outcome). These battles involve diverse innate defences and weaponry, including antibiotics, and are influenced by external environmental factors. To confront the challenges of human infections, we have learned to manipulate a number of these natural parameters, in addition to instituting a range of technical processes that do not occur naturally, to create new or elevate existing barriers that reduce infections. Some of these manipulations have their roots in traditional medicine practices developed by early civilisations. For example, although antibiotics were discovered and characterised only at the beginning of the last century (e.g., Hutchings, Truman, and Wilkinson 2019), the use of antibiotic‐containing preparations of herbs and other materials in infection prevention and treatment by humans was practiced as early as 2500 BC (https://www.sciencelearn.org.nz/interactive_timeline/15‐antibiotics‐and‐antimicrobial‐resistance‐a‐timeline). It is also worth noting that exploitation of antibiotics to counter infections is not restricted to humans: there are a number of animals and insects that employ antibiotic‐producing microbes to protect vulnerable offspring from infection by pathogens (e.g., Currie et al. 1999; Martín‐Vivaldi et al. 2014; Kaltenpoth et al. 2014), an ecological practice that goes back in animal evolution to the Cretaceous age (ca. 145 My BP).

It is important to note that ecological manipulations in clinical medicine do not always aim to raise barriers; sometimes, they aim to lower them. Examples include the use of blowfly larvae for debridement of necrotic tissue of wounds (maggot therapy): in this case, the necrotic tissue serves as a barrier to body defences where pathogens can proliferate, so its removal by maggots cleanses the wound and allows innate defences to function more effectively. Medicinal leech therapy can be used for a number of different goals but in general to lower functions such as inflammation in inflammatory diseases, coagulation in cases of thrombosis, etc. (Abdualkader et al. 2013; Sig et al. 2017; https://biology.anu.edu.u/research/research‐stories/leeches‐modern‐medicine).

### 3.3 Body Surface Barriers and Their Breaching: Portals of Entry

In order to infect a host and cause disease, a pathogen must be transmitted to, and either colonise—establish themselves and multiply on surfaces of—and/or invade the host. Colonisation and invasion occur via portals of entry: epithelial surfaces. The surfaces of our bodies are covered by a protective cellular barrier—the epithelium—which includes the skin and the mucosal surfaces of the airway, the gastrointestinal and genito‐urinary tracts and ocular surfaces. The epithelium impedes the breaching of body surfaces by microbes and other chemical and physical threats, and is the first line of defence against pathogen attack.

The epithelial barrier itself is covered by microbes—the microbiome—which is designated the ‘second skin’, because it also acts as a physical barrier and protects against colonisation of and invasion by pathogens (Kim, Covington, and Pamer 2017; Byrd, Belkaid, and Segre 2018; Eisenstein 2020; Panwar, Sequeira, and Clarke 2021; Harris‐Tryon and Grice 2022; McCallum and Tropini 2024; Wu and Yao 2024) by leaving little surface area for colonisation by new microbes, and by fighting off those that would invade its territory.

As the primary portal of entry for pathogens, epithelial surfaces are armed with a comprehensive array of immune defences, both innate immunity, which has broad specificity and involves *inter alia* inflammatory processes, clotting factors, phagocytes, complement, cytokines and antimicrobial proteins and peptides, and adaptive immunity, which are dedicated to protecting the epithelial barrier (Moens and Veldhoen 2012). The surface immune barrier is the third line of defence. Moreover, the epithelium and microbiome also act as (bio)chemical barriers by independently and interactively producing an array of substances, that either inhibit ‘enemies’ or promote the growth of microbiome ‘friends’ (Mukherjee and Hooper 2015; Zhang, Merana, et al. 2022). These chemicals include antimicrobial lipids and fatty acids, microbially produced compounds such as iron chelating molecules and, in skin, immuno‐modulatory tryptophan derivatives, and acidifying substances that lower surface pH and are responsible for creating and maintaining the ‘acid mantle’, which plays key roles in skin barrier function (Elias 2015). Epithelial chemistry constitutes a fourth line of defence. In turn, the chemical and immune defences shape, protect and nurture the microbiome. In addition, some epithelial surfaces may be ‘patrolled’ by macrophages that eliminate pathogens and other microbes that are not recognised as indigenous. All of the body surface lines of defence, though largely distinct in terms of actors, are highly interactive and integrated and coordinate responses to threats, especially barrier breaches.

In general, the skin is the most robust barrier (Eisenstein 2020) because a major function is to keep foreign agents out. The internal epithelia are more vulnerable because their function is also to take in—to serve as a portal for—environmental materials, like food and water (gastrointestinal tract) or oxygen (airways). Ensuring that portal activities of internal epithelia are highly selective for needed materials, while keeping enemies out, is more challenging. Some internal epithelia also have an additional defence: production of sticky mucus that binds foreign materials, including microbes, and that is continuously swept to the outside: a conveyer belt physically removing unwanted agents (Turner 2009; Hansson 2012; Song, Chai, et al. 2023).

### 3.4 The Gastrointestinal Tract Epithelial Barrier

The intestine, with an estimated surface area of 32 m^2^, is an important portal of entry for food‐ and water‐borne pathogens. Its epithelial barrier function is largely governed by the integrity of the intercellular ‘tight junctions’, and the other defences mentioned above. Pathogens have evolved mechanisms to breach intestinal defences. For example, *Entamoeba histolytica* specifically binds to mucus via a Gal‐binding lectin, and subsequently produces a cysteine protease that degrades mucin and allows invasion (Solaymani‐Mohammadi and Petri Jr. 2008). Other pathogens penetrate the intestinal barrier either opportunistically, by taking advantage of increased gut epithelial permeability (‘leaky gut’), or actively, by creating increased permeability, or though diverse means of invasion of the epithelium (Kim et al. 2010).

### 3.5 The Respiratory Tract Epithelial Barrier

Although the airways also have a mucus layer which serves a similar function of sweeping pathogens and other particulates to the exterior via the mouth (and thereafter the gastrointestinal tract) and nose, it differs in two important but linked respects, namely the airways are a cul‐de‐sac, not a thoughway, and under certain circumstances mucus can be produced in excess which can clog the airways and hinder pathogen clearance, as is experienced in respiratory infections and diseases like cystic fibrosis (‘mucoviscidosis’).

### 3.6 Endogenous and Exogenous Pathogens

Sources of infections can be either endogenous—transmission is not necessary, although there will have been a transmission event at some point before the pathogen became endogenous—or exogenous, which involves transmission via something external to the person who becomes infected.

### 3.7 Raising/Creating Barriers to Infections: Endogenous Pathogens

Healthy microbiomes typically contain small populations of facultative pathogens, microbes such as *Clostridioides difficile*, that do not generally cause disease because their numbers and hence activities are held in check by ecological controls operating in the microbiomes of healthy individuals, and do not therefore reach infective dose levels. Such controls may be lowered by events that cause microbiome dysbiosis, such as antibiotic treatment, and/or in immune‐compromised individuals, allowing pathogen numbers to increase to infective dose levels that enable a pathogen to overwhelm our defences and initiate disease. Microbial technologies to lower pathogen population levels, and thereby raise barriers to disease, include interventions such as probiotics and microbial transplants to increase gut microbiota diversity and the population levels of beneficial microbes.

### 3.8 Raising/Creating Barriers to Infections: Exogenous Pathogens and Their Transmission

Exogenous pathogens require transmission among hosts and can be acquired by air, water, food, physical contacts, insect vectors, physical injuries‐wounds and invasive health interventions, the latter three of which involve breaching our natural surface barriers.

As a consequence of our inherent barriers to infection, most pathogen transmission events do not lead to disease. In healthy individuals, a large number of pathogenic organisms are needed to overcome our barriers. This number is designated the infective dose and varies according to the pathogenicity of the microbe. Although there are some pathogens, such as shigellae, whose infective dose is low, that is, a few organisms are able to initiate an infection, most pathogens, like salmonellae and vibrios have infective doses in the thousands or millions (Kothary and Babu 2001). Thus: infections by exogenous pathogens have both a qualitative component—the transmission event—and a quantitative component—the number of infectious agents in the transmission event in relation to the infective dose. Raising barriers to such infections involves interventions targeting both of these components.

### 3.9 Challenge 1: Settings With High Pathogen Burdens and/or Highly Susceptible Populations

High concentrations of pathogens are found in certain settings, which translates into high probability of infective dose transmission to susceptible individuals. The classic and historically one of the most important examples is water contaminated with human wastes. The seriousness of the disease impact of contaminated water led to the development of wastewater treatment processes which substantively reduce pathogen loads of incoming wastes.

**Microbial technologies that raise barriers against water‐transmitted disease**

Wastewater treatment is a key, largely microbiological process that reduces the environmental load and transmission of human faecal pathogens and constitutes a major barrier to infections transmitted by water, either directly consumed by drinking or indirectly taken in during washing, swimming, etc. Drinking water is usually subjected to microbiological monitoring, treatment and disinfection (Pluym et al. 2024) to reduce water‐borne infections further and lower the intake of toxic chemicals. A recent study estimates that improving access to safely managed water, sanitation and hygiene services would prevent an additional 1.4 million deaths and 74 million DALYs each year (Wolf et al. 2023). Both types of barrier can be breached by stormwaters, poor maintenance, and accidents and, in the case of drinking water treatment, by the development of biofilms housing pathogens such as *Legionella* in water supply piping (Mondino et al. 2020).

Other settings where pathogens concentrate, such as hospitals, or where hygiene conditions are suboptimal, such as may be found in some disadvantaged communities, refugee camps, informal settlements, slums, care homes, nurseries, war zones, etc. are more challenging, as are close contacts with animals infected with zoonotic pathogens. Surveillance in such settings must be top priority because epidemics can easily break out, and speed is of the essence to contain them. Concerted efforts are needed to provide the necessary expertise and logistics in particularly vulnerable, low‐resource settings. In any case, it is important to avoid situations where pathogens may concentrate, such as swimming in confined freshwaters with high densities of rodents (*Leptospira*; Haake and Levett 2015) and waterfowl (*Cryptosporidium*, *Giardia*; Kuhn, Rock, and Oshima 2002).

Hospitals are particularly problematic environments for infections by resistant pathogens because they contain high populations of people with low barriers to infections due to disease and treatment issues, such as receiving immune depressants following transplantations, are high‐traffic environments where large numbers of people introduce all manner of microbes, and places where diverse pharmaceuticals, including antibiotics, and disinfectants are deployed. These conditions select a robust ‘hospital microbiome’ in which antibiotic and disinfectant resistances are common and where facultative pathogens that may not cause disease in healthy individuals are able to produce life‐threatening infections in vulnerable patients. Of particular concern are the antibiotic‐resistant ESKAPE pathogens: *Enterococcus faecium* , *Staphylococcus aureus* , *Klebsiella pneumoniae* , *Acinetobacter baumannii* , *Pseudomonas aeruginosa* , *Enterobacter* species (De Oliveira et al. 2020; Ayobami et al. 2022; Denissen et al. 2022; Miller and Arias 2024), which are able to colonise high‐touch surfaces, like handles, and thence be transmitted to almost anyone, including patients, hospital professionals and visitors, the latter two of which also serve as agents of transmission to patients. Earlier effective barriers to hospital‐acquired (nosocomial) infections included hygiene measures based on comprehensive disinfectant use, but their use is now experiencing diminishing efficacy, not least because hospital microbiomes typically include disinfectant‐resistant AMR pathogens. However, the application of probiotic bacteria and bacteriophages to surfaces that carry high loads of pathogens can be an effective means of reducing such loads and hence their ability to deliver infective doses. This is strategy being tested and rolled out in hospitals and elsewhere.

Fomite (surfaces)‐to‐person and person‐to‐person contacts are in general significant routes of transmission of infections. Contact transmission can be reduced by avoiding hand‐shaking/kissing/hugging when greeting, and by the use of condoms in sexual encounters. Other barriers include disinfection, sanitation and hygiene but, as already mentioned, pathogens are becoming increasingly tolerant‐resistant to disinfectants, so these barriers are becoming less effective (Tong et al. 2021).

Food is a major vehicle for pathogen transmission via the faecal‐oral route, either contaminated during the supply chain or during preparation. While basic hygiene practices are the mainstay barriers against food contamination, diverse means of processing food materials and food safety controls are key to preventing the consumption of contaminated food.

Respiratory infections are common (https://immunizationdata.who.int/, https://www.ecdc.europa.eu/en/data‐dashboards‐and‐databases) and transmitted by breathing air with a high pathogen load. Barriers include air filtration systems, sometimes coupled with disinfection (e.g., by UV irradiation), use of masks and personal distancing, avoidance of large gatherings in confined spaces, especially in times of epi−/pandemics, and pathogen dilution with fresh air by ventilation.

Breaches of the surface barriers can be non‐specific, like those caused by surgery, transfusions, catheters and other invasive clinical interventions, physical wounds and animal bites, all of which become potential portals of entry for pathogens.

Just as humans have evolved sophisticated barriers to infection, so have microbial pathogens evolved ways and means of circumventing or countering our innate defences: their specific mechanisms to breach surface barriers (Hornef et al. 2002). One of the most effective of these is pathogen exploitation of blood‐sucking insect vectors (that are often also intermediate hosts for the pathogens), like mosquitoes (https://ukhsa.blog.gov.uk/2024/07/26/how‐you‐can‐help‐us‐resist‐the‐tiger‐mosquitos‐conquest‐of‐europe/) that can transmit dengue (https://www.weforum.org/stories/2024/11/dengue‐fever‐outbreak‐climate‐change/?utm_source=sfmc&utm_medium=email&utm_campaign=2839332_AgendaWeekly‐8November2024&utm_term=&emailType=Agenda%20Weekly) and malaria, and ticks that can transmit Lyme borreliosis and tick‐borne encephalitis. Such vectors inject pathogens directly into the blood stream during the blood meals needed to provide the nutrients for egg production. Insect vector‐mediated transmission circumvents all the surface barrier defences. Examples of microbial technologies available to raise barriers in these cases are, on one hand, vaccination (e.g., to prevent tick‐borne encephalitis) and antibiotics (e.g., to treat *Borrelia*) and, on the other, the deployment of *Wolbachia* to reduce transmission of pathogens by female mosquitos.

### 3.10 The Wolbachia Technology Barrier

*Wolbachia* is a bacterial endosymbiont of around 50% of the arthropods, and also of many nematodes, that profoundly influences host physiology and behaviour. *Wolbachia* particularly impacts host reproduction in ways that favour its own propagation and transmission among host populations. One impact on the host includes reducing host mortality from microbial infections by inhibiting the transmission and virulence of pathogens the host may carry, some of which like dengue and Zika virus also infect humans. In other cases, *Wolbachia* endosymbionts have become intricately intertwined with host biology and are essential for host survival and reproduction, as is the case for the nematode *Onchocerca volvulus*, the causative agent of river blindness. These interactions between *Wolbachia* and its hosts can be exploited to fight disease and have already triggered explosive growth in *Wolbachia* technology directed at reducing insect‐ or nematode‐vectored infections of both humans and crop plants.

### 3.11 Raising/Creating Barriers to Infections: Prophylaxis

Since its inception, vaccination has saved countless lives and protected many more from disease (e.g., considering just one vaccine, it is estimated that the measles vaccine saved 31.7 million deaths over a 20‐year period https://www.who.int/news‐room/spotlight/history‐of‐vaccination/history‐of‐measles‐vaccination; Venkatesen 2022; see also Nandi et al. 2019). There are a range of vaccines available to prevent many infectious diseases (e.g., https://immunizationdata.who.int; https://vaccineknowledge.ox.ac.uk/home; https://immunizationdata.who.int/; Croucher 2024). Immunisation with vaccines supports the initial host response to infection by significantly elevating the barrier function of adaptive immunity against specific pathogens. Priming the immune system through vaccination to achieve a rapid and massive response to infection can not only protect the individual, but also the population, through attainment of ‘herd immunity’ (Fine, Eames, and Heymann 2011).

The ability to spread in a totally susceptible population, quantified as the R_0_ number—the average number of people acquiring an infection from a diseased individual—is a pathogen‐specific characteristic (https://www.cebm.net/covid‐19/when‐will‐it‐be‐over‐an‐introduction‐to‐viral‐reproduction‐numbers‐r0‐and‐re/#:~:text=If%20R0%20is%20less,the%20data%20that%20inform%20it): for example, the R_0_ number for measles is around 15 (Guerra et al. 2017). Immunisation (and recovery from infection) reduces the proportion of susceptible individuals in a population, and we then instead consider the effective reproduction number, R_e_, which will reduce compared to the R_0_ value the more individuals are immunised or have recovered from natural infection (https://www.cebm.net/covid‐19/when‐will‐it‐be‐over‐an‐introduction‐to‐viral‐reproduction‐numbers‐r0‐and‐re/#:~:text=If%20R0%20is%20less,the%20data%20that%20inform%20it). Once a particular threshold of unsusceptible individuals is reached, for example 95% in the case of measles, the pathogen fails to transmit effectively, and there is a decline in disease incidence. This threshold, which is dependent on the R_0_ number, is when herd immunity is achieved. Crucially, herd immunity confers a significant degree of indirect protection on unvaccinated individuals (https://vaccineknowledge.ox.ac.uk/herd‐immunity#People‐who‐depend‐on‐herd‐immunity), which is particularly important as populations age and numbers of people with lower immunity, for example, due to treatment with immunosuppressants, co‐ and polymorbidities, etc., increase.

Impressively and importantly, vaccination campaigns aimed at achieving herd immunity have eradicated a number of deadly infections, either globally (smallpox, rinderpest) or regionally (polio, rabies) and have the ability to eradicate more (e.g., guinea worm, malaria), if socio‐economic parameters will allow (https://ourworldindata.org/eradication‐of‐diseases; https://asm.org/Articles/2024/September/Polio‐s‐Last‐Stand‐The‐Global‐Fight‐for‐Eradicatio?utm_medium=email&utm_source=rasa_io&utm_campaign=newsletter). This represents the alleviation of an enormous health burden. Although rinderpest was a deadly infection of cattle (cattle plague), not of humans, it had an enormous impact on human health because outbreaks caused famines responsible for millions of deaths and created poverty among farmers (https://ourworldindata.org/how‐rinderpest‐was‐eradicated).

However, vaccine hesitancy (Larson, Gakidou, and CJL 2022) tends to reduce vaccine coverage, so that herd immunity is no longer attainable for some infections and the vulnerable are not protected. In 2019, the WHO listed vaccine hesitancy as one of the 10 threats to global health (https://www.who.int/news‐room/spotlight/ten‐threats‐to‐global‐health‐in‐2019). Even when vaccination levels that provide herd immunity are achieved it can break down because, due to vaccine hesitancy within certain groups (Jäckle and Timmis 2023), vaccination levels are not uniform across communities and pockets of unvaccinated individuals can create mini‐epidemics that can jump to other pockets (Peeples 2019). In the context of microbial technologies to raise barriers against disease, vaccine hesitancy is a significant opposing barrier.

### 3.12 Raising/Creating Barriers to Infections: Therapeutics

Treatment of infections often involves *inter alia* the administration of antibiotics which, if the causative agent is sensitive, can be extremely effective, add an additional weapon to the natural defences of the infected individual, and tip the balance of the ongoing ecological war in favour of the host.

### 3.13 Challenge 2: Antimicrobial Resistance

Antimicrobial resistance (AMR) ‐ has been increasing alarmingly in recent years and is now predicted to restore infectious diseases as the main cause of human mortality, as was the case in the pre‐antibiotic era (Ventola 2015a, 2015b; Coque et al. 2023). One report forecasts that by 2050 infectious diseases will be responsible for 100 million deaths annually and result in a cumulative loss of economic output worth US$ 100 trillion (https://amr‐review.org/sites/default/files/160525_Final%20paper_with%20cover.pdf; Naghavi et al. 2024). AMR is considered by the WHO to be one of the most serious threats to human medicine, a concern that was brought into sharp focus by a 2019 study which attributed 1.27 million deaths worldwide directly to AMR and another 4.95 million deaths as associated with AMR (Murray et al. 2022).

New antibiotics effective against current AMR pathogens are urgently needed but the pipelines of new candidates are almost dry. The revitalisation of antibiotic discovery programmes is hugely important and urgent (e.g., Timmis et al. 2014; Anderson et al. 2023; Birkelbach et al. 2024; Brüssow 2024).

However, while there is indeed cause for alarm, and the currently increasing trajectory of morbidity and mortality caused by AMR pathogens constitutes a crisis, this prediction is based on modelling studies that fail to take into account the potential influence of a major revitalisation of antibiotic discovery programmes and the development of alternative microbial technologies based on ecological barriers. While drug prospecting has traditionally focused on microbial sources and continues to do so with increased emphasis on poorly investigated microbes, including uncultured microbes, and on expression of silent or inactive microbial biosynthetic pathways, other sources such as human and animal antimicrobial peptides are also receiving increasing attention. However, in addition to new but classical antibiotics, a number of other options are being actively investigated. These include co‐therapies of antibiotics and compounds targeting functions vital to antibiotic resistance (Laborda et al. 2024; Wu, Huang, and Xu 2024; Wan et al. 2024; Xu and Lin 2024) and the use of pathogens of pathogens, such as bacteriophages and bacteriovorous bacteria, like *Bdellovibrio*, surface‐engineered bacteria (Dahlsson Leitao, Ståhl, and Löfblom 2024), anti‐pathogen and anti‐AMR CRISPR‐Cas systems (Bikard et al. 2014; Derollez, Lesterlin, and Bigot 2024), therapeutic antibodies/nanobodies that inactivate pathogens or toxins, use of antimicrobial nanomaterials (Arora, Lashani, and Turner 2024) and 'nanobiotics' (Chakraborty et al. 2022; Lashani et al. 2024), and microbiota transplants (Bratkovič et al. 2024; Carratalá et al. 2024). Bacterial therapeutics‐bacteria engineered to inactivate inter alia pathogens, their virulance products, and antibiotic‐inactivating enzymes, or to deliver therapeutic payloads at sites of disease‐would seem to have signficant potential (Srivastava and Lesser 2024). These various approaches, and others, based on microbial technologies, have yet to prove their ability to achieve significant reductions in morbidity and mortality caused by infections by AMR pathogens, but their development and testing needs to proceed at pace. Moreover, new technologies are available and in development that increase the speed and accuracy of antibiotic sensitivity testing, thereby reducing the time during which potentially ineffective antibiotics are administered. These improve disease outcomes and reduce selection pressure for AMR. Importantly, monitoring AMR and its evolution by PCR, sequencing, etc., will be crucial to targeting and raising barriers against it.

It is important to note that vaccines also play a crucial role in combating antimicrobial‐resistant (AMR) pathogens by preventing infections, reducing the reliance on antibiotics and lowering the selection pressure for resistance (Bloom et al. 2018; Sevilla et al. 2018). New vaccines targeting AMR pathogens will be able to directly reduce the proliferation and spread of resistant strains. Recently developed generic technologies to generate attenuated live vaccines have focused on the creation of D‐glutamate‐dependent variants of pathogens (Cabral et al. 2017), and variants dependent upon an unnatural amino acid (Pigula et al. 2024), neither of which proliferate significantly in the host so do not cause disease, but which elicit protective immune responses. Such vaccines were shown to be effective against challenge by multi‐resistant *Acinetobacter baumannii* , *Pseudomonas aeruginosa* and *Staphylococcus aureus* in animal models, so have considerable promise as an essential technology in the broader strategy to combat AMR. Such vaccines should contribute not only to individual and public health, but also to the sustainability of effective antimicrobial therapies.

In addition to the development and deployment of new technologies, there must also be accompanying measures which ensure the reduction in non‐clinical use of antimicrobial agents, to lower environmental selection pressures for evolution and transmission of AMR, and provide the education which will be required to achieve this (e.g., Calvo‐Villamañán, San Millán, and Carrilero 2023).

### 3.14 Challenge 3: Chronic and Recurrent Infections

Sometimes, even when natural defence barriers are augmented with antibiotics, pathogen clearance is not achieved and infections flare up again, particularly in patients with indwelling devices (one study recorded that > 90% of hospitalised patients have an indwelling device: Chen, O'Malley, and Chopra 2021). This may have different causes, including genetic defects in vital barriers, such as pathogen clearing processes, as is the case in cystic fibrosis (Ribeiro et al. 2023), pathogen surface variability that prejudices the efficacy of pathogen recognition by body defences, pathogen localisation in body sites that are poorly accessible to host defences, pathogen creation of protective subcellular location structures and extracellular colony structures like biofilms (Bjarnsholt 2013), and development of persister states in which dormancy is induced *inter alia* by antibiotic treatment or phages (Fernández‐García et al. 2022; Fernández‐García et al. 2024).

### 3.15 Raising Barriers to New Infections With the Aid of Artificial Intelligence

Microbial pathogens, like all members of the biosphere, evolve to adapt to changing environments. Unlike most visible members of the biosphere, microbial pathogens may evolve very rapidly because most can have very short reproduction times under certain conditions. Evolution includes developing new host specificities, in particular for humans in the case of animal pathogens, and increased virulence. As the 'space' of evolutionarily space for pathogens becomes better known, it will become easier to predict using artificial intelligence what new infection threats may evolve, and hence to be better prepared and take effective measures to counter them (Danchin 2024).

### 3.16 Creating Barriers to Infectious Amyloid

Neurodegenerative diseases, like Alzheimer's and Parkinson's (a) are major, essentially unpreventable and untreatable lethal diseases of old age, (b) involve progressive build‐up of denatured protein—amyloid—plaques in the brain that inhibit normal neurological functions, and (c) are similar in some ways (progressive amyloid build‐up) to prion diseases. There is an association between Alzheimer's disease and the gut microbiota (Grabrucker et al. 2023; Marizzoni et al. 2023; Williams et al. 2024). Prion diseases of animals cause transmissible spongiform encephalopathies (TSEs), such as bovine spongiform encephalopathy (BSE; mad cow disease) in cattle and scrapie in sheep (Prusiner 1998). BSE can be transmitted via infected animal products to humans causing variant Creutzfeldt‐Jakob disease (vCJD). A characteristic of the denatured protein in amyloid plaques is the failure of on‐site protein quality control systems (i.e., proteases and chaperones) to degrade and recycle it. Prion amyloid is infective, that is, acts as a template to promote misfolding of similar proteins, and thereby reproduces.

Bacteria, including gut bacteria, are known to produce and release amyloid proteins (Giraldo 2020), for example, in biofilms, and a possibility that has been raised is that one potential aetiology of human neurodegenerative disease is that gut bacteria‐produced amyloid migrates to the brain and acts as a template that catalyses initiation and propagation of human amyloid in the brain (Jain 2024; Elkins, Jain, and Tükel 2024). One potential strategy being explored to reduce the incidence of amyloid‐caused neurodegenerative disease is the removal of amyloid‐producing microbes, or of the secreted amyloid itself, in the gut microbiota. Another relates to the fact that gut microbiota‐produced metabolites have pro‐inflammatory activity on neurons and glia (e.g., see Cattaneo et al. 2017), which creates a window of opportunity to intervene in microbial metabolic networks to down‐regulate production of such metabolites. Prion proteins released into soils from dead animals are long lived and contaminated soils may represent a source of new infections. Moreover, plants are known to take up prion proteins via their roots, transport them to their aerial parts, and thence transmit them to herbivores (Carlson et al. 2023). One possibility to confront this risk may be the deployment of microbial proteases able to degrade animal prions and targeting contaminated soils (and their run‐off waters), and/or their ecosystem engineers, like earthworms (Nechitaylo et al. 2010; Pritzkow et al. 2021) and plant rhizospheres (Elkins, Jain, and Tükel 2024).

## 4 The Microbiome Barrier

The human microbiome consists primarily of microbial populations—microbiota—on the different body surfaces: skin, oral cavity, gastrointestinal (GI) tract, respiratory tract, ocular surface and genital tract. Some internal tissues/organs may also be colonised temporarily (e.g., blood following a cut or graze, after brushing the teeth; see also Tan, Ko, et al. 2023; Michán‐Doña, Vázquez‐Borrego, and Michán 2024) or longer term (e.g., tumour colonisation: Nejman et al. 2020). Each body site is characterised by unique physiological conditions that select microbiota of different compositions with different activities and interactions with host tissues—functionalities—and health consequences (McCallum and Tropini 2024). Most of these interactions are either positive or essential: they are the basis of the goods and services the microbiome contributes to the human‐microbiome partnership.

### 4.1 Dysbiosis

Studies in model animals and humans suggest that both wellness and ill health may be influenced, sometimes determined, by the microbiota, especially when the ‘normal’ microbiota is perturbed in its composition, either by inherent or environmental factors, or by interventions like antibiotic treatment (Kim, Covington, and Pamer 2017). Disequilibrium of the microbiome is termed dysbiosis and may be associated with perturbed provision of microbiome‐supplied goods and services, including barrier functions (Pitlik and Koren 2017). Dysbiosis is frequently characterised by a lower microbial diversity, increased levels of microbes considered to be potentially harmful, and lower levels of microbes considered to be beneficial and thought to contribute to barriers against disease, especially disease caused by microbes (Hrncir 2022). A number of factors, including the genetic make‐up of the host, ageing, lifestyle habits including smoking, alcohol and substance abuse/addiction (García‐Cabrerizo and Cryan 2024), exposure to environmental pollutants including agrochemicals and emerging pollutants, infections and other diseases and clinical therapies, are associated with a shift in a normal, diverse microbiome towards one that is less diverse and tending to the dysbiotic state. However, classifying a microbiota as healthy or dysbiotic is not straightforward (Brüssow 2020).

### 4.2 Gut Microbiota

The gut microbiota is the best‐studied component of the microbiome and has a pervasive influence on the physiology and well‐being of the host. The GI tract acts as a conveyor belt with food taken in at one end and the remains, microbes and the products of host:microbe activities expelled at the other. During the passage of food through the gut, enzymes produced by both the host and its microbiota digest the food‐derived macromolecules producing metabolites that are taken into the body and used for metabolism to generate energy and produce materials the host needs to grow and maintain and regulate essential functions. Microbial fermentation processes also generate energy and nutrients needed for proliferation of the gut microbes, and metabolites, which are mainly short‐chain fatty acids that are important mediators in host–microbe interactions. As a result of their active proliferation in the gut, microbes make up around 50% of the faecal biomass (Sender, Fuchs, and Milo 2016).

As a consequence of these digestive activities, a vast array of metabolites are produced beyond short‐chain fatty acids, some of which have a profound diet‐dependent influence on other organs of the body and their function, and hence host physiology and well‐being (Valdes et al. 2018; Nicolas and Chang 2019; Morris et al. 2017), via multiple signalling pathways that influence gene regulation and protein expression, amino acid metabolism and functioning of the immune system. Some of the metabolites have hormone‐like activities (the gut microbiota has been called the second endocrine system; Clarke et al. 2014; Neuman et al. 2015; Rastelli, Cani, and Knauf 2019) that influence hormonally controlled processes. Yet other metabolites influence the activities of the enteric nervous system and thence the brain. The metabolic processes that take place in the gut, particularly those mediated by the gut microbiota, and modulated by dietary intake and internal and external influences, are thus networked with the activities of other organs and play key roles in their proper functioning and homeostasis. These are the gut–organ axes, such as the gut‐oral axis (Kunath et al. 2024), the gut‐brain axis (de la Fuente‐Nunez et al. 2018; Margolis et al. 2021; Smith and Wissel 2019; Schneider et al. 2024; https://www.sciencedirect.com/book/9780323999717/the‐gut‐brain‐axis), the gut–liver axis (Pabst et al. 2023), the gut‐heart axis (Bui et al. 2023), the gut–lung axis (Dang and Marsland 2019), the oral–lung axis (Garmendia and Cebollero‐Rivas 2024), gut‐skin axis (Guo, Luo, et al. 2024), gut‐bone axis (Behera et al. 2020), gut–immune axis (Sanz et al. 2015; Dinakis, O'Donnell, and Marques 2024), communicating (often bidirectionally) via metabolic, humoral, immune, endocrine or neural pathways (Ahlawat, Asha, and Sharma 2021).

The gut microbiota also interacts with and metabolises therapeutic drugs with profound consequences (Alexander et al. 2017). Such interactions include degradation of drugs before they can be taken up by the host, and chemical modification that may result in enhanced or reduced potency, uptake or toxicity, resulting in reduced or elevated drug efficacy and/or drug‐related adverse reactions. These interactions, which can have a major effect on therapy‐disease outcomes, differ among individuals with different microbiomes, and may differ within an individual according to diet or other parameters. A major preoccupation of future precision medicine will be to improve drug therapy efficacies and disease outcomes by optimising drug‐microbiome compatibility‐functionality (e.g., see Garmendia and Cebollero‐Rivas 2024).

### 4.3 Gut Microbiota Dysbiosis

The gut microbiota provides key physical and chemical barriers to colonisation and invasion by pathogens (Moens and Veldhoen 2012; Caballero‐Flores, Pickard, and Núñez 2023). Principal mechanisms include competition for space and nutrients, production of short‐chain fatty acids and antimicrobial agents, and release of bacteriophages (Ducarmon et al. 2019; Ramsteijn and Louis 2024). One consequence of gut microbiota dysbiosis can be increased permeability of the gut epithelium—‘leaky gut’—due to structural changes in the intercellular tight junctions (Bischoff et al. 2014; Pickard et al. 2017; Di Vincenzo et al. 2024). Leaky gut is associated with a number of metabolic and immune diseases, caused for example by lipopolysaccharide endotoxins crossing the gut barrier and entering the portal circulation (Gäbele et al. 2011), causing inflammatory diseases (Di Vincenzo et al. 2024). Dysbiotic gut microbiota have also been associated with intestinal and systemic diseases such as type 2 diabetes, obesity, hypertension, colorectal cancer, non‐alcoholic liver disease, cardiometabolic diseases, malnutrition, autoimmunity, allergies and asthma (Fan and Pedersen 2021; Hrncir 2022; Tan, Ko, et al. 2023; Tan, Taitz, et al. 2023).

In addition to influencing overall health, the gut microbiota is also linked to the ageing process through its impact on immune system modulation and disease vulnerability (O'Toole 2024). In most individuals, the gut microbiota changes progressively with age, resulting in changes in microbiota‐produced metabolites that influence inflammatory processes and increase gut leakiness. These changes induce systemic inflammation, which when associated with ageing is termed ‘inflammaging’, and is associated with overall loss of fitness and poorer health in the elderly (Badal et al. 2020; Ragonnaud and Biragyn 2021).

### 4.4 Microbiota Therapies

Given that the gut microbiota produces a vast array of metabolites, some of which have profound effects on host physiology and well‐being, understanding the metabolic capabilities and nutrient requirements of gut microbial communities will open new opportunities for targeted microbiota prophylaxes and therapies (Valdes et al. 2018; Vargason and Anselmo 2018; Nicolas and Chang 2019; Turjeman and Koren 2021; Kostic et al. 2024; McCallum and Tropini 2024). Indeed, microbiomes are becoming a key component of precision medicine and have medical applications as biomarkers useful for diagnosis, patient stratification, prognostics, surveillance, and as a target for interventions (e.g., see Zeevi et al. 2015). Significant advancements in synthetic biology and microbiome ecology have spurred the development of microbiota editing for prophylactic and therapeutic purposes.

The principal microbiome manipulation interventions focus on introducing or favouring the growth of desirable microbes, such as strains of *Bifidobacterium, Lactobacillus* and *Blautia* (https://ods.od.nih.gov/factsheets/Probiotics‐HealthProfessional/; Sen et al. 2022), restoring community diversity and functionality, or *microbiota editing*: selectively reducing the competitiveness of detrimental microbes and opportunistic pathogens, through dietary interventions, prebiotics, probiotics and synbiotics, faecal microbiota transplantation, phage therapy, live biotherapeutics or microbiome mimetics (Chua et al. 2017; Valdes et al. 2018; Vargason and Anselmo 2018; Gulliver et al. 2022; Sen et al. 2022; Sorbara and Pamer 2022; Berding et al. 2023; Ramsteijn and Louis 2024; Zhang et al. 2024). The promise of successful microbiota applications in medicine is supported by effective faecal matter transplant (FMT) treatment of the otherwise untreatable infection *Clostridioides difficile*‐mediated colitis (Gonzales‐Luna, Carlson, and Garey 2023). Another example of microbiota approaches includes augmentation of butyrate‐producing bacteria in patients suffering from inflammatory bowel disease characterised by a dysbiotic gut microbiota depleted of butyrate producers (Geirnaert et al. 2017). Approaches to address increased gut permeability include the oral administration of normal mucus‐inhabiting microbes coated with polyethylene glycol, to localise the added microbes to the mucus and reinforce its barrier function by stimulating its production (Chen et al. 2024). It has also been suggested that ethanolamine may be a cause of increased gut permeability, resulting from a microbiota with reduced ethanolamine metabolic activity. Probiotic restoration of ethanolamine metabolising microbes restores the normal gut epithelial barrier (Mishra et al. 2023).

### 4.5 Oral Cavity Microbiota

Dysbiosis of the oral cavity microbiota has been associated with periodontitis and caries, and their sequelae, (e.g., tooth/bone loss and pulpitis), and systemic diseases associated with these oral diseases, such as infective endocarditis, atherosclerosis, diabetes, Alzheimer's disease and head and neck/oral cancer (Radaic and Kapila 2021). Moreover, oral dysbiosis‐associated periodontitis has an influence on complications of respiratory diseases, including chronic obstructive pulmonary disease (COPD) and obstructive sleep apnoea (Wen et al. 2022; Molina et al. 2023).

### 4.6 Skin Microbiota

The skin microbiota not only serves as a vital barrier against pathogens but also plays a major role in wound healing and repair processes, through local stimulation of inflammation and immune responses, influencing keratinocyte growth, and blood vessel development, and also skin neoplasia, through production of a purine analogue that inhibits cancer cells (Nakatsuji et al. 2018). It also limits the formation of biofilms. Probiotics and other microbiome‐based approaches are now increasingly evaluated for the management of wound healing (Nakatsuji et al. 2017; Canchy et al. 2023; Yin et al. 2024) and thus the process of rehabilitation, and atopic dermatitis (Vargason and Anselmo 2018).

### 4.7 Respiratory Tract/Airways

The respiratory tract is the largest external surface of the human body, estimated to be around 145 m^2^, most of which is represented by the alveoli whose function is gas exchange between the blood and the external environment: uptake of oxygen for metabolism and excretion of waste carbon dioxide. Other functions of the respiratory tract include smell, phonation‐speech and filtering‐warming‐humidifying intake air. The oxygen uptake surface of the alveoli is of functional necessity vulnerable to damage by small particles and microbes, and especially attack by pathogens (Costantini, Nunzi, and Romani 2022). A major barrier to small particle damage‐pathogen attack is the mucus layer in the respiratory tract which binds particulates and sweeps them out through ciliary action (Abrami et al. 2024). This layer is covered with a microbiota that provides a range of protective functions (Pérez‐Cobas et al. 2023; Garmendia and Cebollero‐Rivas 2024). However, its vulnerability as a portal of entry for pathogens is testified by the frequency of respiratory infections, especially viral infections, suffered by most people every year. Viral infections increase the vulnerability of the airway epithelium and additionally cause dysbiosis of the airway microbiota, therefore lowering the airway defences to attack by other pathogens which can cause secondary infections, such as bacterial pneumonia. This usually requires antibiotic therapy which in turn perturbs the microbiome composition and may cause general dysbiosis. Development of vaccines against viral respiratory pathogens, and microbiota‐based therapies for prevention and treatment of secondary microbial infections of the airways, are urgently needed.

### 4.8 Genital Tract Microbiota

The genital microbiota has a great influence in maintaining reproductive health and protecting from disease. While a healthy vaginal microbiota plays a significant role in fertility (Venneri et al. 2022) and protection against vaginosis and sexually transmitted infections (Lewis, Bernstein, and Aral 2017), the microbiota of the male genital tract (which mainly consists of the urethra and coronary sulcus) is associated with erectile function, semen quality and male fertility (Mändar 2013). There is now evidence that the microbiota exchanged during sexual intercourse has a significant influence on the female microbiota and raises the fitness of sexual reproduction by enhancing sperm movement and survival, as well as egg fertilisation (Mändar 2013). Sexual activity involving a partner with an unhealthy microbiota may increase dysbiosis by reducing the microbial diversity, leading to loss of beneficial bacteria and a rise in harmful ones (Tuddenham, Ravel, and Marrazzo 2021). This may not only lead to bacterial vaginosis or increase the risk of acquiring sexually transmitted infections, including HIV (Toh et al. 2023), but may also result in mental disorders including brain‐fog, irritability, mood changes and anxiety (Nasrallah 2022; Lewis, Bernstein, and Aral 2017). Vaginal microbiome modulation, principally through the application of lactic acid bacteria, is being explored as a therapy for vaginosis (Vargason and Anselmo 2018)

### 4.9 Challenge 4: The Variation in Microbiome Composition and Establishing Causalities

A huge challenge to identifying causalities in microbiota‐associated diseases, in order to design effective microbiota engineering therapies, is the high degree of variation in microbiome composition between individuals, and indeed within individuals over time, and/or related *inter alia* to changes in nutrition, lifestyle and co‐morbidities. This renders statistical analysis challenging and makes recognition and definition of microbiota dysbiosis problematic (Brüssow 2020). That said, advances in animal models, including germ‐free animals, especially the fruit fly, which has a low diversity microbiome and hence is easy to manipulate and interpret results (Grinberg et al. 2022; Dodge et al. 2023), humanised mice (Uzan‐Yulzari et al. 2024), and human organoids (Bozzetti and Senger 2022), can provide significant support for the likelihood of causality of some associations (Aguanno et al. 2022). For example, establishment in mice of gut microbiota from healthy and diseased humans, with reproduction of disease symptoms, may enable identification of putative causal microbes/microbial groups and, in some cases, fulfilment of Koch's Postulates in the mouse model. Reproduction of the pathology/proxies of pathology in human organoids, and mechanistic investigations, may then provide important corroborating evidence which can then be used to design optimal human trials.

## 5 Microbial Technology Barriers to Non‐transmissible Diseases

### 5.1 Leading Causes of Global Burden of Disease

In 2019, the leading causes of burden of disease were cardiovascular diseases (418 million *DALYs*), cancers (247 million DALYs), neonatal disorders (199 million DALYs) and respiratory infections (161 million DALYs). ‘In the poorest regions of the world, childhood and maternal underweight, unsafe sex, unsafe water, sanitation, and hygiene, indoor smoke from solid fuels, and various micronutrient deficiencies were major contributors to loss of healthy life. In both developing and developed regions, alcohol, tobacco, high blood pressure, and high cholesterol were major causes of disease burden’. (Ezzati et al. 2002). Other assessments include *inter alia* air pollution, obesity and drug abuse (see https://ourworldindata.org/grapher/number‐of‐deaths‐by‐risk‐factor; https://www.who.int/data/gho/data/themes/topics/topic‐details/GHO/ncd‐risk‐factors). Although each of these risk factors is listed as a separate parameter, several are obviously overlapping, intertwined and mutually reinforcing (https://www.niddk.nih.gov/health‐information/weight‐management/adult‐overweight‐obesity/health‐risks).

### 5.2 Metabolic Disease

As indicated above, the gut microbiota in combination with nutrition plays a major role in metabolic dysfunctions such as obesity, type 2 diabetes, non‐alcoholic liver disease and cardiometabolic disorders, as well as in inflammatory and autoimmune diseases via the gut–immune axis (Morris et al. 2017). As a consequence, there may be considerable potential for microbiome engineering‐editing for prevention and treatment of such disorders (Sanz et al. 2015; Fan and Pedersen 2021; Wu et al. 2021; Ross et al. 2024; https://www.sciencedirect.com/book/9780323999717/the‐gut‐brain‐axis; see also Lin et al. 2024 and references therein). This potential will grow with the increasing discovery of new metabolic biomarkers (e.g., see Karjalainen et al. 2024) and the identification of microbes playing roles in the production of key gut metabolites.

Obesity, which results from the activities of two body weight regulation systems, energy homeostasis and cognitive‐emotional control (Lister et al. 2023), is considered to be an epidemic (https://www.who.int/news‐room/fact‐sheets/detail/obesity‐and‐overweight). According to one report, 69% of adults in the USA are obese (Busebee et al. 2023). Obesity is associated with multiple health risks that include Type 2 diabetes, cardiovascular disease, cancer, psychiatric issues (https://www.niddk.nih.gov/health‐information/weight‐management/adult‐overweight‐obesity/health‐risks; Valenzuela et al. 2023), and, as a consequence, premature death: 5 million people in 2019 (https://ourworldindata.org/obesity). The economic costs of obesity are staggering: US$ 2 trillion in 2020 predicted to rise to more than US$ 3 trillion by 2030 (https://data.worldobesity.org/publications/WOF‐Economic‐Impacts‐2‐V2.pdf; Okunogbe et al. 2021).

It has been suggested that a contributing cause of the obesity epidemic is the rise in consumption of ultra‐processed foods and sugar‐sweetened beverages (Dicken and Batterham 2024; Piperni et al. 2024; see Monteiro et al. 2017, for a discussion of the NOVA food processing classification). Independently of this nutritional contributor, and availability of an increasing number of weight loss medications (Melson et al. 2024), a number of reports associate microbiome composition with obesity and indicate that microbiome‐diet interventions, coupled with supporting measures that *inter alia* address possible underlying mental‐stress‐anxiety issues, have the potential to raise barriers against and thereby counter obesity (e.g., de Wit et al. 2023; Lister et al. 2023; but see also Sze and Schloss 2016 and Van Hul and Cani 2023).

### 5.3 Cancer

Each year there are 19 million new cases of, and 10 million deaths due to, cancer (Sung et al. 2021). Microbes can be directly associated with the initiation of different types of cancer (https://asm.org/articles/2023/august/cancer‐microbiology‐connecting‐the‐dots#:~:text=Examples%20of%20carcinogenic%20parasites%20include,Click%20image%20for%20larger%20view). The leading cause of liver cancer results from virus infection, especially by hepatitis viruses B and C (HBV and HCV). The HBV vaccine protects against both HBV infection and HBV‐caused liver cancer. Human papilloma virus (HPV) infection through person‐to‐person contact can also cause cancer. The anti‐HPV vaccine, given at age 11–12 before most instances of transmission is highly protective. *Helicobacter pylori* infects the stomach lining and can cause gastric cancer and lymphoma, and increase the risk of colorectal cancer. Antibiotic treatment to eliminate *H. pylori* greatly reduces the risk of such bacterially initiated cancers. Further discoveries of links between infectious pathogens and cancers, and other chronic disease states, are not unlikely (Shi, Li, and Zhang 2024). Antimicrobial treatment of cancer‐triggering bacterial infections comes with a risk, however, namely microbiota perturbation and the possibility of dysbiosis‐induced health disorders, including other cancers (Gao et al. 2020; and see below). To reduce dysbiosis and increase treatment selectivity, bacteriophages against cancer‐causing microbes are being developed for use, also for combination therapies (Dong et al. 2020; Zheng 2019).

In addition to preventing microbially initiated cancers, microbial technologies—either cell factories producing therapeutic agents, or as therapies in of themselves—show considerable promise both for diagnosis (Turjeman and Koren 2021; Chu et al. 2024) and treatment (Luo et al. 2016; Hardebeck et al. 2024; Howell et al. 2024; Wu, Huang, and Xu 2024; Xia and Wu 2024; Xiao et al. 2024; Xu et al. 2024) of non‐microbially caused cancers. The main problem with cancer therapy is the fact that it is uncontrolled proliferation of own tissues, so attacking cancer cells generally results in attack of own cells, of self; discrimination of target and non‐target cells, and hence selective toxicity, is usually low. Classical chemotherapy treatment of cancers exploits the fact that cancer cells proliferate more rapidly than most non‐cancer cells (https://www.cancer.org/cancer/managing‐cancer/treatment‐types/chemotherapy/how‐chemotherapy‐drugs‐work.html), but it also kills the latter, non‐target cells, especially those that also proliferate rapidly, with some probability. To increase selective toxicity, more discriminatory features of cancer cells have been actively sought, especially cancer‐specific cell surface antigens. This has enabled the development of agents targeting cells bearing such antigens, like ‘next‐generation therapeutic antibodies, including bispecific antibodies, nanobodies, antibody‐drug conjugates, glyco‐engineered antibodies, and antibody fragments’ (Raja, Kasana, and Verma 2024), and microbes and microbe components such as minicells and extracellular vesicles that target such cancer cell‐specific antigens through complementary surface‐expressed ligands (Salema and Fernandez 2017). Both antibodies and microbes/microbial components are often ‘armed’ with ‘payloads’ of cytotoxic agents that kill cells to which they bind (Li et al. 2024; Xi et al. 2024; Zhang et al. 2024).

More broadly, the microbiome influences the onset, progression and therapy outcomes of diverse cancers (Garrett 2015; Nejman et al. 2020). For example, a strain of *Staphylococcus epidermis* of the skin microbiome provides protection against skin neoplasia (Nakatsuji et al. 2018). The gut microbiota and the myriad of metabolites it produces a profound influence on the activities of immune pathways, signalling and inflammatory responses, all of which play roles in cancer initiation and progression. Gut microbiome dysbiosis seems to increase the risk of certain cancers. Moreover, and crucially, cancer tissues can have their own microbiota, which influence the tumour environment and can interfere with immune responses, metastasis and therapy efficacy (Nejman et al. 2020; Zhao et al. 2023). The recent appreciation of the role of the microbiome in cancer opens up new options for cancer diagnosis, prophylaxis and therapy interventions.

### 5.4 Neuropsychiatric and Behavioural Disorders

There is increasing evidence of associations, via the gut‐brain axis, between the gut microbiota and mood (An et al. 2024; Morris et al. 2017; https://longevity.stanford.edu/lifestyle/2024/04/08/more‐than‐a‐gut‐feeling‐how‐your‐microbiome‐affects‐your‐mood/), neuropsychiatric and behavioural disorders (Clapp et al. 2017; Carbia et al. 2023; Cryan et al. 2020; Delanote et al. 2024), such as stress and anxiety (Neuman et al. 2015; Butler et al. 2023), depression (Radjabzadeh et al. 2022; Xiong et al. 2023), schizophrenia, autism, Alzheimer's disease and Parkinsonism. Gut microbes produce neurotransmitters, such as serotonin, dopamine, norepinephrine, gamma‐aminobutyric acid and neuroactive short‐chain fatty acids that influence emotions and mood (https://longevity.stanford.edu/lifestyle/2024/04/08/more‐than‐a‐gut‐feeling‐how‐your‐microbiome‐affects‐your‐mood/). For example, microbes produce 95% of the serotonin of the body and much of the tryptophan needed for serotonin synthesis by the brain (Nunzi et al. 2024). This makes the gut microbiota an important target for interventions for mental health and neuropsychiatric disorders (Xiong et al. 2023; García‐Cabrerizo and Cryan 2024; Glinert et al. 2024; Schneider et al. 2024), including through the use of psychobiotics—microbes that alleviate neuropsychiatric disorders and prebiotics that stimulate proliferation of such microbes (Sarkar et al. 2016; Berding et al. 2023).

## 6 Microbial Technology Barriers to Malnutrition

### 6.1 The Scale of Malnutrition and Food Insecurity

Humans, like all forms of life, need food: a diet that is calibrated quantitatively and qualitatively to the chemical make‐up and energetic and ecophysiological needs of the organism. Undernourishment results in malnutrition, hunger and disease. About one third of the world's population is inadequately nourished and almost 10% face hunger (https://openknowledge.fao.org/server/api/core/bitstreams/06e0ef30‐24e0‐4c37‐887a‐8caf5a641616/content; https://www.who.int/news/item/06‐07‐2022‐un‐report‐‐global‐hunger‐numbers‐rose‐to‐as‐many‐as‐828‐million‐in‐2021#:~:text=Around%202.3%20billion%20people%20in,207%20million%20in%20two%20years; https://www.who.int/news/item/24‐07‐2024‐hunger‐numbers‐stubbornly‐high‐for‐three‐consecutive‐years‐as‐global‐crises‐deepen‐‐un‐report; https://ourworldindata.org/hunger‐and‐undernourishment), with the problem most acute in Africa where 20% face hunger. Global warming and regional conflicts are two important factors hindering improvements in food supplies. Poor nourishment and hunger are not only direct causes of suffering and premature loss of life, but also predisposing factors for disease in general (https://www.fao.org/4/y5650e/y5650e03.htm; https://www.who.int/news‐room/fact‐sheets/detail/malnutrition).

Food insecurity (https://www.fao.org/4/y4671e/y4671e06.htm; https://openknowledge.fao.org/server/api/core/bitstreams/06e0ef30‐24e0‐4c37‐887a‐8caf5a641616/content) can have different causes, including food cost‐poverty, inadequate availability of fertile farmland that can sustain local populations, poor global availability due to bad weather, warfare and warfare‐caused interruption of supply chains, crop and food animal pest and infection epidemics, loss of fertile soil and farmland due to global warming and extractive agricultural practices, extreme weather events and soils erosion, and overland fires (Timmis and Ramos 2021). While this diversity of causes of food insecurity requires a diversity of measures to improve food supply, microbial technologies currently play and will increasingly play key roles in the production of foods (Diaz‐Troya and Huertas 2024), in fermenting diverse raw plant and animal products into foods with improved nutritional characteristics, safety and shelf lives (Graham and Ledesma‐Amaro 2023; Bernal 2024), in increasing crop plant yields and plant resilience to increasing stresses, and in countering loss of soil fertility, fertile soils and desertification (Anand et al. 2023).

### 6.2 Current Food Production Systems Cannot Provide Long‐Term Food Security for the Global Population

Most food consumed by humans is of three types: products of crop plants, products of terrestrial animals reared on crop plant products and grasslands, and products of aquatic animals, both ‘farmed’ and wild. Terrestrial food animals are upgraders: they convert low‐grade food materials such as grass and hay into high grade, especially protein‐rich, foods. Crop plants require cultivation on fertile soils—a diminishing resource—and treatment with diverse agrochemicals that pollute the environment, reduce biodiversity, and the production of which is associated with high energy inputs and significant carbon footprints that promote global warming. There is competition for use of fertile soil for production *inter alia* of human food, food for food animals and companion animals (pets; Alexander et al 2020), and feedstocks for biofuels (Okin 2017; https://ourworldindata.org/environmental‐impacts‐of‐food; https://ourworldindata.org/global‐land‐for‐agriculture). Sources of aquatic food animals suffer from over‐fishing and reducing yields, in the case of wild animals, and epidemics and contamination with growth promoters, in the case of ‘farmed animals’. Contamination of water bodies with industrial pollutants, eutrophication and the formation/persistence of oxygen minimum zones, pollutant bioaccumulation in the food chain, and pathogen concentration in filter feeders, are all challenges for the sustainability of finfish and shellfish harvesting.

Quite apart from the ethical issues of food animal husbandry, terrestrial animal food production is associated with extremely low efficiency of conversion of plant food into animal food (i.e., significant wastage of crop plant resources—it has been estimated that only 5%–25% of input cellulose is converted into animal biomass: Javourez et al. 2024), pollution of the environment with animal wastes, high levels of non‐clinical use of growth promoters that also pollute the environment and, crucially, the production of greenhouse gases, especially by ruminants. Essential issues that rarely enter discussions about food security, but eloquently emphasised by Willy Verstraete and his colleagues (e.g., Javourez et al. 2024) are that *microbial* conversion of low‐grade food materials into high grade materials is 2–10× more efficient than *animal* conversion, much less polluting, is associated with much lower energy and chemical inputs with their resulting carbon footprints, and does not require fertile soils. Current food production systems neither assure global food security nor are sustainable; increased use of microbial food production technologies is essential for food security, sustainability and mitigation of global warming.

### 6.3 Balanced Diet‐Healthy Gut Microbiome

Since all or most organisms are metaorganisms/holobionts with an integral microbiome that provides diverse goods and services and significantly influences organismal well‐being, a healthy diet also reflects the needs of a healthy microbiome. However, since the gut microbiome itself contributes essential nutrients, host food needs are not identical to host food intake needs. Importantly, the interplay of host, gut microbiome and diet determines a range of gut functionalities, such as immune training (Rook 2022), the control of inflammatory processes and preservation of the gut epithelial barrier (tight junctions), that in turn influence the health of the host and its microbiome.

Maintaining a healthy gut microbiota that provides key functionalities necessitates nutrients that feed and favour beneficial microbes. Human breast milk is an example of a food that favours proliferation of beneficial microbes in the infant gut (Masi and Stewart 2024). Another is dietary fibre which also plays a key role in gut microbiota health (Ramsteijn and Louis 2024; https://longevity.stanford.edu/lifestyle/2024/04/08/more‐than‐a‐gut‐feeling‐how‐your‐microbiome‐affects‐your‐mood/), However intake of dietary fibre has been steadily decreasing as consumption of ultra‐processed, low fibre ‘fast foods’ has increased (Dicken and Batterham 2024), and is now below recommended levels in many countries (Ramsteijn and Louis 2024). Establishment and maintenance of a healthy gut microbiota and gut functionalities is especially important for developmental processes in infants in children (Fontaine et al. 2023; Mostafa et al. 2024); malnutrition in children has lifelong consequences (https://data.unicef.org/resources/jme‐report‐2023/; Blanton et al. 2016; Fontaine et al. 2023).

### 6.4 Special Diets‐Special Foods

Mediterranean diets, rich in vegetables, fruits, fish, grains, pulses and nuts, complemented with olive oil and modest amounts of wine, are associated with diverse gut microbiomes, beneficial microbes, healthy lives and longevity (Gerber and Hoffman 2015; Wang, Nguyen, et al. 2021).

Fermented foods also contain components that contribute to good health. Food fermentation has been employed for millennia for plant and animal products (Gänzle 2022). During fermentation, naturally occurring microbes or added microbial starter cultures transform carbohydrates, proteins and lipids to a variety of structurally different metabolites with various functionalities, generate distinctive flavours and aromas and improve food texture and appearance. Short‐chain carboxylic acids and other antimicrobial compounds are produced that inhibit the growth of microorganisms responsible for deterioration processes and hence raise barriers to food spoilage. Fermentation technology can thus be considered a biopreservation process reducing food spoilage and waste (Bernal 2024). Enzymatic activities of microbes also reduce anti‐nutrients that are naturally present in plant‐based raw materials. Major fermenting microorganisms are food‐grade *Lactobacillaceae*, *Propionibacteriaceae* and *Acetobacteraceae*. It has been estimated that 5%–40% of the human diet is fermented.

### 6.5 Unhealthy Foods‐Unhealthy Gut Microbiota

On the other hand, some foods are unhealthy. Ultra‐processed foods and food additives are associated with poorer health and especially an increase in type 2 diabetes and obesity (see Du et al. 2024, and references therein; Lane et al. 2024). Such foods, which are produced on an industrial scale, mostly as ‘hyper‐palatable, cheap, ready‐to‐consume’ fast foods (Monteiro et al. 2013), are increasingly used as lifestyles change and people choose or are obliged to repartition their daily schedules to reduce the times they allocate to food preparation and consumption (Baker et al. 2020; https://www.nature.com/articles/d42473‐024‐00020‐7.pdf). Importantly, ultra‐processed foods have a significant impact on gut microbiota composition and function, which is linked to increased intestinal permeability and inflammation, and inflammatory bowel disease, colorectal cancer and irritable bowel syndrome (Du et al. 2024; Whelan et al. 2024).

### 6.6 Microbial Technology Barriers Against Micronutrient Deficiencies

Micronutrients are organic or inorganic elements and compounds such as vitamins and minerals. They are crucial for the maintenance of human health: some are components of enzymes that carry out key cellular activities, others regulate biosynthetic cellular reactions essential for immune functions and energy generation, and yet others are essential for biological processes such as growth, bone health, and fluid balance (https://ourworldindata.org/micronutrient‐deficiency; https://www.fao.org/4/X0245E/x0245e01.htm; https://www.fao.org/4/X5244E/X5244e03.htm; https://www.bbc.com/future/bespoke/follow‐the‐food/the‐hidden‐hunger‐affecting‐billions/). Micronutrients also modulate the diversity and composition of the gut microbiome, leading to beneficial or detrimental outcomes for human health. Humans cannot synthesise all the required micronutrients, so they need to acquire them exogenously mainly from three major sources: (a) dietary components, (b) synthesis by commensal gut bacteria and (c) as oral food supplements.

One quarter of the world's population, and half of the children, may suffer from micronutrient deficiencies resulting from reduced intake and/or poor absorption that lead to or aggravate many chronic diseases, such as allergies, inflammatory diseases, metabolic and endocrine disorders, cardiovascular diseases and even cancer (Shenkin 2006). Micronutrient deficiencies dramatically impact the quality of life, lead to physical and mental dysfunctions, and increase susceptibility to infectious diseases by impairing immune functions (Barone et al. 2022; Noushin et al. 2021).

As mentioned above, microbes produce a range of nutrients in the gut that are essential for the host. In analogous fashion, microbes can produce in cell factories such nutrients to be used as food supplements.

Another promising strategy to reduce micronutrient deficiencies is biofortification: the increase in levels of micronutrients in crop plants. This may involve classical plant breeding, rhizosphere microbiota interventions or genetic engineering. Rhizosphere microbiota interventions are particularly interesting because existing crop plants can be used and applications can be rapid (Dhiman et al. 2023; Kumari et al. 2023). However, plant:microbe:soil interactions are dynamic and not always reproducible.

Another approach being explored relates to phytate, which is a major storage form of phosphorus in cereals, legumes, oil seeds and nuts (Gupta, Gangoliya, and Singh 2015). Although phytate may have some health benefits, it is considered an antinutritional factor because it also forms complexes with dietary minerals, especially iron and zinc, and causes mineral‐related micronutrient deficiency in those humans who are primarily dependent upon grain‐based foods. For this reason, phytase supplementation of human plant food is under investigation to find the right conditions that maximise benefits of both phytate and phytase (Kumar et al. 2010; Dersjant‐Li et al. 2015).

The gut microbiota regulates the intestinal levels of essential vitamins (such as vitamins B‐group, C, D, E, K, etc.), minerals (such as calcium, magnesium, iron and phosphorus), and health‐related compounds, such as short‐chain fatty acids (like acetic, propionic and butyric acids). Importantly, the gut microbiota also influences micronutrient uptake (Barone et al. 2022; Noushin et al. 2021; Lin and Medeiros 2023). Gaining an understanding of the intricate interplay of host, microbiota and nutrition in gut physiology, and the relevant biochemical pathways and their regulation, will undoubtedly help develop microbiome and nutrition interventions that increase levels and absorption of micronutrients and hence barriers against micronutrient deficiencies.

### 6.7 Soil Health and Human Health

Plants are the base of terrestrial food chains that lead to human nutrition and thus play a major role in nutrition‐related health. They are also pivotal for biodiversity and the services it provides, and for carbon capture (and, with microbial partners, burial) and hence global warming and its threats to health. Therefore, any parameter that generically influences plant health and productivity influences human health. Soil fertility is fundamental to plant productivity and crop yields, and soil availability determines the extent to which crop plants can be grown, hence the soil health:human health relationship. But soil health and food production is only one aspect of the importance of soil (https://www.weforum.org/agenda/2024/10/soil‐health‐human‐health‐connection/?utm_source=sfmc&utm_medium=email&utm_campaign=2837004_AgendaWeekly‐4October2024&utm_term=&emailType=Agenda%20Weekly). Besides supporting plant growth, soil functions as a complex ecosystem for animals and microbes, acting as a natural reactor that purifies water, replenishes aquifers and balances surface waters through various chemical and biological processes. It plays an essential role in biogenic cycles pivotal to life. About 20% of the carbon fixed by plants is released into the soil, enriching its organic matter, improving soil quality and vitalising it and the organisms that call it home (Ramos and Timmis 2021; Timmis and Ramos 2021; Singh et al. 2023; Ulbrich et al. 2022). Although sequestration of carbon in soils is crucial for mitigating climate change, poor soil management practices can exacerbate greenhouse gas emissions. Both the United Nations and European Commission stress the importance of sustainable soil practices to reduce these risks. In response to these vital functions, the European Union launched a mission program to enhance soil health and restore polluted sites (https://rea.ec.europa.eu/funding‐and‐grants/horizon‐europe‐cluster‐6‐food‐bioeconomy‐natural‐resources‐agriculture‐and‐environment/soil‐mission_en). This initiative highlights the importance of maintaining healthy soils, an issue as important as plastic or water pollution.

The growing global population, projected to reach 10 billion by 2050 (https://ourworldindata.org/un‐population‐2024‐revision), demands greater food production, underscoring the need to preserve and build healthy topsoil. Effective soil management is key to tackling global challenges like food insecurity, climate change and biodiversity loss (https://openknowledge.fao.org/server/api/core/bitstreams/a4fd8ac5‐4582‐4a66‐91b0‐55abf642a400/content). Soil biodiversity, which includes an immense reservoir of microscopic life, plays a critical role in these processes. For instance, a single gram of rhizosphere soil (the soil near plant roots) can contain between 10 and 100 million microbes, while bulk soil can hold 10,000 to 1 million microbes per gram. Despite this diversity, < 1% of soil microbes have been successfully cultivated in laboratories and characterised (Lutz et al. 2023; Panda and Zhou 2023). Recent advances in metagenomics are revealing the extent of this microbial diversity and their interactions, which are essential for sustaining biogenic cycles and restore natural ecosystems (Ramos, de Lorenzo, and López 2024; Singh et al. 2023).

To address the importance of soil health we should take into account that nearly a century is needed to form just 3 mm of topsoil, making it a non‐renewable resource within human lifespans. It is estimated that 33% of the world's land is threatened by desertification, with 25% of European agricultural soils severely damaged. Climate change compounds these issues by reducing rainfall, increasing desertification and threatening agricultural output. Projections suggest a 30% decline in food production due to plant diseases and plagues, potentially leading to hunger, mass migrations, and economic and socio‐political instability (https://www.undrr.org/understanding‐disaster‐risk/terminology/hips/en0019; https://www.weforum.org/agenda/2024/09/triple‐cop‐year‐leaders‐align‐efforts‐planetary‐health/?utm_source=sfmc&utm_medium=email&utm_campaign=2836567_AgendaWeekly‐27September2024&utm_term=&emailType=Agenda%20Weekly).

Soils also vary in terms of their plant pathogen burdens, which can determine the health and yield of a crop. Repeated cultivation of the same crop in the same soil can enrich for pathogens of that crop and a progressive lowering of yields. On the other hand, some soils—*disease‐suppressive soils*—can either prevent establishment of relevant pathogens, or allow their establishment but restrict their potential to cause disease (Baker and Cook 1974). Disease‐suppressive soils suppress disease because of microbial activities (e.g., Mendes et al. 2011), characterised by a competitive environment of a high and active microbial biomass, which limits access of the pathogen to available resources, and by production of antimicrobial compounds (e.g., see Schlatter et al. 2017). Importantly, specific suppressiveness can be transferred to and conferred upon disease conducive soils, thereby increasing plant health and yields.

Protecting soil is important for rural economies and enhances agricultural sustainability. Initiatives like the European Green Deal promote soil health policies aimed at achieving sustainable agriculture and forestry by 2030 and 2050 (https://rea.ec.europa.eu/funding‐and‐grants/eu‐mission‐soil‐deal‐europe_en). Healthy soils help mitigate desertification, pollution and biodiversity loss, while also supporting microbial systems that enable plants to tolerate extreme conditions. Microbial technologies can help improve marginal soils, offering a solution to expand cultivable land and boost food security in vulnerable regions (Maestre, Sole, and Singh 2017; Bernal 2024).

For these reasons, a more proactive approach to preserving soil fertility, slowing the process of desertification, regenerating low‐fertility soils and maintaining our ability to produce food for a growing global population has been proposed (Timmis and Ramos 2021; see also Rhodes 2017). This urges adoption of policies that treat soils worldwide as ‘patients in need of healthcare and creation of (a) a public health system for development of effective policies for land use, conservation, restoration, recommendations of prophylactic measures, monitoring and identification of problems (epidemiology), organizing crisis responses, etc., and (b) a healthcare system charged with soil care: the promotion of good practices, implementation of prophylaxis measures, and institution of therapies for treatment of unhealthy soils and restoration of drylands’ (see also Maestre, Sole, and Singh 2017). It further recommends ‘elaboration of internationally agreed laws to protect the environment, to define ecocrimes/ecocide/environmental crimes, including those that deliberately degrade soil health or pollute, and appropriate sanctions, and creation of the International Environmental Court (https://www.ibanet.org/Article/NewDetail.aspx?ArticleUid=71b817c7‐8026‐48de‐8744‐50d227954e04; Greene 2019; Solntsev 2019) to prosecute/adjudicate such laws’.

## 7 Microbial Technology Barriers to Intoxication

### 7.1 The Disease Burden of Intoxication

Intoxication—the poisoning of the body—takes many forms with many outcomes, ranging from mild discomfort to death, and from acute to chronic disease. Particularly concerning is long‐term (chronic), low‐level exposure to environmental chemicals (micropollutants), including diverse agrochemicals used on crop plants, with slowly developing symptoms that are recognised late in disease progression, and emerging pollutants (Wang, Xiang, et al. 2024; Wang, Li, et al. 2024) and contaminants of emerging concern (CECs; https://planbleu.org/wp‐content/uploads/2021/05/Pollution‐emergentes‐EN.pdf). An extension of intoxication is the exposure to allergens, which can also have a range of health outcomes.

According to Fuller et al. (2022), 9 million premature deaths were attributable to environmental pollution in 2019, with air pollution responsible for 6.7 million and water pollution for 1.4 million. But the authors remark that these are underestimates because of the lack of comprehensive health data. The WHO estimates that 24% of global deaths are linked to the environment (https://www.who.int/data/gho/data/themes/topics/sdg‐target‐3_9‐mortality‐from‐environmental‐pollution).

### 7.2 Chemical Toxins

Chemical toxins are highly diverse and have many sources, such as industrial manufacturing processes, mining, including the extraction, processing and use of fossil fuels, accidents of transportation and storage of chemicals, handling and disposal of wastes, agricultural practices, domestic use of fossil fuels, and mobility. Many pollutants are channelled into municipal wastewater treatment plants, whereas industrial pollutants are usually treated on‐site at the point of production. In both cases, the treatment processes may or may not remove all of the pollutants and, if not, they enter the environment where they may negatively impact the health of life forms in the biosphere, including that of humans. Non‐degradable pollutants and heavy metals like mercury that reach the sea can bioaccumulate in the food web and reach dangerous levels in top predators, like tuna, which may become part of our diet (e.g., see https://www.bloomassociation.org/en/mercury‐contamination‐bloom‐exposes‐a‐health‐scandal‐on‐an‐unprecedented‐scale/). Delayed cognitive development has been observed in children suffering from mercury intake resulting from fish‐rich diets (Freire et al. 2010).

Whereas the primary action needed to reduce chemical intoxication is the reduction in production and release of pollutants, this is difficult to achieve in many cases and will take considerable time in others. In addition to ongoing production of pollutants and their contamination of air, water and soils, there are many ‘legacy sites’, sometimes containing massive amounts of toxic chemicals that can leak into and disperse in surrounding soils, watersheds and, through volatilisation, into above‐ground air.

Microbes are the great transformers—the catalysts of biogeochemical processes—and are key agents of processes that transform many toxic pollutants into harmless or less harmful products. Microbes are the basis of wastewater treatment and bioremediation technologies that reduce pollutant levels and hence human exposure to and suffering from chemical intoxication (e.g., Timmis, Steffan, and Unterman 1994; Young and Cerniglia 1995; Durante‐Rodríguez et al. 2024). Even emerging pollutants and non‐degradable metal pollutants, such as the example of mercury above, can be transformed by microbes from toxic species into less toxic or less bioavailable species, activities that have been developed into bioremediation technologies (Wagner‐Döbler et al. 2000; Ding et al. 2024; Kariyawasam et al. 2024). Microbial technologies for pollutant mitigation raise barriers to morbidity and mortality and improve quality of life. However, some communities still lack these basic technologies so there is an urgent need to deploy them where needed.

### 7.3 Air Pollution

According to the WHO, ‘Almost all of the global population (99%) are exposed to air pollution levels that exceed the safe WHO guideline level…’ (https://www.who.int/teams/environment‐climate‐change‐and‐health/air‐quality‐energy‐and‐health/health‐impacts/exposure‐air‐pollution#:~:text=Ambient%20(Outdoor)%20Air%20Pollution%20Exposure,the%20highest%20levels%20of%20exposure). Exposure in utero can have lifelong health impacts (https://www.stateofglobalair.org/sites/default/files/documents/2024‐06/how_does_air_pollution_impact_childrens_health_factsheet.pdf). Further: ‘An estimated 4.2 million deaths globally are linked to ambient air pollution, mainly from heart disease, stroke, chronic obstructive pulmonary disease, lung cancer and acute respiratory infections. The health consequences of air pollution have a significant economic impact’ (https://www.oecd‐ilibrary.org/docserver/56119490‐en.pdf?expires=1725607601&id=id&accname=guest&checksum=52AFE630E9AF166C7CD33F792651BFA3). Air pollution is often coupled with odour pollution, because the responsible gases‐volatile organic chemicals (VOCs) are often malodorous, which also lowers the quality of life in affected localities (Rotton 1983; Shusterman 1999; Guadalupe‐Fernandez et al. 2021).

Global warming has diverse impacts on air pollution, including levels of ozone, particulates, organic aerosols and persistent organic pollutants, which in turn influence the composition of the aerobiome, the air microbiota (Robinson et al. 2024), all of which are taken into the lungs in the average 6 L of air that humans inhale per minute (Pleil et al. 2021).

While air pollution comes in many forms and from many different types of sources, industrial off‐ and process‐gases are an important source and many can be captured and rendered harmless by microbial filters (Devinny, Deshusses, and Webster 1998; Komang Ralebitso‐Senior et al. 2012; Lan et al. 2020) in processes called biofiltration, biotrickling filtration and bioscrubbing, in some instances also generating electrical energy in the process (Liu, Lin, and Lin 2023). At‐source treatment of industrial off gases by microbial systems not only removes polluting gases, lowering emissions and thereby reducing health impacts, but also captures carbon and reduces carbon emissions, thereby reducing their contribution to global warming and its health impacts.

### 7.4 Non‐infectious Food Poisoning

Food can be contaminated in various ways by a variety of toxins. A major class is microbial toxins that can be introduced at different stages in the food supply chain. These include mycotoxins (https://www.who.int/news‐room/fact‐sheets/detail/mycotoxins#:~:text=Key%20facts,under%20warm%20and%20humid%20conditions) produced *in planta* or during post‐harvest fungal growth, like aflatoxins, toxins found in shellfish and finfish harvested from waters experiencing harmful algal blooms, and toxin‐containing meals, the preparation of which allowed growth of toxin‐producing microbes, like *Staphylococcus aureus* , *Clostridium* spp., *Bacillus cereus* and so forth in a component of the meal. Unlike most food‐borne pathogens which can be killed by cooking, many toxins are heat stable, and are not inactivated by cooking. Allergens can also be delivered by food, either as inherent components, or extrinsic substances acquired along the farm‐to‐fork chain.

While much of the strategy to avoid food poisoning revolves around hygiene practices, an important microbiological technology is that of toxin and allergen detection/monitoring which enables the removal of contaminated materials from the food supply chain and identification and elimination of operational practices that allow toxin contamination of food. While there are a number of different ways of detecting and measuring toxins and allergens in food, biosensors and lateral flow tests have the advantages of portability—they can be used essentially anywhere, so on‐site at any point in the food supply chain, are usually simple to use, so do not need highly trained personnel, and many are or can be made frugal, so can be used worldwide. Many such tests are based on immunological detection of the target substance and use antibodies and control antigens produced in microbial cell factories.

### 7.5 Agrochemical Intoxicants and Causes of Intoxication

Pollution with agrochemicals is particularly insidious because they can enter the human food supply chain (Ahmad et al. 2024) but also directly affect people handling them or the products they have been used to treat (e.g., see: https://www.bbc.com/news/articles/c4glydv8qlgo). Moreover, chemical pesticides not only directly affect humans but, because of their low specificity, poison a range of non‐target organisms. These include beneficial insects, such as pollinators (Stuligross and Williams 2021; Douglas et al. 2022), and earthworm ecosystem engineers (Zeng et al. 2024, both of which are essential for food security which is a barrier to malnutrition. Contaminated worms are in turn eaten by birds (see also Carson 1962). Non‐target effects of pesticides are exacerbated by climate change (Sirois‐Delisle and Kerr 2022).

Agrochemical fertilisers, primarily nitrogen (see Verstraete 2024, for a comprehensive overview of nitrogen and its benefits and hazards) and phosphorus compounds, while not toxicants in of themselves, engender toxin production by causing eutrophication and harmful algal blooms in receiving water bodies. This is due to the fact that agrochemical fertilisers are highly mobile in water and, rather than being used by target plants for growth, mostly migrate in run‐off into receiving water bodies. Microalgae that inhabit these water bodies are mostly nitrogen‐ and/or phosphorus‐limited for growth. Their population sizes determine the nature of prevailing aquatic food webs. An influx of agricultural fertiliser removes the growth limitation and there is a burst of growth—a bloom—of microalgae, the process of eutrophication, that exceeds the grazing capacity of available predators and hence perturbs the food web. Growth of the microalgae therefore continues until nutrients are exhausted, after which they die and are degraded by bacteria which in the process consume most or all of the available oxyge.

The microalgae and cyanobacteria that bloom in response to the inflow of nutrients often produce toxins, particularly neurotoxins, many of which are lethal to aquatic animals, especially filter feeders and animals feeding on them, fish, larger aquatic species and birds feeding on fish, and humans (https://unesdoc.unesco.org/ark:/48223/pf0000233419; https://www.fisheries.noaa.gov/feature‐story/toxic‐algal‐bloom‐suspected‐dolphin‐and‐sea‐lion‐deaths‐southern‐california; Karlson et al. 2021). Algal blooms mandate the prohibition of harvesting, sale and consumption of affected shellfish and finfish, so reduce food security and have significant economic impacts, especially on coastal populations dependent on fish. Eutrophication also creates oxygen minimum (dead) zones in aquatic systems, causing the death of oxygen‐requiring species, loss of coral reef systems and local biodiversity (Altieri et al. 2017), and frequently, major kills of fish that again decrease food security.

Although harmful algal blooms are also naturally caused by the transportation of nutrients from nutrient‐rich to nutrient‐poor environments, for example by upwelling events involving nutrient‐laden deep cold waters (Pitcher et al. 2010), and wind transportation/deposition events of nutrient‐rich soil particles to nutrient‐poor photic surface waters (https://www.epa.gov/habs/climate‐change‐and‐freshwater‐harmful‐algal‐blooms#:~:text=Coastal%20upwelling&text=Along%20the%20west%20coast%20of,nutrient%20pollution%20from%20the%20land), a major contributor to eutrophication is agrochemical fertiliser run‐off from agricultural land. The only means of countering this source of eutrophication is massive reduction in the use of *agrochemical* fertilisers on farmland. But, because of the need for fertiliser to achieve crop yields needed to feed the growing human population, in order to do this, alternative fertilisers are required: the *agrobiologicals* (Bernal 2024).

Plant symbiotic nitrogen‐fixing bacteria (rhizobia) live in symbiosis with leguminous plants, fix atmospheric nitrogen and make it available to the plant. N‐fixation by bacteria associated with non‐leguminous plants also exist. Other microbes, particularly mycorrhizal fungi, solubilise and make available phosphorus in soil that is otherwise unavailable to plants. Plant‐associated microbes also mobilise micronutrients important for plant growth and vitality. *Agrobiologicals* not only include microbes that provide or facilitate acquisition of plant nutrients and plant stress tolerance promoters, but also serve as biopesticides against a variety of plant pathogens and pests (bacterial, fungal, insect, nematode: Ruffner et al. 2012; Collinge et al. 2022; Bernal 2024; Compant et al. 2025; Erdrich et al. 2024; Jiménez et al. 2024). Importantly, in contrast to *agrochemica*l pesticides, *agrobiologicals* tend to be highly specific, not affecting non‐target organisms like pollinators, and have a low impact on ecosystems and the services they provide.

### 7.6 Animal Venoms and Toxins

Many animals, including bees and wasps, ants, spiders, jellyfish and snakes, produce toxins (Chen et al. 2018). Snake envenoming—the injection of venom and resulting disease (Gutiérrez et al. 2017)—has been called the world's biggest hidden health crisis (Basnyat and Shilpakar 2022), with more than 5 million people bitten by snakes each year, resulting in ca. 2 m envenomings, ca. 100,000 deaths, and ca. 300,000 amputations and permanent disabilities (Kasturiratne et al. 2008; https://www.who.int/news‐room/fact‐sheets/detail/snakebite‐envenoming#:~:text=An%20estimated%205.4%20million%20people,are%20caused%20by%20snakebites%20annually; https://www.nature.com/articles/s41467‐022‐33627‐9#citeas). Snake bites are particularly important in rural areas of tropical and sub‐tropical countries, and children are particularly vulnerable because of their smaller body mass. Anti‐venom antibodies are generally effective treatments, but their timely delivery to patients can be challenging in settings with weak health systems and infrastructure. Moreover, even under ideal clinical treatment conditions, anti‐venoms are not perfect (Hamza et al. 2021; Benard‐Valle et al. 2024). However, there is considerable current activity developing new, more effective anti‐venom therapeutics based on oligoclonal nanobodies obtained by phage display technology, and produced in microbial cell factories (see Ledsgaard et al. 2023; Benard‐Valle et al. 2024, and references therein).

### 7.7 Ecosystem Engineering Microbial Barriers for Health and Resilience

The issue of environmental pollution caused by chemical emissions from urban, agricultural and industrial activities is a well‐documented, critical challenge for the future of our planet. It has long been understood that many microorganisms can degrade pollutants into CO_2_ and water. The study of interactions between microorganisms and chemical pollution gained significant momentum with the advent of recombinant DNA technology in the mid/late 1980s. On one hand, molecular techniques allowed microbiologists and biochemists to explore and dissect the immense catalytic potential of environmental microorganisms in combating contaminants. On the other, these technologies anticipated the potential to genetically engineer new traits, creating super‐catalysts that, once released, could address many environmental issues caused by different types of emissions (Timmis, Steffan, and Unterman 1994). However, these early efforts were hampered by the limited understanding of microbial ecology at the time and opposition from environmental groups to genetic engineering. As a result, while the promise of these approaches was recognised, their immense potential remained largely unrealised (Cases and de Lorenzo 2005).

A few decades later, we are now facing a vastly different pollution scenario, compelling us to revisit some of the earlier ambitions and integrate them with modern tools that were unavailable back then. While the traditional response to the widespread presence of diverse chemicals and greenhouse gases has focused on prevention and monitoring, the time is now ripe for entertaining large‐scale bioremediation interventions that not only mitigate but reverse environmental damage (de Lorenzo, Marlière, and Solé 2016). In this way, we can now consider actions aimed at counteracting pollution in both targeted and extensive ecosystems. The objective in these cases is not only to rehabilitate them, but also protect them from future aggressions and secure their long‐term functionality (Hassard et al. 2024). Note that successful large‐scale ecosystem engineering for the sake of sustainability is not a new endeavour, as evidenced by the effort along the XIX century to bring a balanced biological network to Ascension Island (Wilkinson 2004).

How can this be achieved in our case? First, we now possess a far greater understanding of microbial ecology and element cycling on a planetary scale. The catalytic potential of the global environmental microbiome (Paoli et al. 2022) may be the most powerful actor in changing the state of the planet, as has been the case in other moments of our planet's History. The spectacular increase in Earth‐wide metagenomic data attests to this. Second, the nature and distribution of pollutants have also evolved. Whereas past bioremediation efforts concentrated on localised oil spills or toxic chemicals at specific sites, the current challenge lies not only in managing globally spread greenhouse gases but also in addressing ubiquitous micropollutants and microplastics (de Lorenzo 2017). A new branch of microbial biotechnology, which we are calling *Environmental Galenics* (de Lorenzo 2022), must be developed to deliver live catalytic agents on a large scale, capable of tackling these extraordinary challenges. There is also a third crucial aspect: the need to adopt advanced genetic engineering and synthetic biology to enhance the biochemical capabilities of microbial agents. While it is true that many environmental problems could be alleviated by simply reducing or stopping the sources of emissions, if stressed environments surpass tipping points, eliminating the causes alone will not be sufficient. In such cases, advanced interventions will be necessary, likely involving the release of catalysts genetically programmed to restore environmental balance.

This, of course, demands a broader public debate and the expansion of societal consensus around the technology, as the regulations governing these developments have remained stagnant for the last 30 years, despite remarkable advances in genome editing, environmental impact modelling (Conde‐Pueyo et al. 2020), and the development of entirely new enzymatic functions in the Laboratory (Arnold 2015; Hossack, Hardy, and Green 2023). Just as serious human diseases require advanced medical treatments, serious environmental problems require cutting‐edge solutions based on the best systems and synthetic (micro) biology of our time (Timmis and Ramos 2021).

## 8 Microbial Technologies to Confront Global Warming

### 8.1 The Health Burden of Global Warming

Global warming is likely to be the most important cause of increasing suffering and mortality in the coming years. Global warming‐induced climate change and extreme weather events affect well‐being, morbidity, mental health and mortality in a variety of ways (Gasparrini et al. 2015; https://iris.who.int/bitstream/handle/10665/107552/9789289010948‐eng.pdf?sequence=1&isAllowed=y; https://www.ipcc.ch/report/ar6/syr/), through a diverse range of often interacting and synergising causes (https://www.ipcc.ch/report/ar6/syr/downloads/report/IPCC_AR6_SYR_SPM.pdf), with the elderly and those suffering from underlying morbidities affected the most by weather extremes, especially women (Calleja‐Agius, England, and Calleja 2021; https://www.swissinfo.ch/eng/climate‐change/why‐older‐women‐are‐hit‐hardest‐by‐deadly‐heatwaves/78907902#:~:text=Older%20adults%2C%20especially%20over%2075,verdict%20against%20Switzerland%20in%20April; https://www.swissinfo.ch/eng/science/historic‐verdict‐could‐link‐climate‐crisis‐and‐human‐rights/75321434).

One study estimates that by 2050 global warming will have been responsible for 2 billion DALYs and 14.5 million premature deaths (https://www3.weforum.org/docs/WEF_Quantifying_the_Impact_of_Climate_Change_on_Human_Health_2024.pdf). Around 3.5 billion people live in regions highly vulnerable to climate change (https://www.ipcc.ch/report/ar6/syr/downloads/report/IPCC_AR6_SYR_SPM.pdf). Children, the elderly and people in low‐resource settings will be disproportionately affected and existing health inequities exacerbated (https://www.weforum.org/agenda/2024/09/children‐climate‐change‐impact‐health/?utm_source=sfmc&utm_medium=email&utm_campaign=2837004_AgendaWeekly‐4October2024&utm_term=&emailType=Agenda%20Weekly; Calleja‐Agius et al. 2021; https://www.who.int/news‐room/fact‐sheets/detail/climate‐change‐and‐health; see also Bressler 2021). The global economic cost, and health cost in particular, will be huge.

The effects of climate change on human health will become increasingly severe as the pace of climate change accelerates. Some existing health threats will increase and new health threats will emerge. There are strong indications that climate change may lead, *inter alia*, to an increase in respiratory and cardiovascular diseases, injuries and premature deaths related to extreme weather events, and changes in the prevalence and geographical distribution of food and waterborne diseases and other infectious diseases. This will inevitably lead to increases in hospitalisations, particularly for those with underlying illnesses, and rises in hospital‐acquired infections with pathogens that are often antibiotic resistant.

Weather extremes also cause desertification and erosion of fertile agricultural land, which reduces available acreage for crops and hence food supply, and thereby increases malnutrition and resulting disease. Global warming also provokes changes in the geographical distribution of organisms of the biosphere. One of many consequences is the killing of essential algal symbionts of coral, leading to the death of coral reefs and destruction of habitats of marine life and food webs that ultimately provide food for humans. Shoreline communities that are heavily dependent on fish for their nutrition are particularly affected and malnutrition‐related diseases alluded to above can result. Another consequence is the ability of insect disease vectors to populate regions at higher latitudes and introduce new pathogens into naïve populations. If (when) the human species goes extinct, global warming will probably be the cause. Humans thus have huge personal and collective self‐interests in mitigating greenhouse gas (GHG) emissions that drive global warming.

Microbes are intimately and essentially involved in a number of aspects of the production and consumption of GHGs (Cavicchioli et al. 2019) and these activities can be influenced in a number of ways. There are fundamentally two actions to reduce atmospheric GHGs: (a) reduce/prevent their production, and (b) capture them after production. There are a number of microbial technologies for reducing GHG emissions and capturing carbon, both of which can positively modulate carbon fluxes.

### 8.2 Carbon Capture and Carbon Burial

Photosynthesis is the basis of creation of most biomass, and hence food for most organisms of the biosphere. It involves the conversion of carbon dioxide, the principal GHG, to organic carbon, so photosynthetic organisms play a central role in carbon capture. For this reason, planting trees is often promoted as a key carbon capture strategy. However, most trees have a particular average lifetime, after which they die and their carbon is recycled by microbes, much of it back to CO_2_, mostly by white rot fungi (the emergence of which may have ended the formation of fossil carbon deposits typical of the Palaeozoic era; Floudas et al. 2012), if they are not consumed by forest fires beforehand. Carbon capture is thus distinct from carbon burial, which is the sink that removes carbon from the carbon cycle and has a long‐term positive impact on global warming. However, some of the carbon captured by photosynthesis is not used for tree growth but is transferred to the roots and released into the soil as exudate, which feeds the tree root microbiota. Tree root exudate‐promoted multiplication of rhizosphere microbes creates soil microbial biomass, some of which becomes soil organic matter (SOM), part of which is not recycled and becomes buried (e.g., see Pett‐Ridge et al. 2021, and references therein). Thus: planting trees is an effective means of capturing carbon from the atmosphere, but it is the tree–microbe partnership that is actually responsible for carbon burial. There are opportunities to improve the fraction of carbon captured that becomes carbon buried through microbial technologies for tuning soil and rhizosphere microbiomes.

That said, the primary problem of carbon capture by trees is deforestation: the destruction of existing stands of mature trees. Agrifood systems are the single biggest driver of deforestation, biodiversity loss and water use, accounting for one third of global greenhouse gas emissions. Vast tracts of land are cleared annually on an industrial scale to satisfy the ever‐growing demand for commodities from international markets (Sylvester et al. 2024).

In this context, therefore, it is important to note that photosynthetic microbes can carry out both carbon capture and burial on their own, both on soils, in aquatic systems, and importantly in constructed bioreactors that occupy very little space. Unlike trees, they do not need to take up any agricultural land, so do not compete with food production activities; indeed, some may actually contribute food. There are exciting developments to optimise and scale up such activities (Onyeaka and Ekwebelem 2023; https://www.bbc.com/future/article/20230829‐the‐bacteria‐that‐can‐capture‐carbon). Engineered microorganisms can also efficiently transform captured carbon into value‐added products (Bae et al. 2022; Turlin et al. 2022), including and importantly food (Sakarika, Ganigué, and Rabaey 2022).

### 8.3 Reducing Greenhouse Gas Emissions and Carbon Footprints of Industrial Activities

Greenhouse gas emissions that drive global warming, climate change and all of the health consequences that result from this, are still rising (https://ourworldindata.org/emissions‐by‐sector; https://www.c2es.org/content/international‐emissions/). There is an urgent need to massively reduce emissions and, where possible, to draw down some of the already released GHG. In recent years there has been an explosion in microbiology research and metabolic engineering aimed at reducing greenhouse gas (GHG) emissions, with notable successes (Wang, Nguyen, et al. 2021; Wang, Harindintwali, et al. 2021; Ahn et al. 2023; The Role of Microbes in Mediating Methane Emissions [Internet]. Washington, DC: American Society for Microbiology; 2023 Nov 15. PMID: 38194471; Wood et al. 2023; Hiis et al. 2024; Tiwari et al. 2024). These include the prevention of emissions at source by promotion of green practices, carbon‐negative manufacturing, renewable energy production such as microbial production of hydrogen fuel, hydrocarbon biofuels, conversion of GHGs to biofuels, chemicals and materials. Electromicrobial processes show considerable promise for conversion of organic wastes and methane into electricity. Carbon capture technologies involving photosynthetic microbes are important, as are off‐gas biofilters that capture industrially produced GHGs. Methylotrophs are important in microbial technologies to capture methane at source and convert it into useful products. The construction industry has a very significant carbon footprint, both in terms of GHG emissions and energy used. The incorporation of biological processes and biological products into building materials including bioconcrete and biobricks (https://www.weforum.org/stories/2020/02/researchers‐have‐invented‐a‐brick‐that‐can‐build‐itself/; Smirnova et al. 2023; Zuiderveen et al. 2023) can significantly lower this footprint.

Whereas these approaches can be highly effective in addressing point sources of carbon emissions or point origins of carbon capture, many important sources, such as sediments underlying water bodies, and sinks or potential sinks, are non‐point or diffuse, which is considerably more challenging. Nevertheless, the urgency of the global warming crisis demands attention to non‐point sources and sinks: large‐scale environmental microbial carbon capture technologies and technologies to stimulate natural carbon capture processes. For example, one major element of increasing GHG emissions is the global warming‐driven thawing of permafrost and frozen peatlands, with resulting metabolic activation of their microbial communities and production of methane. Fortunately, methane oxidising microbes that use methane as a growth substrate act as a methane biofilter (also in other settings like anaerobic sediments, gas clathrates and landfills) significantly reduce emissions (Dang et al. 2022; Venetz et al. 2024).

Nitrous oxide (N₂O) is a potent greenhouse gas, with a global warming potential about 274 times greater than carbon dioxide (CO₂) over a 100‐year period. N₂O emissions are particularly concerning in agriculture, wastewater treatment and industrial processes, where microbial activities significantly contribute to its release. Recent advancements in microbial technologies offer solutions to reduce N₂O emissions in nitrogen‐rich environments, such as wastewater treatment. One example is the anammox (anaerobic ammonium oxidation) process, which treats nitrogen without producing N₂O and provides a more energy‐efficient alternative to traditional nitrification and denitrification. Anammox bacteria convert ammonia directly into nitrogen gas under anaerobic conditions, minimising N₂O emissions (Henze et al. 2008). Another is the use of N₂O‐respiring bacteria (Hiis et al. 2024)

### 8.4 Reducing Dependence on Fossil Fuels

Biofuels are considered as a key solution to reduce GHG emissions, which peaked in 2023 with CO_2_ levels at 424 ppm and global temperature increase of 1.45°C over pre‐industrial levels (https://www.ipcc.ch/report/ar6/syr/summary‐for‐policymakers/; Fletcher et al. 2024). Terrestrial transport and air travel contribute significantly to emissions, with air travel contributing 785 million metric tons of CO_2_ in 2019 (https://theicct.org/wp‐content/uploads/2021/06/CO2‐commercial‐aviation‐oct2020.pdf) equivalent to the pollution from 164 million cars. Despite global agreements like the Kyoto and Paris protocols pushing green energies (https://unfccc.int/sites/default/files/unfccc_spm_2016.pdf), efforts to curb emissions have failed and the UN Climate Conference COP28 affirmed that anthropogenic pollution has an impact on global changes including severe droughts, wildfires and floods all over the planet (https://unfccc.int/sites/default/files/resource/Summary_GCA_COP28.pdf).

Current energy trends favour renewable sources such as solar, wind and biofuels. Countries like USA, Brazil, China and European Union have implemented biofuel programs to reduce emissions and to enhance energy security. However, biofuels currently account for a low percentage of global energy use.

Ethanol, primarily produced from corn, is the most common biofuel. The US produces about 15 million gallons annually, replacing about 500 million barrels of petroleum. Brazil produces 7.5 billion gallons from sugarcane. However, this bioethanol, known as first‐generation (1G) biofuel, is derived from food crops and raises concerns over food security, biodiversity and its impact on gasoline demand is limited.

Second generation (2G) biofuels and biochemicals, made from non‐edible sources like agricultural waste and municipal solid waste (MSW), offer a more sustainable alternative. It has been estimated that only the United States could produce 7.5 billion gallons bioethanol form MSW, which could replace up to 16% of the US transportation fuel, significantly reducing GHG emissions (Kalago et al. 2007).

Emerging biofuels like butanol, which blends better with gasoline, and advanced fuels like farnesane for aviation, are gaining attention. Additionally, microbial processes for producing chemicals and fuels, such as using methane and methanol for plastic production, offer interesting alternatives. Challenges remain, such as high cost of 2G biofuels and scaling up processes (Valdivia et al. 2020), but biofuels hold significant potential to mitigate climate change.

## 9 Microbial Technology Barriers to Wastage

### 9.1 Wastage of Planetary Resources

Geographical and special situation exceptions notwithstanding (e.g., regions of high rainfall that do not experience water shortages), wastage of essential resources in limited supply ultimately negatively impacts health and well‐being. For example, despite widespread hunger in the world, between 20% and 30% of all food produced is wasted: the equivalent of 1 billion meals per day in a world in which 735 million people go hungry (https://openknowledge.fao.org/server/api/core/bitstreams/10388b16‐5f1a‐45d0‐b690‐e89bb78d33bb/content, https://www.wfp.org/stories/5‐facts‐about‐food‐waste‐and‐hunger; https://www.un.org/en/observances/end‐food‐waste‐day; Javourez et al. 2024).

More broadly, the linear economy of linear production—resource in, product out, waste discarded (‘take‐make‐waste’; see also, e.g., Conway 2023)—and linear consumption—product used, product discarded—creates unnecessary waste that needs to be treated, in the process consuming various additional resources including energy and land (e.g., land available in Melbourne for landfill may run out in 2025, and wastes are increasingly being transported to landfill sites ever more remote, with the associated carbon footprint of the additional transportation needed: https://acehub.org.au/news/what‐is‐the‐linear‐economy‐and‐why‐do‐we‐need‐to‐go‐circular). Furthermore, waste creates toxic waste streams and greenhouse gases. Crucially, it often contains still useable limiting natural resources, such as clean water, used in production (e.g., clean water used in semiconductor manufacture: https://www.weforum.org/agenda/2024/07/the‐water‐challenge‐for‐semiconductor‐manufacturing‐and‐big‐tech‐what‐needs‐to‐be‐done/).

Consumerism, fuelled by policies of perpetual economic growth, advertising, media and peer pressure (‘new trends’), etc., and price competition, which often encourages environmentally damaging processes, promotes the linear economy, wastage of natural resources and pollution of the environment. This is exemplified by the fashion industry with fashions changing each year, much non‐recyclable/degradable clothing being made cheaply from materials produced from fossil fuels and being discarded after a short time, some ending up in the Atacama Desert to sit for hundreds of years (https://www.nationalgeographic.com/environment/article/chile‐fashion‐pollution), if it does not catch fire/is not burned first (https://www.wired.com/story/fashion‐disposal‐environment/).

Consumerism may be considered to be the deliberate creation of stranded assets (Caldecott et al. 2021). According to the United Nations, if the human population reaches 9.7 billion by 2050, the resources needed to support it will be three times that which planet Earth can provide (https://www.un.org/sustainabledevelopment/sustainable‐consumption‐production/). Both the linear economy and the policy of consumerism are unsustainable. They also negatively impact the biosphere in general, causing loss of biodiversity and reducing ecosystem resilience (according to the European Commission, 90% of biodiversity loss is caused by resource extraction and processing; https://environment.ec.europa.eu/topics/circular‐economy_en). This unnecessarily costs lives and causes suffering in various ways, such as polluting local soils, waters and aquifers, creating shortages of vital resources like clean water, driving up the cost of resources making them unavailable to the less affluent members of society, etc. (https://www3.weforum.org/docs/WEF_New_Nature_Economy_Report_2020.pdf).

The barrier to unnecessary human suffering caused by the linear economy is the circular economy, which is based on the fact that used products either continue to harbour some or all of the resources used in production, or new resources that can be exploited (https://www.un.org/sites/un2.un.org/files/circular_economy_14_march.pdf). The circular economy seeks to eliminate or at least reduce to a minimum resource wastage, by reusing and recycling production waste streams and used products. Where possible, the circular economy also seeks to regenerate, to put resources back into the system to improve its quality and resilience and support biodiversity (Timmis, Ramos, and Verstraete 2022; https://www.ellenmacarthurfoundation.org/the‐circular‐economy‐in‐detail‐deep‐dive).

The strategies underpinning the circular economy are varied and range from policy through economics to recycling by individual households. Product design is key to the circular economy because up to 80% of the environmental impacts of products are determined at the design phase (https://environment.ec.europa.eu/topics/circular‐economy_en). Because transitioning from linear to circular economies necessitates paradigm–process–economic–behavioural changes, education is crucial for both understanding of what is needed and achieving broad acceptability. Importantly, microbial technologies play vital roles in many different components of the Circular Economy, ranging from water purification for reuse (https://asm.org/articles/2020/april/how‐microbes‐help‐us‐reclaim‐our‐wastewater; Metcalf and Eddy Inc. An AECOM Company et al. 2007), metal recovery from digital devices (Chauhan et al. 2018; Han, Teo, and Yew 2022), resource recovery from wastewaters (Pikaar et al. 2022), energy generation from wastewaters (Tchobanoglous, Burton, and Stensel 1991; Bazina et al. 2023), the creation of new, better recyclable materials such as microbial polymers (Zhu, Romain, and Williams 2016; Pereyra‐Camacho and Pardo 2024) and cellulose (Wang, Lu, and Zhang 2016), and less polluting extraction processes through biomining (Ehrlich and Newman 2008; Jerez 2017).

## 10 The Potential of Microbial Activities to Counter Disease, Injury and Mortality Caused by Aggression: Warfare, Violence, Discrimination and Abuse

In humans, aggression is expressed in different forms—physical and psychological—at different levels, in different circumstances, including abuse of the vulnerable, discrimination, demonisation of groups and nations, conflicts and warfare. It is often expressed in the context of political polarisation, in and out grouping and ‘othering’ (https://ethicsunwrapped.utexas.edu/glossary/in‐group‐out‐group; Hitlin, Kwon, and Firat 2021), which are often exploited by individuals to gain and maintain personal power, influence and wealth. Comprehensive data on harm and suffering visited by humans on humans are lacking because of extremely low reporting. Data on warfare‐violence lethality indicate that the number of premature deaths per 100,000 range between 50 and 500 (https://ourworldindata.org/grapher/global‐death‐rate‐in‐violent‐political‐conflicts‐over‐the‐long‐run; https://ourworldindata.org/war‐and‐peace; https://ourworldindata.org/grapher/deaths‐in‐armed‐conflicts; https://www.dw.com/en/global‐conflicts‐death‐toll‐at‐highest‐in‐21st‐century/a‐66047287; Rutar 2024). However, these numbers are those easily measured and just the tip of the iceberg because, as is the case for current conflicts in 2024, a large number of people not killed directly during the conflict experience all manner of injuries and deprivation, including the stress‐anxiety of experiencing traumatic events (Alburez‐Gutierrez et al. 2024), displacement and forced migration, poor access to food, hygiene, healthcare and medicines, education, etc., and human suffering, including grieving for lost family–friends and fragmentation of social groupings, that can translate into massive human suffering, initiation of new and exacerbation of existing health conditions including stress‐anxiety‐neuropsychiatric disorders, and poorer quality of life, all of which can result in premature death. The long‐term effects on the young are not known but are significant. These, coupled with long‐term interruptions in education, have life‐changing consequences, analogous in some ways to those of ‘long Covid’. The global health impact of violence and aggression has not been quantified but it can be assumed that it is huge.

While warfare is all about killing and maiming personnel of the ‘other side’, prevention of loss of life and suffering, and healing of personnel of the ‘own side’ are also central elements of warfare. This latter includes vaccination of personnel against infections anticipated in theatres of war, and measures to reduce infections resulting from conditions of poor hygiene that are typically experienced, but also treating physical and mental injuries of affected military and civilian personnel (see also https://www.nato.int/cps/en/natohq/official_texts_224669.htm). Available microbial technologies relevant to treatment of physical injuries include antibiotics, microbially produced biocompatible wound dressings, and microbially derived promoters of wound healing (Rivero‐Buceta et al. 2020; Canchy et al. 2023; Yin et al. 2024).

Since it is known that anxiety‐stress are influenced by the gut microbiota, there may be opportunities for microbiota interventions that lessen the effects of, and accelerate recovery from, trauma‐induced mental problems. Posttraumatic stress disorder (PTSD) in both combatants and civilians, especially the young, is a common outcome of war, developing in 15% of people exposed to trauma (https://www.who.int/news‐room/fact‐sheets/detail/post‐traumatic‐stress‐disorder#:~:text=An%20estimated%203.9%25%20of%20the%20world%20population%20has%20experienced%20PTSD,conflict%20or%20war%20(3). However, almost 4% of the global population experience PTSD during their lifetimes, with rates particularly high following sexual violence. A recent study found that adolescents with PTSD had a distinct gut microbiome profile and lower microbial diversity compared to resilient individuals. Lower microbiome diversity was associated with more posttraumatic symptoms in early childhood, increased emotional and behavioural problems in adolescence, and poor maternal care‐giving. The study also revealed less mother–child microbial synchrony in youth with PTSD, suggesting that reduced microbial concordance between mother and child may indicate susceptibility to posttraumatic illness. Importantly, when germ‐free mice were transplanted with microbiomes from individuals with PTSD, they displayed increased anxiety behaviour, suggesting that the trauma‐associated microbiome profile contributes to the anxiety component of PTSD. This important study highlights the potential role of the microbiome as a biological marker for PTSD risk and resilience, and suggests new avenues for microbiome‐related diagnosis and treatment following trauma (Yirmiya et al. 2024).

Another relevant aspect of microbes in the context of warfare is the potential use of pathogens as weapons (Casadevall and Pirofski 2004; Oliveira et al. 2020; Gani Mir et al. 2022). While most countries have long abandoned microbial weapon research because of the lack of predictability and the logistics of handling and delivery, the relatively low cost of microbial weapons keep them as options for less technically advanced groups, especially terrorist groups. The anthrax attack of 2001 is one such example (Bush and Perez 2012). The COVID‐19 pandemic was a timely reminder that the issue of biological warfare needs to remain in focus (Gostin and Nuzzo 2021). Microbial warfare research and development in most countries focuses on developing barriers—defensive strategies—which include early detection systems (Gani Mir et al. 2022) and response strategies that include new vaccines (Croucher 2024), phages, antibiotics, as well as handling strategies for delivery media: air, water, food, fomites.

The production and use of munitions is associated with environmental pollution which is harmful to health. Mapping and remediation of contaminated sites is thus essential and microbial biosensors for pollutant detection and bioremediation processes involving microbes and microbe–plant partnerships that degrade the pollutants is a promising option (Lewis, Newcombe, and Crawford 2004; van Dillewijn et al. 2007; Kalsi et al. 2020).

Violence and aggression may be characteristic of wars but are also generally prevalent in society and impact health (e.g., see Wang, Fu, et al. 2022). A report by the Organisation for Economic Cooperation and Development (OECD; https://www.oecd‐ilibrary.org/docserver/health_glance‐2017‐8‐en.pdf?expires=1726900577&id=id&accname=guest&checksum=5451ACDC424800864A175A53EF79BF20) identifies diet, smoking and alcohol consumption as playing important roles in global deaths, with violence, accidents and self‐harm being important in ‘external’ causes of death. Alcohol consumption is sometimes linked to violence and abuse, which in turn are linked to physical and mental injuries. Alcohol consumption and substance abuse may also be linked to risk‐taking, like dangerous driving, unsafe sex, etc., associated with higher probabilities of injury and suffering. Thus, violence and aggression are influenced by a range of factors that are networked and partly reinforcing and downward spiralling. Since these are in part behavioural in nature, their roots often lie in mental make‐up/state, tendency to risk‐taking, aggressivity, upbringing and other influences, some of which have been associated with the human microbiome.

One fascinating study has shown that the microbiome is implicated in aggressive behaviour in fruit flies (Grinberg et al. 2022) and a recent study from the same group demonstrated the microbiome also played a role in aggressive behaviour in a mouse model (Uzan‐Yulzari et al. 2024). In both cases, germ‐free or antibiotic‐treated animals were more aggressive than their wildtype control. Surprisingly in both models, recolonization with bacteria (mono‐colonisation in flies or faecal microbiota transplantation (FMT) in mice) caused aggression levels to return to normal. Changes in aggression levels were accompanied with changes in pheromone and metabolite levels and also changes in gene expression levels. The researchers also conducted an FMT experiment in mice with faecal material from 1 month‐old human babies who had received antibiotics during the neonatal period, or not, and demonstrated again that antibiotic treatment in the infants increased aggression in the transplanted mice. Given that aggressive behaviour is responsible for a considerable amount of human suffering at all levels of society, from personal relationships, to road rage, to wars, the possibility of modulating it through targeted microbiota interventions should be considered. It is also worth noting that the gut microbiota is also implicated in substance abuse, so there may be opportunities for microbiota interventions to ameliorate its practice and effects (Kazemian and Pakpour 2024) (Table 1).

**TABLE 1:** Microbial technology pathways to raising barriers to preventable human suffering, morbidity and mortality.

Health challenge	Mitigating and potentially mitigating microbial technologies
Infections	*Surveillance*
	Molecular and serological diagnostic methods/biosensors for pathogen/(endo)toxin monitoring and quality control of clinical materials (such as blood products and solutions for intravenous infusions), healthcare facilities, food (from farm to fork), wild and domestic animal sources of zoonotic infections, drinking water, recreational waters, wastewaters, built environment, etc. POCTs
	*Transmission*
	Wastewater treatment Drinking water treatment High‐touch surface probiotics Antimicrobial biochemicals *Wolbachia* technology Food fermentation to prevent pathogen growth/reduce burdens
	*Prevention*
	Vaccines for protection, eradication and achievement of herd immunity Identification of conserved, essential antigens of pathogens with highly variable surfaces Maintenance of a healthy, diverse microbiome
	*Infection diagnosis*
	Molecular and serological diagnostic methods POCTs
	*Therapy*
	Antibiotics Therapeutic antibodies Alternative antimicrobial therapies Microbiota transplants/editing
Antimicrobial resistance	Rapid methods to determine resistance profiles Discovery and development of new antibiotics Co‐therapies, including non‐lethal agents targeting drug resistance mechanisms, strategic cell structures and functions Therapeutic use of pathogens of infecting agents: phages, etc. CRISPR‐Cas strategies to inactivate pathogens and/or their drug resistances Bacterial therapeutics engineered to inactivate pathogens, virulence products, antibiotic modifying enzymes Antimicrobial nanomaterials and ‘Nanobiotics’ Vaccines targeting AMR ESKAPE+ pathogens Microbiota transplants Monitoring of AMR and its spread Elimination of the practice of using antibiotics as growth promoters in food production, through the development and deployment of microbial alternatives to antibiotics
Pathogenic biofilms; persisters	New antibiotics that kill non‐growing/non‐metabolising cells
Pathogens in poorly‐accessible body sites	‘Weaponised’ bacterial membrane vesicles
Non‐transmissible diseases	Disease detection‐diagnosis and monitoring
	Deployment of biosensors and diagnostics that detect disease biomarkers
	*Cancer*
	Vaccines against viruses causing cancer Antibiotics against bacteria causing cancer ‘Weaponised’ cancer cell‐targeting bacteria, minicells, membrane vesicles Therapeutic antibodies Microbiota interventions
	*Metabolic disease*
	Promoting healthy gut microbiota and physiology through increasing dietary fibre, fermented food intake, precision nutrition, and reducing ultra‐processed food intake Microbiota interventions Nutraceuticals
	*Microbiota dysbiosis*
	Pre‐, pro‐, symbiotics Fibre and precision nutrition to create and maintain a healthy gut microbiome Microbiota transplants/editing
	*Neuropsychiatric disorders, including post‐traumatic stress disorder*
	Psychobiotics Gut microbiota interventions
	*Addictions: alcohol and substance abuse, ultra‐processed food, internet, etc., moods, stress, etc*.
	Psychobiotics Promoting healthy gut microbiota and physiology through increasing dietary fibre, fermented food intake, precision nutrition, and reducing ultra‐processed food intake
	*Disorders of ageing: ‘leaky gut’, inflammaging, etc*.
	Nutrition and microbiome interventions to increase populations of microbes associated with a healthy gut
Injury and rehabilitation	Microbially produced biocompatible materials for wound dressings Biocompatible materials as scaffolds for tissue regeneration
Malnutrition	Microbial foods Fermented foods and probiotics Microbial production of food supplements/micro‐nutrients Microbial technologies to increase crop yields, soil fertility, and acreage of productive agricultural land
Intoxication	*Contaminated food‐mediated intoxication*
	Toxin detection diagnostics
	*Harmful algal blooms*
	Minimisation of nutrients in water bodies through use of agrobiologicals Improved wastewater treatment
	*Environmental pollution*
	Pollutant detection biosensors Microbial technologies to remove pollutants at source Bioremediation of contaminated sites Cleaning of radioactive wastes Ecosystem bioregeneration
Global warming	Biofilters for carbon capture at emitting sources Atmospheric carbon capture technologies Microbe:plant partnerships for carbon capture and burial Increasing the extent and efficiency of methane oxidising biofilters Approaches to reduce nitrous oxide emissions Microbial food production to reduce deforestation justified by a need for additional agricultural land Microbial energy production, such as hydrogen and bioelectricity to reduce fossil fuel burning; digestion of organic wastes to methane Microbial technologies to reduce carbon footprints of industrial processes, such as construction
Resource wastage	Microbial resource recovery, recycling and upcycling technologies
Poverty, deprivation and inequalities	Frugal health technologies and products POCTs Early detection and surveillance of pathogens (biosensors, real‐time environmental monitoring using next‐generation sequencing technologies, etc.) Thermostable vaccines with reduced cold‐chain requirements (e.g. DNA vaccines (Berger et al. 2024) Novel therapeutic interventions to fight against infections (bacterial therapeutics, phage therapies, pre and probiotics) Improved food and water safety (water purification by engineered microbes, biofortification) Better management of starvation and malnutrition through increased use of engineered microbes for fermentations to produce inter alia sustainable and affordable protein‐rich food, vitamins, antibiotics Improving balance of research to make it more relevant to LMIC settings, e.g. microbiome research, which is currently HIC population‐centric
Wars, conflicts, terrorism (including bioterrorism) and related consequences (i.e., migration, malnutrition, disease)	Microbially‐produced biocompatible materials for wound dressings Biocompatible materials as scaffolds for tissue regeneration Biodefence and counter‐bioterrorism (vaccines, microorganism‐based decontamination processes, predictive modelling using microbiological data and AI; see Danchin 2024) Rapid diagnosis and POCT tools to detect specific pathogens Infection control in overcrowded settings (antimicrobial coatings, air purifiers, UV‐based sterilisation systems, etc.) Supporting mental and physical recovery (through microbiome research and regenerative medicine) Global collaboration (training programs and open data sharing)

## 11 Everyday Problems in Everyday Life: Stress, Neuropsychiatric Disorders and the Microbiome

While everyday life experience varies enormously among individuals and communities, in general most of it is occupied by work or education and sleep, with the rest usually filled with household chores, meals and leisure‐hobbies‐sport (lifestyle), including and especially electronic and social media activities. In many settings, each of these three daily activities roughly take up about 8 h or an equal third of the day. While everyday life can be stimulating, fulfilling and enjoyable for many, it can be a battleground for others and, for some, home or work‐school may represent confined spaces harbouring chronic stressors. This can create new or exacerbate existing neuropsychiatric disorders which can in turn worsen quality of life and work or education, and sleep experiences, which can then lead to a downward spiral. Some younger members of society who may be particularly concerned about global problems, like climate change, species extinctions‐biodiversity loss and environmental degradation, and influenced by social media may be especially susceptible to development of such disorders. The U.K. National Health Service motto of *Every mind matters* (https://www.nhs.uk/every‐mind‐matters/mental‐health‐issues/) is an inspirational principle that should guide mental health policy and strategy everywhere.

### 11.1 The Workplace/Classroom

Quality employment and job satisfaction are important for well‐being. While some employers and organisations create healthy work environments, other workplaces can be less than optimal, with problems in crucial aspects such as mismatching of expertise‐talent‐qualification and responsibility‐task, excessive workloads or work pace, support and supervision, fair and adequate remuneration, job insecurity, inequality and bullying‐harassment‐discrimination (e.g., https://www.who.int/news‐room/fact‐sheets/detail/mental‐health‐at‐work; Goh, Pfeffer, and Zenios 2015; https://www.who.int/health‐topics/social‐determinants‐of‐health#tab=tab_1). More than 50% of the global workforce works in the informal economy where there may be little or no job security and protection for health and safety (https://www.who.int/news‐room/fact‐sheets/detail/mental‐health‐at‐work). Such deficits can have major impacts on health, ranging from cardiometabolic issues to mental disease. According to the WHO, around 12 billion working days are lost each year to depression and anxiety, amounting to $ 1 trillion in lost productivity. Improving workplace conditions can significantly reduce such health impacts (https://www.weforum.org/agenda/2024/10/employee‐health‐programmes‐obesity/?utm_source=sfmc&utm_medium=email&utm_campaign=2838443_AgendaWeekly‐25October2024&utm_term=&emailType=Agenda%20Weekly). Paradoxically, though understandably, absence from the workplace, especially long‐term unemployment, creates its own stresses and neuropsychiatric disorders, *inter alia* because of loss of financial security, decrease in self‐esteem, social stigma and family–friend tensions (Roelfs et al. 2011; Roberts et al. 2023). Re‐employment can reverse these health impacts (Carlier et al. 2013).

Though different from adult employment, the school environment can create related stresses, resulting *inter alia* from periods of absence from school due to childhood illnesses resulting in disruption in knowledge acquisition pathways, and examinations, both of which can create insecurity and anxiety, harassment‐discrimination‐outgrouping, especially anonymous cyberbullying (https://www.thelancet.com/lrnals/lanchi/article/PIIS2352‐4642(17)30011‐1/fulltext; https://www.pewresearch.org/internet/2022/12/15/teens‐and‐cyberbullying‐2022/; https://www.healthdirect.gov.au/cyberbullying), competition and, in some cases, health‐impacting performance pressure from parents.

### 11.2 Sleep

Sleep disorders (https://www.ncbi.nlm.nih.gov/books/NBK19961/) are common, particularly in young adults (> 40%: McArdle et al. 2020), have a major impact on the quality of life (Institute of Medicine (US) Committee on Sleep Medicine and Research 2006; Pavlova and Latreille 2019), and are associated with chronic diseases, including cardiometabolic diseases, brain disorders, including cognitive function, behavioural problems and depression, tiredness (and, in adults, increased motor vehicle collisions), immune function and premature mortality (Holder and Narula 2022; Lim et al. 2023; Bermingham et al. 2023; https://www.cdc.gov/pcd/issues/2023/23_0197.htm). They are exacerbated particularly in children by the pervasive use of electronics and social media. A significant cause of sleep disorder is associated with experiences in the workplace and schoolplace: pressure, stresses and insecurity can all promote sleep disorders. Shift work is a major contributor (Kecklund and Axelsson 2016). Anxiety caused by cyberbullying can disturb sleep patterns especially in children.

### 11.3 Stress, Anxiety and the Gut Microbiome

The gut microbiota, via the gut‐brain axis (perhaps also via inflammatory responses of the immune system: Peirce and Alviña 2019), plays significant roles in mental well‐being and neuropsychiatric disorders, as well as sleep disorders (see above, and also Ritz et al. 2024; https://www.sciencedirect.com/book/9780323999717/the‐gut‐brain‐axis). These roles are associated with microbiota diversity and composition, which in turn can be influenced by nutrition, microbiome‐based therapies (prebiotics, probiotics) and lifestyle. Thus, a combination of workplace‐schoolplace environment optimisation, combined with a focus on optimal diets and lifestyle, should result in significant mitigation of both neuropsychiatric and physical disorders. Moreover, as key microbial players in stress and sleep patterns are revealed, targeted interventions to modulate gut microbiome composition and functionality, and personalised nutrition, should become possible.

The gut microbiome has been shown to play a significant role in sleep physiology and circadian rhythm biology, and gut microbiota diversity and dysbiosis are correlated with sleep disorders (Krueger and Opp 2016; Smith et al. 2019; Yue et al. 2023). Importantly, sleep habits and gut microbiota compositions of infants seem to influence behavioural and developmental outcomes by influencing brain maturation (Schoch et al. 2021). This led to the suggestion that both sleep patterns and gut microbiota may represent attractive intervention targets in infants since both can be re‐shaped non‐invasively. Moreover, since some adult diseases have their origins in infancy, such interventions may have long‐term protective effects in some individuals (Schoch et al. 2021). If this proves to be the case, it will be hugely important because it will open pathways to improve long‐term health by acting early in life, involving parenting, nutrition and microbiome modulation. While this could affect many people in ‘normal’ environments, it will be particularly important in environments that are inherently prejudicial to mental health in general, and sleep patterns in children in particular, such as conflict/war zones, with their heightened anxieties, mental and physical traumas, and accommodation‐food‐healthcare‐education insecurities, refugee camps and informal settlements. Beneficial microbiome interventions in such cases may in future become a game‐changing damage repair strategy.

### 11.4 Leisure‐Hobbies‐Sport

The third of the day that may not be dedicated to work or sleep is filled with other various activities that include meals and their preparation, other obligations and, for some, diverse leisure and lifestyle activities that may include sport and hobbies. While none of these is totally free of health risks, one is particularly concerning. One of the most dramatic changes in human behaviour over the last 20–30 years is the explosion in digital connectivity, electronic entertainment, information and disinformation distribution, and social media, focused to a considerable extent on smart phone usage (https://ourworldindata.org/internet‐history‐just‐begun; https://ourworldindata.org/rise‐of‐social‐media; https://online.maryville.edu/blog/what‐is‐digital‐media/). As of January 2024, there were 5.35 billion Internet users worldwide, 66% of the global population, of which 5 billion were social media users, with the greatest use in age group 15–24 (http://www.pvmarquez.com/socialmediausagementalhealth).

Undoubtedly, the Internet and digital technologies represent a quantum advance in progress and have brought and are bringing massive benefits to humanity, including and especially in healthcare. That said, there is a dark side to the issue. On one hand, there is deliberate misuse of the technology by criminals, corrupt individuals and groups, and malign individuals and groups intent on harming others. On the other, there is ‘innocent’ but often excessive use of the technology. Involvement with digital media is addictive for many people and some suffer from clinical addiction: Internet addiction disorder (IAD)/problematic Internet use (PIU) https://pmc.ncbi.nlm.nih.gov/articles/PMC3480687/; https://www.sciencedirect.com/science/article/abs/pii/S0272735822000137?via%3Dihub. IAD/PIU are often accompanied by psychiatric co‐morbidities, including anxiety, poor impulse control and substance use disorders that can impact lives by causing social problems. Crucially, algorithms underpinning some platforms incorporate addictive features (https://www.cambridge.org/core/journals/business‐ethics‐quarterly/article/ethics‐of‐the‐attention‐economy‐the‐problem‐of‐social‐mediaaddiction/1CC67609A12E9A912BB8A291FDFFE799; https://edri.org/our‐work/exploitative‐designs‐how‐tech‐platforms‐harm‐young‐people/).

Even in the absence of Internet addiction, there is often intensive involvement, amplified by constant ‘prompts’ by various platforms continuously seeking attention (https://www.humanetech.com/brain‐science), and the autoamplifying messaging nature of social media, particularly with regard to misinformation and disinformation: *information pollution* (https://www.oecd.org/en/topics/sub‐issues/disinformation‐and‐misinformation.html; https://www.undp.org/eurasia/dis/misinformation; https://www.bbc.com/news/articles/c99v90813j5o; https://www.iccl.ie/wp‐content/uploads/2023/12/Ending‐artificail‐amplification‐of‐hate‐and‐hysteria.pdf). This intense involvement, often leading to information overload, can result in a number of common mental health issues, especially among young people, that include anxiety, poor sleep, poor body image, low self‐esteem, attention‐deficit disorders, addictive behaviours and depression (https://www.thelancet.com/journals/eclinm/article/PIIS2589‐5370(18)30060‐9/fulltext). Long term effects may also include 'brain rot' (https://www.theguardian.com/media/2024/dec/02/brain‐rot‐oxford‐word‐of‐the.year‐2024): reduced cognition (Firth et al. 2019), shortening attention spans (Moshel et al. 2024), changes in brain structure (Solly et al. 2022), reprogramming of behaviour (https://www.humanetech.com/brain‐science), and early onset of dementia ('digital dementia': Manwell et al. 2022). Some Internet communities promote alcohol abuse, with the health risks associated with this (https://pmc.ncbi.nlm.nih.gov/articles/PMC9722554/). These health impacts can be exacerbated by online communities that focus on promoting pro‐eating disorder behaviours (https://pubmed.ncbi.nlm.nih.gov/33672305/) and self‐harm and suicidality (https://pmc.ncbi.nlm.nih.gov/articles/PMC6278213/).

Even more disturbing are internet activities intended to cause harm. These include online harassment‐cyberbullying (https://www.thelancet.com/journals/lanchi/article/PIIS2352‐4642(17)30011‐1/fulltext), online violence (https://cdn.who.int/media/docs/default‐source/documents/child‐maltreatment/online‐violence‐final.pdf?sfvrsn=d8d2053f_2&download=true; https://iris.who.int/bitstream/handle/10665/364707/9789240062085‐eng.pdf?sequence=1) and child sexual abuse. Encrypted platforms, creation of false identities and the dark web/net (https://www.tandfonline.com/doi/full/10.1080/00396338.2016.1142085#d1e106; https://www.sciencedirect.com/science/article/pii/S240584402309477X) allows child sex offenders to have increased access to children and enables simultaneous mass pursuit of victims by individuals and trafficking rings (https://www3.weforum.org/docs/WEF_Typology_of_Online_Harms_2023.pdf; https://www.weforum.org/agenda/2023/10/world‐mental‐health‐day‐how‐to‐safeguard‐childrens‐mental‐well‐being‐in‐the‐digital‐era/; https://www.unicef.org/innovation/stories/protecting‐childrens‐rights‐in‐digital‐environments).

While the primary focus of countermeasures to digital media‐promoted neuropsychiatric disorders must obviously be at the level of digital media use, algorithm design and policing of platforms and removal of harmful content, microbial technology interventions designed to promote gut microbiota activities that positively influence mental disorders may have the potential to play a significant role in mitigating mental health consequences of the use of digital media.

### 11.5 Addictions and Addictive Behaviour

Internet addiction is just one example of the pervasiveness of addictions in society. ‘Addictions are chronic relapsing health conditions associated with many negative consequences at individual and population levels. These include, but are not limited to, higher morbidity and mortality rates for the addicted person, health and financial damages for family or community members, and increased economic and social costs for society as a whole’. (Konkolÿ Thege, et al. 2016; Effertz and Mann 2013). Addictions encompass both substance use disorders and behavioural addictions (Karim and Chaudhri 2012). Although there is discussion about what constitutes addiction, excessive use of tobacco, alcohol and illicit drugs, and excessive eating, gambling, video gaming, Internet use, falling in love, sex, exercise, work and shopping, are often included. According to one report, ‘47% of the adult population of the USA suffers from maladaptive signs of an addictive disorder over a 12‐month period’. (Sussman, Lisha, and Griffiths 2011). Addictions, like many disorders, impact the quality of life of the addicted, their relationships and their productivity.

Importantly, addictions may be multiple in nature: co‐addictions, co‐morbidities (Konkoly Thege, Hodgins, and Wild 2016). One example is addictive consumption of ultra‐processed food and drinks which are the perfect accompaniment to Internet‐social media‐video gaming activities that in some instances have eliminated ‘mealtimes’ and require food ‘on‐the‐go’, that is, hyperpalatable, ready‐to‐eat food (Monteiro et al. 2021) that can be consumed with little diversion of attention while otherwise occupied. Meal preparation and consumption at mealtimes demands time and effort, and hence impose a certain temporal‐scheduling discipline on daily behaviour‐habits and to some extent regulate engagement in activities such as Internet use. Ready‐to‐eat food lowers this imposition, reduces the need for regular meal times, and facilitates excessive Internet use.

Ultra‐processed foods and drinks contain or consist of ‘food substances never or rarely used in kitchens (such as high‐fructose corn syrup, hydrogenated or interesterified oils, and hydrolysed proteins), or classes of additives designed to make the final product palatable or more appealing (such as flavours, flavour enhancers, colours, emulsifiers, emulsifying salts, sweeteners, thickeners, and anti‐foaming, bulking, carbonating, foaming, gelling and glazing agents’; Gearhardt, Corbin, and Brownell 2016; Monteiro et al. 2019). Some of these components, such as high levels of refined sugar, fat, salt and other flavouring components, are considered to be addictive and have biological and behavioural effects that parallel those observed in substance addition (Gearhardt and Schulte 2021). Moreover, some of the additives of ultra‐processed food have been shown to have negative health consequences in animal models (e.g., Chassaing et al. 2015), as have compounds such as acrylamide which may be created during their manufacture, and compounds such as bisphenol A that may migrate from packaging materials into the food.

Food addiction affects around 1 in 7 of adults and 1 in 8 children. While the level of addiction in adults is comparable to other addictions, like those of tobacco and alcohol, the level of food addiction in children is ‘unprecedented’ (Gearhardt et al. 2023). Excessive use of the Internet and ultra‐processed food are examples of co‐addictions/co‐morbidities. Food addiction is also frequently associated with mental disorders (Horsager et al. 2021).

The growth in consumption of ultra‐processed foods is associated with a higher risk of adverse health outcomes (Du et al. 2024; Lane et al. 2024; for summary see https://bmjgroup.com/consistent‐evidence‐links‐ultra‐processed‐food‐to‐over‐30‐damaging‐health‐outcomes/), including and in particular with increases in obesity, type 2 diabetes and related disorders which are currently classed as a pandemic (Monteiro et al. 2021), and common mental disorders. Ultra‐processed food is also associated with an increased risk of gastrointestinal diseases and notably an altered gut microbiome and increased intestinal permeability and inflammation (Srour et al. 2022; Song, Chai, et al. 2023; Song, Song, et al. 2023; Whelan et al. 2024). Ultra‐processed foods lower microbial barriers to disease. There exists the possibility that mental disorders associated with ultra‐processed food may result from gut dysbiosis via the gut‐brain axis (Song, Chai, et al. 2023; Song, Song, et al. 2023). While confronting complex health issues like addictions and co‐addictions requires consideration of multiple contributing factors and a range of prophylactic and therapeutic options, microbiome interventions will probably play a significant role in raising barriers to disease (see also Carbia et al. 2023; Worth 2024).

## 12 Microbial Technology Barriers to Health Issues Associated With Urban Environments

### 12.1 Microbiology‐Relevant Health Aspects of Urban Life

A major challenge to human health is that settlements have become progressively more dense and urban, partly due to population growth, but also due to their multiplicity of varied logistical‐activity advantages (Bloom, Canning, and Fink 2008). Urban living will inexorably increase as a result of diminishing living space resulting *inter alia* from global warming‐caused rising sea levels, increasing extreme weather events and wildfires, and reducing habitability of affected regions, coupled with growth in the world population. Megacities and urban agglomerations that house millions of inhabitants will increase in number from the 85 currently counted, and in size: it has been reported that Shanghai will be merged with surrounding cities to create a megacity of 130 million (https://www.worlddata.info/megacities.php#:~:text=What%20is%20a%20megacity%3F,confused%20with%20a%20metropolitan%20area).

Urban environments are engineered, not natural, characterised by reduced content of nature: natural landscapes and their diverse soils, waters, airs, trees, animals and reduced physical exposure to them. They are landscapes covered with concrete, with the loss of soil ecosystem services and massive carbon footprints resulting from this. The very concept of urban settlements—economy of scale, the gain in efficiency of provision of goods and services to the maximum number of people in the minimal possible space—embodies their major downsides (Bloom, Canning, and Fink 2008; https://www.nae.edu/7529/MegacitiesandtheDevelopingWorld). Growth in urban environments often involves growth of slums (Ooi and Phua 2007; https://unhabitat.org/sites/default/files/download‐manager‐files/The%20Challenge%20of%20Slums%20‐%20Global%20Report%20on%20Human%20Settlements%202003.pdf; https://digitallibrary.un.org/record/639679?ln=en&v=pdf), with all the health consequences they embody.

Urban environments create the human equivalent of factory farming—crop plant and food animals maintained at high density in artificial environments—with all the negative ecophysiological and health consequences of these (this is not of course to imply that lower density living does not have its own problems). As in factory farming, high‐density living not only results in rapid exhaustion of local natural resources and the need to import replacement resources from elsewhere (often causing resource depletion elsewhere). Ecology is ecology and increased densities of host populations favour increased transmission and multiplication of pathogens, resulting in increased densities of pathogens. Thus, considerable effort is invested in prophylactic measures to reduce pathogen burdens, such as cleaning high‐touch surfaces with antimicrobial disinfectants, the equivalent of chemical pesticides. And, as with agrochemicals, this can lead to resistance to/tolerance of the antimicrobial agent and, in some cases, co‐selection and/or collateral resistance to other antimicrobials, including antibiotics in clinical use.

Urban living may be also associated with higher stress and workplace competition, and elevated peer pressure to follow certain lifestyle habits (Oliveira, Vidal, and Ferraz 2019), that lead to decreases in time allowed for food production and consumption. This trend is facilitated‐promoted through large‐scale production of ready, or at least rapidly and easily prepared, meals—ultra‐processed, low fibre (Ramsteijn and Louis 2024) ‘factory food’—whose composition is often influenced more by considerations of cost, distribution and shelf‐life than nutritional value, quality and true (as opposed to marketing) diversity. The fact that it is rapidly prepared coupled with lifestyle pressures, also encourages rapid consumption and resulting diminution of the benefits of extended and usually more relaxing mealtimes with accompanying restorative family‐social interactions and bonding. This in turn will tend to amplify existing anxieties, stress and other mental issues (see also Xu et al. 2023).

Agricultural expansion to feed a growing global population—invasion of new ecosystems—is predicted to drive the spread of infective agents (Rohr et al. 2019; Waage et al. 2022). Moreover, large scale, centrally prepared and distributed food will tend to transmit similar food‐borne microbes to everyone (also pathogens, as a result of centralised contamination, and thus has the potential to initiate large‐scale food‐borne infections, both accidental and deliberate/terrorist) and to support and select gut microbiomes that are more similar/less diverse among people. Even fresh foods, like fruit, vegetables, milk products and meat, will tend to be sourced from a few major suppliers able to supply the large quantities required, and hence have a limited microbial diversity, rather than a large number of small outlets collectively supplying products with greater diversity. This and other constraints on ambient microbial diversity, such as ventilation systems in large buildings, circulating the same air to everyone, and urban pollution, which reduces microbial diversity and selects pollutant‐tolerant microbes, and the widespread application of antimicrobial cleaning products, tend to reduce ambient microbial diversity and hence microbiome diversity in the population. The progressive reduction in microbiome diversity is currently considered to be associated with a reduction in microbiome services provided and reduced health resilience (Blaser 2018; Finlay et al. 2021; https://asm.org/articles/2019/november/disappearance‐of‐the‐gut‐microbiota‐how‐we‐may‐be).

Moreover, as noted above, air pollution is the leading environmental disease risk factor globally. Although indoor air pollution, often associated with cooking and heating, can be a serious health risk in rural communities, outdoor air of urban environments in general, and megacities in particular, can be highly polluted in many cases and a major cause of ill health (Grimm et al. 2008; https://www.bbc.com/news/articles/c1wj43vqdlpo; https://www.bbc.com/news/articles/c0k8dxpr8x5o). Young children are particularly vulnerable to air pollution and it is estimated that around 2 billion live in areas burdened with air pollution levels that exceed WHO limits https://www.unicef.org/sites/default/files/2019‐02/Clear_the_Air_for_Children_Executive_summary_ENG.pdf.

Urban life is also characterised by increased time spent indoors. This often means breathing air that is loaded with the microbiomes, especially airway microbiota, of others in the same building, including their respiratory pathogens, and of volatile pollutants inherent to most built environments (e.g., see Bruinen de Bruin et al. 2008), instead of fresh air outside blowing from the fields/sea, and being illuminated by lamps and having vitamin D deficiencies, instead of by sunlight that both kills airborne viruses and stimulates vitamin D production in skin.

In conclusion, urban life is increasingly being associated with lower quality nutrition, exposure to pollutants, particularly air pollution, low microbiome diversity and its protective and barrier functions that contribute to resilience, high pathogen densities, higher anxiety and stress levels, and increases in disorders such as autoimmune diseases (Zuo et al. 2018). How can the many virtues of urban settlements be enjoyed without being counteracted by their evils?

## 13 Microbial Technology Barrier Enabler: Exploration of Microbial Diversity to Mine New/Improved Drugs, Products, Activities and Services

The development of a new or improved technology to combat disease will often result from the targeted or untargeted discovery of a new or better biological product, activity or process. The microbial world, with its 3.8 billion year evolutionary history during which it biochemically and ecophysiologically explored a vast range of environments/substrates/energy sources, its huge phylogenetic diversity (one estimate suggests that there are one trillion species of microbes: Locey and Lennon 2016) and range of habitats colonised that are too hostile for most visible organisms, possesses an exceptional spectrum of activities and creates a vast range of chemicals and materials that greatly exceeds those of larger organisms. Prospecting the microbial world for new chemicals traditionally required their cultivation. Since only a tiny fraction could thus far be cultivated, there is a huge reservoir of functions remaining to be discovered. However, with genomics and metagenomics revealing potential functions of interest without cultivation, and recombinant DNA techniques permitting expression of such functions in culturable microbes (the cell factories; see below), the exploration, discovery and mining of new microbial products and functions is proceeding at an accelerating pace (Rodríguez et al. 2024; Van Goethem et al. 2024; Wang, Xiang, et al. 2024; Wang, Li, et al. 2024).

Key to the discovery of useful new products is the existence or development of selection systems and screens that access and identify what is sought. A simple screen deployed in early antibiotic discovery involved the ability of products secreted by potential producer organisms to inhibit test microbes. Microbes can now be engineered in a variety of ways to respond to a vast range of external signals that that identify sought products. Screens that target specific metabolic reactions/processes are particularly useful, especially combined with genomics/metagenomics to identify relevant protein:protein and protein:ligand interactions. This approach has been enormously simplified by the availability of the artificial intelligence‐based AlphaFold protein structure prediction software (https://deepmind.google/technologies/alphafold/) and the ability to model the interacting interfaces (https://blog.google/technology/ai/google‐deepmind‐isomorphic‐alphafold‐3‐ai‐model/#life‐molecules). Predicting interacting surface structures enables the identification of potential sites of action for the design of agonists and antagonists—drug candidates—that promote or prevent such interactions (see Timmis 2018; Abramson et al. 2024).

## 14 Microbial Technology Barrier Enabler: The Microbial Cell Factory

There are many microbial technologies that save lives but one of the most powerful and pervasive is the cell factory technology (Timmis and Hallsworth 2024). This is because it is highly versatile, can be deployed for so many applications, and is in constant evolution through the development of new genetic tools and the application of metabolic design and synthetic biology tools and strategies. A few examples are listed here for illustration.

### 14.1 Reagents

Cell factories are used to make reagents used in diagnostic procedures, such as DNA polymerases for PCR and LAMP.

### 14.2 Antibodies

Monoclonal antibodies, nanobodies, etc., used for diagnostics, prophylaxis and therapy, are mostly made in cell factories.

### 14.3 Antigens

Both natural and recombinant antigens used for diagnostics and vaccines, such as the HBV surface antigen produced in yeast for the HBV vaccine, are made in cell factories. The immune response stimulatory potency of antigens may be enhanced by diverse microbial technologies, including glycoengineering (Lehri et al. 2024).

### 14.4 Microbially Inspired Drugs

Perhaps the most important sector of cell factory application is in drug production. Most new antibiotics are discovered in microbes which use them as weaponry in their own ecological battles (e.g., Rodríguez et al. 2024; Van Goethem et al. 2024; Wang, Xiang, et al. 2024; Wang, Li, et al. 2024). Once discovered, an antibiotic is tested for application potential: activity spectrum, selective toxicity‐safety, stability, pharmacokinetics, and so forth. Materials for testing are usually produced from the organism in which the activity was first detected—which thereby acts as a cell factory—either a natural isolate from the environment or a recombinant organism into which a suite of new genes have been transferred. As the most promising candidates advance through the pipeline, their therapeutic properties are improved by chemical modification, often removal/addition/substitution of groups. Sometimes this modification is achieved by chemical reactions but, given the complexity of many natural products that serve as starting points for clinical drugs, chemistry can often be difficult, and biochemistry is necessary. Cell factories are used extensively for the production of enzymes that modify the structure of drugs and drug precursors, some of which are made by synthetic chemistry, and endow upon them properties desired for clinical use. They are also used to produce precursors and building blocks of drugs (e.g., Hernández‐Fernández et al. 2024). Using microbes as chemists leads to green and/or eco‐friendly manufacturing reducing the pollution burden to the planet.

### 14.5 Enzymes

Although enzymes used in the production of drugs are vital for preventing and reducing suffering and loss of life, they only represent a small fraction of enzyme production in cell factories. For example, cold‐active enzymes—proteases and lipases that remove food and other stains from clothing—are used extensively in detergent powders and liquids employed in automatic washing machines, enabling them to be run at much lower temperatures than is the case for detergents lacking the enzymes (Kuddus et al. 2024; Oliva et al. 2024). As a result, there is a significant saving in energy used by washing machines, which translates into a lower carbon footprint, a lower contribution to global warming and climate change, and hence a reduction in suffering and lives lost due to climate change. Another important example of enzyme production in cell factories is phytase, used in animal feed, especially feed for monogastric animals. Phytate is the major form of phosphorus in plants but is largely unavailable to the animal. Phytate also binds other nutrients in the gut and hence is an antinutritional factor. Phytase hydrolyses phytate and thereby makes available not only phosphorous but also other nutrients and increases significantly the nutritional value of animal feed, which in turn reduces the amount of feed needed. The production of animal feed comes with high carbon, environmental and agricultural resource footprints, which translate into contributions to global warming‐climate change, environmental deterioration and competition for human plant food production and hence food security, all of which impact health. Microbial production of phytase is therefore another example of microbial technologies that contribute to human well‐being. Indeed, enzymes from cell factories are exploited in many, very diverse ways, some of which have positive direct or indirect impacts on health.

### 14.6 Metabolites

Microbial cell factories are also extensively used to produce simple metabolites, such as amino acids and vitamins essential to healthy nutrition, sunscreen compounds like melanin, microsporine and microsporine‐like amino acids, and scytonemin (Cordero, Vij, and Casadevall 2017; https://enviromicro‐journals.onlinelibrary.wiley.com/doi/10.1111/j.1751‐7915.2010.00241.x/), which reduce skin damage by ultraviolet light and hence skin cancer. Other compounds, including some that serve as basic scaffolds for organic synthesis of drugs and other compounds of value, can be also obtained by cell factories. A striking example is the production of the anticancer drug vinblastine in engineered yeast cells (Zhang, Hansen, et al. 2022), which includes 30 enzymatic steps encoded by 34 heterologous genes from plants. Another example relevant for health applications is the production of fluorinated precursors in engineered bacteria (Pardo et al. 2022), which is relevant when considering that one quarter of all pharmaceutical drugs in the market contain fluorine decorations to enhance bioavailability (Haas and Nikel 2023), among other advantages.

### 14.7 Advanced Materials

Microbial biotechnology is revolutionising the field of materials science, with bacteria emerging as powerful producers of biopolymers that hold significant potential for biomedical applications (Moradali and Rehm 2020; Blanco et al. 2021; Hernández‐Arriaga et al. 2022; Pereyra‐Camacho and Pardo 2024). Through advancements in microbial biotechnology, synthetic biology and metabolic engineering, bacterial biopolymers are being transformed into customizable, high‐performance materials for a range of applications, particularly in medicine and sustainable industries. Three primary bacterial biopolymers—bacterial cellulose, polyhydroxyalkanoates and γ‐polyglutamic acid exemplify distinct classes of polysaccharides, polyesters and polyamides, respectively, each offering unique properties that make them ideal for critical applications such as drug delivery, tissue engineering and regenerative medicine. These biopolymers present sustainable and biocompatible alternatives to traditional synthetic materials, addressing both medical and environmental needs.

Bacterial cellulose, produced by species like *Komagataeibacter*, stands out for its exceptional mechanical strength, biocompatibility and high water‐holding capacity. Its nanoscale fibrous structure closely mimics the extracellular matrix, making it particularly suitable for tissue scaffolds, wound healing and vascular grafts. Moreover, its ability to support host cell growth and tissue regeneration, alongside its efficient drug‐carrying capacity, positions bacterial cellulose as a valuable material for controlled drug delivery systems.

Polyhydroxyalkanoates are biodegradable polyesters synthesised by bacteria such as *Cupriavidus necator* and *Pseudomonas putida* . These biopolymers are widely used in drug delivery systems due to their ability to encapsulate drugs and ensure sustained release, thus enhancing therapeutic effects and reducing toxicity. Furthermore, polyhydroxyalkanoate‐based scaffolds show immense promise in tissue regeneration, with applications in bone and cardiac tissue engineering. Genetic engineering has further enhanced the production of polyhydroxyalkanoates, broadening their suitability for various medical uses, including sutures and regenerative medicine.

γ‐Polyglutamic acid, synthesised by bacteria such as *Bacillus subtilis* , is a water‐soluble, biodegradable polymer with excellent potential in drug delivery and tissue engineering. Its ability to form hydrogels makes it particularly useful for developing scaffolds in tissue regeneration and controlled drug delivery systems, especially in challenging environments like the gastrointestinal tract.

To maximise the utility of these biopolymers, advanced technologies are employed to optimise bacterial production. Metabolic engineering plays a key role in enhancing biopolymer production by fine‐tuning bacterial biosynthetic pathways, transforming bacteria into highly efficient biofactories capable of producing large quantities of tailored biopolymers for both medical and industrial applications. Synthetic biology enables precise control over bacterial production processes, facilitating the creation of engineered living materials that possess smart functionalities such as self‐repair and responsiveness to environmental stimuli. These innovations are paving the way for the development of materials that can dynamically respond to biological signals, thereby opening up new possibilities for smart drug delivery systems and tissue regeneration. Further customisation of bacterial biopolymers is achieved through chemical modification and diversification, using techniques like cross‐linking, grafting, and blending with other polymers. These methods enhance the flexibility, biocompatibility and antimicrobial activity of the materials, making them particularly effective in wound healing, tissue engineering and drug delivery applications. An emerging and promising approach involves the development of hybrid living materials, which combine living bacteria with synthetic components to produce materials with adaptive properties. These materials can self‐assemble, self‐heal and adapt to their environment, rendering them especially suitable for biomedical applications. Additionally, innovative techniques such as bioprinting and cell encapsulation have enabled the creation of complex, three‐dimensional materials that support cell growth and maintain biological activity, thereby further expanding the potential uses of bacterial biomaterials in therapeutic applications.

Microbial cell factories are also used to produce nanomaterials of various types for agricultural, environmental, nutritional and medical applications (Li et al. 2011; Yang et al. 2022; Carmona et al. 2023). In medicine, they are used *inter alia* in diagnostic procedures and biosensors, imaging, as biomaterials for tissue regeneration and in drug delivery systems. Some nanomaterials have antimicrobial, antifungal, insecticidal or anticancer activities (Arora, Lashani, and Turner 2024). Nanomicrobiology is also being used to develop new materials for electronics, chemical catalysis and separation science. Nanomaterials have been traditionally produced by physical methods which are inefficient, have high energy needs and require the use of toxic chemicals. Nanomicrobiology, which is based on the metabolic transformation of input materials to nanomaterials like metal, oxide, sulfite and other nanoparticles, is characterised by high efficiencies and low energy and toxic chemical inputs, so is more environmentally friendly.

### 14.8 The Factory Itself (or Parts of It) as the Barrier Agent

Cell factories are also used to design cells or cell components with novel features, such as surface decoration (surface display) with foreign antigens, nanobodies or ligands that provide important functionalities that can be exploited in various ways, especially for medical interventions, including the engineering of microbiomes (e.g., Timmis et al. 2019; Timmis, Soffritti, Mazzacane, et al. 2019; Shen et al. 2022; Srivastava and Lesser 2024).

Extracellular vesicles (EVs) are subcellular membrane‐bound structures released from cells that contain all types of cellular materials (nucleic acids, proteins, metabolites, sugars, fatty acids, etc.): their so‐called ‘payloads’ (György et al. 2015). They readily fuse with other cells, delivering their payloads, and are thought to be important means of intercellular communication. Importantly, they tend to have low toxicity and good permeability. EVs are produced by cells of both prokaryotes and eukaryotes and in principle those from one branch of the tree of life can fuse with cells of other branches. As a consequence, EVs are currently perceived as offering many possibilities for efficient delivery of specific payloads to diverse types of target cell for a range of applications. Cells producing EVs can be engineered so that EV surfaces are decorated with functional structures that serve as immunising antigens, lipopolysaccharides to trigger good immune responses (Gerritzen et al. 2017; Jiang et al. 2021), and ligands that bind to specific structures, for example cancer cell‐specific antigens, and hence constitute ‘homing devices’. Applications include diagnosis (e.g., of cancer: Chronopoulos and Kalluri 2020), active and passive prophylaxis with vaccines (Micoli, Adamo, and Nakakana 2024), therapy, especially of infectious diseases and cancer (Gao, Yujie, and Wang 2022; Long et al. 2022), and tissue repair and regeneration (Liu et al. 2022; Liang et al. 2022).

Bacterial minicells are anucleate cells budded off parental cells that have a defect in cell division (https://pubmed.ncbi.nlm.nih.gov/34516078/). Although analogous to EVs, they are quasi normal cells, with a similar structure and complement of cellular functions except that they lack a genome. As a consequence, they are more robust than EVs, are easy to isolate, are relatively homogeneous, and can also be engineered/treated to carry specific payloads. In addition, as indicated above, their surfaces can be decorated with ligands, for example ligands to tumour‐specific antigens, which endows them with the ability to home in on specific targets (Brahmbhatt and MacDiarmid 2021). Recent clinical trials with anti‐tumour minicells show promising results (Ganju et al. 2024). A further development of this is the surface display of cytokines on tumour‐homing bacteria which boosts the activity of local immune effector cells that destroy hard‐to‐treat tumours (Fidelle and Zitvogel 2024).

Living microbes are also an attractive platform for detecting and treating disease in vivo (see Vargason and Anselmo 2018; Srivastava and Lesser 2024). Microbial cell factories can produce a wide array of therapeutic agents, including single‐chain antibodies, enzymes or cytokines. Engineered bacterial therapeutics have shown efficacy in animal models for various pathologies, including infectious diseases, cancer (Kalia et al. 2022), inflammatory bowel disease and cystic fibrosis (Piñero‐Lambea, Ruano‐Gallego, and Fernández 2015; Riglar and Silver 2018; Mazzolini et al. 2023). The possibility for real‐time monitoring and in situ production of therapeutic agents offers tremendous possibilities for targeting diseases that are currently difficult to manage.

### 14.9 Cell Factories and Nutrition

As mentioned above, cell factories are used to create foods and fermented foods. Metabolically designed cell factories are also used to produce food supplements, like amino acids and vitamins, but also neutraceuticals like curcumin, which has anti‐inflammatory, anti‐oxidant and anticancer properties, in addition to being an important food colourant (Beganovic and Wittmann 2024), natural food colourants (Thomsen, Nielsen, and Borodina 2024) and flavours (Guo, Luo, et al. 2024; Guo, Ding, et al. 2024).

Probiotics have been mentioned multiple times in this discourse: classically they are cell factories that operate inside our bodies and belong to the class of agents that also include prebiotics, synbiotics, postbiotics and neutraceuticals. Probiotics are live microorganisms used to confer health benefits on an individual when administered in adequate amounts. Prebiotics are ingredients non‐digestible by hosts that selectively stimulate the growth and activity of probiotic bacteria in the digestive tract. Synbiotics are a combination of pro‐ and prebiotics. Postbiotics are dead microbes and/or their components that offer health benefits to the host. Neutraceuticals are biological products incorporated into diets that have health properties. All of these can act as regulators of gut microbiota and are viewed as having considerable potential for preventing and treating infectious and metabolic diseases such as obesity, type‐2 diabetes, and fatty liver disease (see also Alessandri et al. 2024), as well as neuropsychiatric disorders, all of which are associated with an imbalance of gut microbiota. Recently, it has been suggested that gut microbiota manipulation by pre‐ or probiotics may become a potential therapy for polycystic ovary syndrome (Babu et al. 2024). In order to protect the viability of probiotics during transit through the gut, carrier materials and prebiotics may be required. New technological improvements will help to develop functional foods and neutraceuticals that increase the viability and effectiveness of probiotics (Li et al. 2021). Interesting examples include a riboflavin‐producing probiotic (Dricot et al. 2024), *Lactobacillus helveticus* probiotic attenuation of alcoholic liver injury (Lv et al. 2024), and grape seed extract prebiotic that modulates the gut microbiota to prevent oestrogen deficiency‐induced bone density reduction (Lu et al. 2024). Of course breastfeeding provides infants with a multitude of probiotics and prebiotics, in addition to antibodies and nutrients (Masi and Stewart 2024).

On the other hand, the term probiotics has more recently been extended to include applications that modify microbiota outside of the body, be it on surfaces of humans (e.g., in wound healing: Nakatsuji et al. 2017; Canchy et al. 2023; Yin et al. 2024) or, for example, on the surfaces of inanimate objects, such as the high‐touch surfaces of hospitals and subways, in agriculture, and in the protection and preservation of cultural artifacts.

### 14.10 Cell Factories Saving Clean Water

Many industrial processes consume clean water, sometimes huge amounts as is the case in the semiconductor industry (https://www.weforum.org/agenda/2024/07/the‐water‐challenge‐for‐semiconductor‐manufacturing‐and‐big‐tech‐what‐needs‐to‐be‐done/). Given that fresh water and especially clean drinking water are limiting in parts of the world, which directly contributes to ill health through dehydration or use of contaminated water for drinking, or indirectly through lower crop yields or crop failures because of inadequate irrigation, processes to save fresh water are vitally important (https://turningthetide.watercommission.org). Seawater is not limiting in coastal regions and near salt lakes, so cell factory processes based on salt‐tolerant microbes, and using salt water for production, are of considerable relevance for sustainability. One example of such a salt‐tolerant microbe being developed as a cell factory is the fungus *Aureobasidium* (Xiao et al. 2024) which can produce a range of products, including melanin and polymalate (e.g., for delivery of anticancer drugs) (Table 2).

**TABLE 2:** Microbial cell factories for raising barriers against preventable human suffering, morbidity and mortality.

Barrier need	Examples of cell factory products that respond to need
Diagnosis: diagnostic reagents	DNA polymerases for nucleic acid amplification (PCR, LAMP) Antigens Monoclonal antibodies, nanobodies
Sensing	Whole cell biosensors for detection of disease biomarkers (also in food animals and plants), toxins, pollutants, emerging contaminants
Prophylaxis	Vaccine antigens, recombinant vaccines Prophylactic monoclonal antibodies
Therapies: microbially‐inspired pharmaceuticals	New pharmaceuticals/pharmaceutical scaffolds Weaponised microbes/microbial components (minicells, membrane vesicles) carrying surface display ligands for precise targeting and delivery of payloads Cas for CRISPR‐Cas applications Production of hormones (including growth hormone, insulin)
Nutrition	Microbial biomass as food/single cell protein Amino acids, vitamins for nutritional supplementation Probiotics
Catalysis: microbes as chemists	Enzymatically‐produced reagents, medicaments, micronutrients, etc. Low footprint enzyme catalysts for medicinal chemistry Enzymes that save energy (e.g. cold‐active enzymes for washing powders) Enzymes that upgrade food value (e.g. phytases) Enzymes that capture CO_2_ and other greenhouse gases Salt‐tolerant biocatalysts for bioreactors using seawater as the solvent/aquatic phase Active agents for bioremediation of environmental pollution
Metabolites	Pharmaceuticals, pharmaceutical precursors/scaffolds Anticancer agents Sunscreen compounds
Advanced materials	Bacterial cellulose for wound healing, tissue scaffolding and vascular grafts Polyhydroxyalkanoates and γ‐polyglutamic acid as biocompatible plastics and drug delivery systems, and tissue regeneration scaffolds in regenerative medicine Self‐assembling, environmental adapting polymers for biomedical applications Microbially‐produced nanomaterials, *inter alia* for biosensors and as anti‐microbial, insecticidal and anti‐cancer materials, and especially as imaging materials

## 15 Microbial Technology Barrier Enabler: Diagnostics and Sensors

### 15.1 The Prompt Diagnosis Barrier

In general, the earlier disease is accurately diagnosed, the earlier appropriate treatment can be initiated and the better the outcome. Diagnostic methods, reagents, instruments and trained personnel are thus pivotal to reducing human suffering and avoidable premature death. Microbial technology contributes massively to the diagnostic effort, either by providing microbial‐originating reagents, such as antigens and enzymes, or producing these and other reagents in microbial cell factories, or engineering microbes as biosensors to detect biomarkers of disease, in vitro or in vivo (Turjeman and Koren 2021; Capin et al. 2024).

### 15.2 Programmable Microbes for Diagnostics and Therapy

Living cells can process a myriad of signals in parallel and compute an adapted output via their signalling networks. Microbes are attractive candidates to engineer next‐generation diagnostic tests, as they are inexpensive, easy to manipulate and store, while capable of sophisticated sensing and signal processing. Researchers have built microbial biosensors equipped with engineered receptors that detect molecules of interest, such as environmental pollutants or biomarkers of disease (see, e.g., Capin et al. 2024; Zhong et al. 2024). Proofs of concept have been made towards application to the clinics, detecting biomarkers such as glucose, bile acids, and zinc in serum or in faeces (Courbet et al. 2015; McNerney et al. 2019; Voyvodic and Bonnet 2020; Chang et al. 2021). Genetic circuits can also improve the robustness and sensitivity of such ‘bactosensors’ (Courbet et al. 2015). Microbial cell extracts can also be used to engineer biosensors (Voyvodic and Bonnet 2020). While barriers to deployment remain, microbial biosensors have an immense potential for providing highly versatile platform for affordable, sophisticated and decentralised diagnostics devices.

### 15.3 The Surveillance Barrier

As the military well know, *intel* (intelligence; knowledge of the enemy: known strategies and expertise, behaviour, predictability, numbers, battlefield, weaponry, etc.) is vitally important and can mean the difference between success and failure. The battles of humans against disease are also greatly influenced by knowledge of what infectious agents (*the enemy*) are doing and how they are evolving, particularly in the context of the microbiome (and its *noncombatant microbes*). Pathogen surveillance by regional and national public health authorities, and transnational and international organisations like the WHO, is fundamental to understanding the ecology/epidemiology and predicting the behaviour of infectious diseases, in order to mount the best possible defences and erect appropriate barriers. Pathogen detection and diagnostic tools and methods lie at the heart of surveillance and epidemiology, and most are based on microbial technologies. Polymerase chain reaction (PCR) and loop‐mediated isothermal amplification (LAMP) detection systems are based on microbial DNA polymerases and primers designed from the pathogen genome, whereas antigen detection systems are usually based on pathogen antigens, and sometimes also monoclonal antibodies/nanobodies, made in microbial cell factories. Other methods include genomic technologies, particularly proteomics and metabolomics, for rapid identification and quantification of key biomarkers of disease, and assessment of pathogens, AMR microbes and microbiota dysbiosis. The development of new CRISPR‐Cas systems hold much promise, not only for precision but also for low cost and ability to form the basis of easy‐to‐use POCTs (Pandya, Jagani, and Singh 2024).

The efficacy of deployment of these technologies was demonstrated in the recent COVID‐19 pandemic. They included centrally organised PCR detection of SARS‐CoV‐2 virus nucleic acids, lateral flow antigen detection kits for personal use, and community surveillance through PCR analysis of wastewater in treatment plants. All of these were vital for surveillance and reducing person‐to‐person transmission, as was genome sequencing to monitor the evolution of pathogen variants. Pathogen surveillance of wastewater has now become routine to provide early warning and monitoring of community infections (e.g., Girón‐Guzmán, Sánchez, and Pérez‐Cataluña 2024). LAMP tests have been used in some airports and LAMP‐based lateral flow tests are being developed.

## 16 Overarching Issues That Provide Important Context

### 16.1 Leaving No‐One Behind

The rallying cry of the United Nations—*leaving no‐one behind* (https://unsceb.org/sites/default/files/imported_files/CEB%20equality%20framework‐A4‐web‐rev3.pdf)—is not only hugely inspirational and the encapsulation of the humanitarian spirit, but also captures the range, diversity and interconnectedness and interdependency of relevant issues. It encourages the view of health through the lens of social and environmental ills. For example, poverty is frequently associated *inter alia* with poor nutrition, lack of clean water, poor hygiene, poor access to healthcare, especially preventive medicine, mental stress, all of which promote physical and mental ill‐health. Microbial technologies are a major component of the broader and growing bioeconomy which has the potential to create employment in regions where it is most needed (Timmis et al. 2014; Timmis, de Lorenzo, et al. 2017; Timmis, de Vos, et al. 2017) and thereby reduce poverty and associated health ills and, in some instances, also help realise the *demographic dividends* (Timmis, de Lorenzo, et al. 2017; Timmis, de Vos et al. 2017, and see below). However, creating employment must go hand‐in‐hand with improved education and training in skills essential to employment.

Forced human migrations resulting *inter alia* from poverty, hunger and conflicts also creates situations of poly‐deprivations that favour ill‐health, often in the context of refugee camps and informal settlements that sometimes also resemble war zones. Although such underlying causes of ill health are not the subject to this discourse (but see Anand et al. 2023), access to public health and healthcare services for monitoring, diagnosis, prophylaxis and therapy are even more essential than in high‐resource settings (although inadequacies are pervasive in most healthcare systems: Caldwell et al. 2024).

### 16.2 Challenge 5: Poor Resource Settings—The Issue of Health Inequity

Despite the power of existing, and the development of new and more powerful, microbial and other technologies for disease monitoring, prevention and therapy, their exploitation is highly unequal among peoples, with those in resource‐poor settings suffering major deficits and disadvantages (Hojat 2022; https://www.weforum.org/agenda/2024/06/investing‐in‐african‐health‐tech‐can‐transform‐health‐systems/). Levelling up requires political and economic commitment which are often hindered by short‐term and constituency considerations and priorities. However, access to quality healthcare is one of the conditions of maximising the demographic dividends (Fried 2016), the substantive potential socio‐economic benefits of demographic changes in the world. One of the barriers to healthcare equity is biased perceptions of priorities based on needs of high‐income countries rather than needs of larger populations in low‐income countries (Kruk et al. 2018), biases that can be countered to some extent by education, especially of the young (Timmis et al. 2024). Moreover, research that leads to medical advances is often HIC population‐centric, so may not always be applicable to LMIC populations and settings, as is the case for some current microbiome studies (Gulliver et al. 2024). Another, currently perceived as almost insurmountable, barrier is the absolute cost of levelling up, both in terms of materials and experienced personnel, so ways and means of reducing costs and personnel will lower this barrier (and ultimately also the bias barrier). However, in this case, there is currently considerable progress in the development of more affordable‐frugal‐microbial technologies and products, that include diagnostics and sensors, POCTs, probiotics for high‐touch surfaces in for example healthcare facilities that transmit pathogens, and 'smart microbes' ‐ bacterial probiotics and therapeutics for example to be deployed against gastrointestinal infections like cholera and childhood rotavirus infections prevalent in LMICs, and that promote wound healing (see Srivastava and Lesser 2024, and citations therein). Prioritising development of quality and effective frugal technologies and products will undoubtedly accelerate the process of dismantling health inequalities in all countries.

### 16.3 The Key Issue of Disease Burden Granularity

While it is normal and proper to focus on the numerically most important global health burdens, it is equally important to be aware of, and address, health burdens whose significance may not be high globally but important locally, regionally or in certain contexts. A classic example before the deployment of ivermectin was river blindness caused by *Oncocerca volvulus*. Communities along rivers in which *O. volvulus*‐carrying black fly (*Simulium* spp.) bred had high, almost universal levels of onchocerciasis which created a situation in which adults were blind with only children having sight and needing to be care‐givers of the adults (https://www.who.int/news‐room/fact‐sheets/detail/onchocerciasis). River blindness was catastrophic for affected communities. Onchocerciasis is a neglected tropical disease—NTD—defined by the WHO as “almost absent from the global health agenda…. NTDs have very limited resources and are almost ignored by global health agencies…. NTDs are diseases of neglected populations that perpetuate a cycle of poor educational outcomes and limited professional opportunities;… in addition, are associated with stigma and social exclusion.” (https://www.who.int/news‐room/questions‐and‐answers/item/neglected‐tropical‐diseases). NTDs include Chagas disease, dengue and chikungunya, leishmaniasis, schistosomiasis, leprosy, and others, and can be regionally important disease burdens. Invasive fungal infections are prevalent in some regions. Snake bites are major health issues in tropical regions. The dominant burden of physical and mental health in some regions is extended conflicts and warfare.

The problem of NTDs is intertwined with and compounded by other inequalities, such as poverty, inadequate access to clean drinking water, food, healthcare, housing and education, and unsustainable practices and policies, and are a significant challenge to the UN ideal of “leaving no‐one behind” and the NHS slogan of “every mind counts” (George et al. 2023; Sun and Amon 2018). Granularity of disease burdens matters and NTDs and other causes of disease and suffering specific to certain regions and situations need to be given attention equal to that accorded the top global causes of morbidity and mortality. NTDs must become an increased focus of effort for development of microbial technologies.

### 16.4 Towards a Well‐being‐Centric Economy

One of the most dramatic changes over the last century has been the increase in the human lifespan coupled with a decreasing working life, which has resulted in a longer pensionable life. This, and the facts that many societies have also experienced a lowering of fertility and increased engagement of women in the workforce, has resulted in significant demographic change, and the societal and economic consequences of this: changing patterns of consumption and recreation, the numbers of children in school, a reducing workforce, changes in input versus disbursement of tax revenues, increasing burdens of healthcare, pensions, care‐giving and so forth. A great deal has been written about the economic consequences of demographic change and about *demographic dividends* (https://www.imf.org/external/pubs/ft/fandd/2006/09/basics.htm; Bloom, Canning, and Sevilla 2003; Bloom, Canning, and Fink 2010; https://www.ncbi.nlm.nih.gov/books/NBK148831/; Ogawa et al. 2021): the potential for economic growth as a consequence of

- Labour supply in societies with young populations and workforces
- Wealth accumulation in societies with ageing workforces that is invested to some extent in national economies
- The social capital represented by retirees (the silver dividend: wisdom and generativity)
- The female gender capital represented by the higher proportion of women not yet in the workforce.

In all cases, realisation of the demographic dividends is not automatic and requires substantive investment *inter alia* in health and education (https://www.unfpa.org/demographic‐dividend#readmore‐expand; https://www.un.org/development/desa/dpad/publication/frontier‐technology‐issues‐harnessing‐the‐economic‐dividends‐from‐demographic‐change/; https://4dividends.prb.org) and a conducive and responsive policy environment (Bloom, Canning, and Sevilla 2003).

In most discussions of demographic dividends, health and education are viewed through the lens of economic performance and measures of it—gross domestic product, GDP—as necessary means of achieving economic benefits, of increasing human capital (but see also analysis on healthy lifetime income by Zhang et al. 2023). However, although healthcare and education are often viewed in terms of populations, their purpose fundamentally concerns the well‐being, nurture of talents and fulfilment of aspirations of the individual. In societies where individuals matter, economic benefits should have their purpose in improving the well‐being of individuals and society, rather than health and education having their purpose in driving economic performance.

The development and application of microbial technologies can bring economic benefits not only to the commercial enterprises that exploit them, but also more widely through enabling a healthier, better educated and more productive population. Improvements in education and health, which are mutually re‐enforcing, can offer substantial return on investment for individuals and society as a whole (https://www.unfpa.org/demographic‐dividend#readmore‐expand; https://www.un.org/development/desa/dpad/publication/frontier‐technology‐issues‐harnessing‐the‐economic‐dividends‐from‐demographic‐change/; https://4dividends.prb.org). This editorial provides a multitude of examples of microbial technologies that contribute to good health and there is good evidence that improvements in health can lead to stronger economies. https://www.sciencedirect.com/science/article/pii/S0277953624006439?via%3Dihub.

An increasing healthy life expectancy (https://www.gov.uk/government/publications/understanding‐the‐drivers‐of‐healthy‐life‐expectancy/understanding‐the‐drivers‐of‐healthy‐life‐expectancy‐report) can also bring a ‘silver dividend’ (https://www.adb.org/sites/default/files/publication/864241/ewp‐678‐population‐aging‐silver‐dividend‐economic‐growth.pdf; Kotschy, Bloom, and Scott 2024) from the economic and social capital of older adults living longer lives (https://iris.who.int/bitstream/handle/10665/356910/Policy‐brief‐1997‐8073‐2022‐1‐eng.pdf?sequence=1; https://cdn.who.int/media/docs/default‐source/decade‐of‐healthy‐ageing/decade‐proposal‐final‐apr2020‐en.pdf) and the desire of many to continue contributing to society (Erickson 1963; McAdams and Guo 2015). However, while the *average* participation in voluntary and charitable activities in Europe of individuals aged 65+ is 14%, there exists substantial variation with a distinct concentration among Northern‐Western European countries (range: 33% in Denmark vis‐à‐vis 3% in Romania) (Lee 2022). This clearly indicates that some regions of Europe are utilising and benefitting substantially more from the silver dividend than others. While various individual benefits of voluntary and charitable activities have been reported (for the age bracket 50+), such as ‘greater well‐being, encompassing life satisfaction, sense of purpose and meaning in life […], happiness, optimism, self‐confidence and feeling in control’ (Haski‐Leventhal 2009; Kim et al. 2020; Owen, Berry, and Brown 2022; as cited by Weziak‐Bialowolska, Skiba, and Bialowolski 2024), the personal decision to participate in voluntary and charity activities may reflect an underlying humanitarian conviction/motivation, desire to pay society back for benefits realised over the working life, loneliness (https://www.hhs.gov/sites/default/files/surgeon‐general‐social‐connection‐advisory.pdf), or simply an intention to continue working after retirement age, (Park and Shin 2023).

The silver dividend is expressed in various ways, including remaining in the labour force, education and care‐giving within and beyond the family, volunteer charitable work and the creation of philanthropic charities. While this effect is significant, it could undoubtedly be increased substantially by appropriate supportive organisational policies and actions (https://ntaccounts.org/doc/repository/NTA14.Lao.pdf; https://india.unfpa.org/en/news/indias‐ageing‐population‐why‐it‐matters‐more‐ever), including efforts to improve health (Fried 2016) and education of the older members of society, which is part of lifelong learning (Rocha de Jesus Fernandes and Lanza Queiroz 2024; Timmis et al. 2024). Moreover, it is also possible that the microbiome plays a role in the desire to participate, since it is known that microbiome structure and function are associated with mood and loneliness (e.g., see Cryan et al. 2019; Donovan et al. 2020; Finlay et al. 2021; Lopizzo et al. 2021; Kim et al. 2022; Falkenstein et al. 2024). Therefore, potential microbiome modulation to treat loneliness and associated neuropsychological disorders may have a collateral benefit in some of increasing the desire to become more involved in social activities, including those related to the silver dividend.

Similarly, there are economic and societal benefits to gain from reducing gender inequalities and enabling greater participation of women in the workforce (https://www3.weforum.org/docs/WEF_GGGR_2022.pdf). According to the International Labour Organisation, 72% of men but only 47% of women are in the global labour force (https://webapps.ilo.org/infostories/en‐GB/Stories/Employment/barriers‐women#global‐gap). There is thus a huge potential to increase the workforce and its economic benefits, calculated by one source to be $ 1 trillion (https://www3.weforum.org/docs/WEF_Closing_the_Women's_Health_Gap_2024.pdf; but see also https://www.unwomen.org/sites/default/files/2023‐09/progress‐on‐the‐sustainable‐development‐goals‐the‐gender‐snapshot‐2023‐en.pdf). Unequal participation in the digital workforce—the *digital gender divide*—is particularly notable (https://www.weforum.org/agenda/2024/09/south‐asia‐digital‐gender‐divide/?utm_source=sfmc&utm_medium=email&utm_campaign=2836567_AgendaWeekly‐27September2024&utm_term=&emailType=Agenda%20Weekly; according to UNICEF, ‘The gender digital divide has cost developing countries 1 trillion USD over the past decade’: https://www.unicef.org/rosa/media/27721/file/Agenda).

There are many reasons for the lower participation of women in the workforce, which vary from country to country and culture to culture, including personal choice, cultural constraints including ‘gender roles’, child marriage (https://documents1.worldbank.org/curated/en/530891498511398503/pdf/116829‐WP‐P151842‐PUBLIC‐EICM‐Global‐Conference‐Edition‐June‐27.pdf), child‐bearing/−rearing, home‐making, care‐giving, inadequate education and training (https://genderdata.worldbank.org/en/data‐stories/a‐tale‐of‐old‐and‐new‐gender‐gaps; https://sciendo.com/article/10.2478/izajodm‐2021‐0001), insufficient employment opportunities, poor working conditions, poor health (women spend 25% more of their lives than men in debilitating health: https://www3.weforum.org/docs/WEF_Closing_the_Women's_Health_Gap_2024.pdf), unequal (or zero) remuneration (https://www.oecd.org/en/topics/policy‐issues/gender‐equality.html) and other forms of discrimination (https://iris.who.int/bitstream/handle/10665/311314/WHO‐HIS‐HWF‐Gender‐WP1‐2019.1‐eng.pdf?sequ; see also https://www.unwomen.org/sites/default/files/2023‐09/progress‐on‐the‐sustainable‐development‐goals‐the‐gender‐snapshot‐2023‐en.pdf). More specifically, women disproportionately undertake unpaid, but socially essential, roles like caring for children or older people. A major challenge therefore is to ensure that unpaid caring and domestic labour is more equally shared between men and women, and appropriately valued. Crucially, there needs to be massive investment in women and girls, particularly in education (https://www.unicef.org/rosa/media/27721/file/Agenda; https://www.un.org/development/desa/dpad/publication/frontier‐technology‐issues‐harnessing‐the‐economic‐dividends‐from‐demographic‐change/) and health (Onarheim, Iversen, and Bloom 2016; Remme et al. 2020). Microbial technologies, in particular those concerned with health, nutrition, hygiene and provision of clean drinking water, reducing pollution, frugal technologies and global warming (‘Girls are also disproportionately affected by natural disasters and economic shocks’; https://www.unicef.org/rosa/media/27721/file/Agenda) have an important role to play in reducing the burden of ill health in women.

Measuring economic benefits only in terms of GDP undervalues many of the roles currently taken by women and over‐values commercial activities that are damaging to health like producing and selling health‐harming products https://wellbeingeconomy.org/wp‐content/uploads/WeAll‐BRIEFINGS‐Measuring‐the‐Wellbeing‐economy‐v6.pdf. It is therefore vital that society shifts the current focus of measuring GDP as an end in itself to developing a ‘wellbeing economy’ which is in service of human and planetary well‐being, rather than the other way round (Friel et al. 2023; https://www.weallscotland.org/what‐is‐a‐wellbeing‐economy). To ensure that the economic gains from microbial technologies support health and well‐being means ensuring that benefits are shared fairly and that adverse externalities are avoided. The next section of this editorial considers the use of Health in All Policies as a way to maximise benefits and minimise risks from any development, and strategies to avoid cost externalities.

### 16.5 Health in All Policies

The many examples in this paper show the potential for microbial technologies to contribute to better health in many ways. Yet there are also risks to population health if they are misused, or if access to these benefits is inequitable. Health in all policies (HiAP) is an approach that aims to gain maximum health benefit, and avoid health risks, from policies and interventions in any sector. The WHO defines HiAP as ‘an approach to public policies across sectors that systematically takes into account the health and health systems implications of decisions, seeks synergies and avoids detrimental health impacts, in order to improve population health and health equity’. (https://www.who.int/publications/i/item/9789241506908). HiAP often involves using processes like Health Impact Assessment (HIA) to assess the likely impacts on health and the population groups likely to be affected by these, to inform changes that maximise benefits and mitigate any risks.

The HiAP approach is built on collaboration and partnership between public health and other sectors (Green et al. 2021; https://iris.who.int/bitstream/handle/10665/112636/9789241506908_eng.pdf). Equity and participation are key values underpinning HiAP and HIA practice. (Green et al. 2021; Kemm 2013; https://iris.who.int/bitstream/handle/10665/112636/9789241506908_eng.pdf; Winkler et al. 2021). This means that the interests of the most vulnerable and highest need populations should be prioritised, and their views taken into account. It involves considering a comprehensive range of ways in which any policy or intervention might affect health, rather than addressing individual health issues at a time. (Green et al. 2021) Sustainability is also a key value for HiAP and HIA (Green et al. 2021; Winkler et al. 2021; https://iris.who.int/bitstream/handle/10665/112636/9789241506908_eng.pdf). While SDG 3 is specifically about health, the other SDGs are all determinants impacting population health outcomes. Some HIAs explicitly use the SDGs as a framework to identify relevant impacts for assessment (Green, Gray, and Ashton 2020; Winkler et al. 2020). It has even been argued that HIA practitioners were implementing the SDG framework before the UN even created it (Gulis 2019).

HIAs are applied to policies and plans related to many of the issues described here, especially those affecting social and environmental determinants of health such as urban planning, agriculture and industry. An understanding of microbial technologies may help HIA practitioners to identify opportunities to mitigate some of the adverse impacts identified. Similarly, HiAP has been little used to date to understand and influence the development and implementation of microbial technologies, but it has great potential to help ensure their benefits are realised and shared more equally. A future paper in this special issue will explore this potential in more detail.

### 16.6 Cost Externalisation

‘Externalised costs are costs of production that someone else pays’. (https://realitysandwich.com/sacred_economics_chapter_10/). The concept of cost externalisation usually refers to financial costs, for example environmental pollution associated with a commercial operation that engenders a societal cost through taxpayer‐funded remediation, commercial loss of revenue from tourism or fishing through oil spills on beaches that affect coastal businesses, increased healthcare costs due to increased disease from intoxication, etc. In all cases, these financial costs are not borne by the polluting producer (and hence not passed on to the customer who buys the products, or result in reduced dividends to shareholders) because they are externalised—transferred to others (https://www.imf.org/en/Publications/fandd/issues/Series/Back‐to‐Basics/Externalities). It is, however, apparent from this example there can also be non‐financial currencies of externalisation, in this case, the preventable human suffering and increase in morbidity and mortality caused by the externalisation.

Policies that embody acceptance, however reluctant or justified by cost considerations, of preventable morbidity and mortality engender major collateral costs that include unnecessary suffering, loss of days worked and corresponding revenue, time lost in education and its long‐term consequences for the individual and the family, a need for caring and the time and economic burden of this, emotional and mental strain on the individual and family and any mental disease consequences. Cost externalisation is therefore not just about money, but very much about health and well‐being—physical and mental—the transfer of health burdens to individuals and families, and the personal and collective responsibilities of those wittingly or unwittingly mediating the externalisation.

Cost externalisation issues relating to health range over multiple spheres which include:

- Vaccine hesitancy, which prejudices attainment of herd immunity and disease erradication, and increases risk of infection, and thereby transforms a personal preference/ideology (often acquired via societal trends and influences) into health burden costs of disease risk to individuals who are either unable to tolerate vaccination or for which vaccination is ineffective (example measles: Hotez, Nuzhath, and Colwell 2020; Opel et al. 2023), and of the associated unnecessary increased burdens on caregivers (Dhaliwal et al. 2022) and health systems that have to cope with these diseases
- Antibiotic use for viral diseases, which unnecessarily promotes the development and spread of AMR, and thereby transforms a perception of an improved individual health outcome into a collective health burden risk of increased morbidity and mortality to persons succumbing to infections untreatable with antibiotics
- Food supply and security, though as indicated above are essential to health, also engender health problems themselves on several fronts through associated carbon footprints and contribution to global warming, use of and pollution by agrochemicals‐eutrophication‐promotion of oxygen minimum zones and associated loss of biodiversity and ecosystem services (also caused by deforestation), water and agricultural resource use footprints, the health consequences of which are largely or entirely externalised
- Unnecessary bureaucracy and unbalanced/disproportionate restrictions *inter alia* take up valuable time of frontline health professionals (https://www.gov.uk/government/calls‐for‐evidence/reducing‐bureaucracy‐in‐the‐health‐and‐social‐care‐system‐call‐for‐evidence/outcome/busting‐bureaucracy‐empowering‐frontline‐staff‐by‐reducing‐excess‐bureaucracy‐in‐the‐health‐and‐care‐system‐in‐england) which reduces their availability for timely diagnosis and treatment of patient issues, some of which are based on microbial technologies, and can lead to poorer health outcomes. This externalisation of bureaucratic tasks to healthcare professionals is directly paid for by the healthcare professionals, who cannot fulfil their full potential to heal and, as a consequence, by patients who suffer poorer health outcomes, and their families and friends.
- Unnecessarily slow approval of new medicaments, medical processes, etc., that save lives. That this is in need of intense scrutiny and improvement is illustrated by the fast tracking of COVID‐19 vaccines which saved millions of lives and prevented many more COVID‐19 infections and long covid sequelae that would have occurred if regulatory approval schedules had followed their normal pace. COVID‐19 vaccination had an estimated global economic value of $ 5 trillion (Sevilla et al. 2024). Extrapolation of this indicates that more urgent consideration of other new developments and products would also have saved many lives. While processes of approval and regulation are designed to provide an essential high level of safety for new and existing drugs, imposition of excessive bureaucracy can equally engender unnecessary and preventable danger for those in need of a new drug. The processes of regulation and authorisation can thus externalise and transform unnecessary bureaucracy into a health burden of preventable suffering to patients, their family and friends.
- Lack of regulation to ensure safety of foods and other products in some settings, again transferring the health burden of preventable suffering to patients, their family and friends.
- The ‘evergreening’ of patents and other restrictions on intellectual property that prevents the sharing and use of technologies at affordable costs, and that externalises to the benefit of profits the health costs resulting from not deploying the technologies in settings unable to pay
- Policies based on ideology/dogma/agendas/narratives rather than evidence, such as the prohibition of recombinant ‘golden rice’ which combats vitamin A deficiency, a major cause of preventable morbidity and mortality in children (https://www.theguardian.com/environment/article/2024/may/25/greenpeace‐blocks‐planting‐of‐lifesaving‐golden‐rice‐philippines), which transfer official responsibility for healthcare to health burden risks of individuals and their families.
- A fundamental responsibility of all governments is to provide effective and equitable healthcare for all citizens, including the provision of clean water, clean air, unpolluted living environment and affordable nutritious food. Failure to do this, for whatever reason, is externalising the costs of political power to those in need of healthcare.
- This core responsibility of governments extends to responsible research and innovation (RRI) policy and budgets that support health‐related topics in the broadest possible sense. Vanity and lobby (pork barrel) projects (though obviously not high‐quality basic research) supported at the cost of health‐related projects is externalisation of future healthcare burdens to future citizens.

Cost externalisation not only causes unnecessary suffering and burdens, but conceals the true costs of actions and processes. Concealment is facilitated by ‘silo thinking’—consideration of individual issues that in reality are part of a whole—and hindered by systems thinking. There is an urgent need to expose and comprehend the consequences of cost externalisation wherever it occurs and to take steps to prevent it and compensate for it. When considering options in healthcare, it is essential to put all the relevant elements on the scale pans, not just selected ones that seem immediately relevant. Health impact assessments (https://www.who.int/health‐topics/health‐impact‐assessment#tab=tab_1) are crucial to this.

### 16.7 The Key Issue of the Behaviour of *Homo sapiens* and Its Path to Potential Extinction

In general, humans are living longer and healthier, so healthcare might be considered a success story and SDG 3 might be considered to be an optimisation exercise. However, although humans are living longer, the proportion of their lives associated with good health is not increasing (https://www.mckinsey.com/mhi/our‐insights/adding‐years‐to‐life‐and‐life‐to‐years). Moreover, although women, on average, live longer than men, on average they ‘spend 25% more time in “poor health” than men’.

(https://www3.weforum.org/docs/WEF_Closing_the_Women's_Health_Gap_2024.pdf; https://www.unicef.org/rosa/media/27721/file/Agenda). Improving these statistics is a major challenge for healthcare and, for this, new approaches are needed, especially in human/pre‐patient behaviour (see also https://www.goinvo.com/vision/determinants‐of‐health/).

Species extinctions mostly result from insufficient reproduction rates; since the human race is highly successful at reproduction, extinction might be considered to be irrelevant to *Homo sapiens* . However, according to one estimate, 99% of all species that ever lived on planet Earth are now extinct (Stearns and Stearns 2000), so why would *Homo sapiens* be different? Extinctions often result from insufficient resources to maintain populations of a critical mass. According to one estimate, there was a 69% decline in population sizes of larger animals, humans excepted, between 1970 and 2018 (https://wwflpr.awsassets.panda.org/downloads/lpr_2022_full_report.pdf). The growing human population is rapidly exhausting available resources of the biosphere. So perhaps species extinctions do hold some lessons for *Homo sapiens* .

Of course, *H. sapiens* is itself responsible for a large part of species extinctions as a result of its behavioural and consumptional activities associated in part with population growth and in part with its ecological excesses (Timmis and Hallworth 2022). This was encapsulated in the famous quote attributed to Gandhi: ‘The Earth has enough for everyone's needs, but not enough for everyone's greed’. Indeed, the growth in consumerism might well be considered to be an infectious pandemic with lethal potential. Thus, *H. sapiens* needs to seriously consider the possibility that one of the extinctions it causes will be its own. That this is not unlikely is evidenced by the fact that human activities have already compromised vital planetary processes, encapsulated in planetary boundaries and climate tipping points (https://www.thelancet.com/journals/lanplh/article/PIIS2542‐5196(24)00042‐1/fulltext; https://sciencebasedtargetsnetwork.org/about/what‐are‐sbts/; https://www.pik‐potsdam.de/en/news/latest‐news/earth‐exceed‐safe‐limits‐first‐planetary‐health‐check‐issues‐red‐alert). It is not currently known when the tipping point will be reached or, indeed, if it has already passed. What is known is that climate change is having a major impact on health, directly for example, by rising health‐compromising temperatures and increasing the geographical range of pathogens and their vectors, and indirectly in many ways, such as increasing desertification and hence hunger and poverty in affected communities, human migrations, overcrowding and reduced access to healthcare.

As clearly articulated by Merz et al. (2023), sustainability requires a massive change in the human mindset and behaviour. This will be facilitated by an understanding of relevant issues, an understanding requiring education that is currently inadequate (Paoli 2024; Timmis et al. 2024). Achieving necessary behavioural changes will be a serious challenge, especially for political leaders who must drive them, although there are encouraging signs that the general public, that is, *the voters*, and especially the young, support essential elements of the changes needed (https://earth4all.life/global‐survey‐2024/; https://www.weforum.org/publications/global‐risks‐report‐2024/digest/). However, the public and business must play a much more active role in change, especially in regard to consumerism and profligacy—the unnecessary use of resources. Advertising plays a major role in this, as do social media and ‘influencers’, including some members of the super wealthy who engage in *wealth vanity*: very publicly doing highly unusual, highly expensive things such as buying super yachts, private jets, super cars, walking in space (https://www.bbc.com/future/article/20240906‐from‐space‐selfies‐to‐nearly‐drowning‐the‐11‐spacewalks‐that‐made‐history), only because they can afford it, not because it is useful or necessary, Influencers and the super‐rich are role models for huge swaths of the general public, particularly the young, who get caught up in the practice of consumerism, unnecessary consumption and waste generation. These ‘role models’ need to pivot away from profligacy and make a public virtue of sustainable behaviour, because if *H. sapiens* goes extinct, they will have been a contributing factor. Everyone must play a part in protecting the planet and its fragile biosphere; everyone must start taking new and transformative decisions (see also Hechtlinger et al. 2024).

### 16.8 Health Education

Public education to achieve health literacy (https://www.who.int/news‐room/fact‐sheets/detail/health‐literacy) is a vital measure to increase *health capital*: to improve understanding of the issues that affect health, promote healthier lifestyles, achieve greater appreciation of public health measures (https://applications.emro.who.int/dsaf/emrpub_2012_en_1362.pdf; https://unesdoc.unesco.org/ark:/48223/pf0000381728; https://www.cdc.gov/healthyyouth/health‐education/index.htm), and both encourage and enable greater engagement in the self‐help that is needed to reduce the mounting burden on health systems worldwide (Timmis and Timmis 2017). While the dissemination of health knowledge should target all population groups, the most important is school children which have the highest learning capacity and will have the greatest (intergenerational) long‐term impact. Moreover, because children have not yet specialised and are minimally biased, they can be taught systems and critical thinking which enables them to see the interconnectivities and interdependencies that are crucial to the identification of causes and the exploration of evidence‐based solutions to problems. A concept for education of the general public in societally relevant microbiology, with special focus on school children, and on treatment of the issues of sustainability and critical and systems thinking, was recently published that may be instructive for health education, also in helping to create a more differentiated view of microbes and dispel widespread germophobia (Timmis et al. 2024).

## Disclaimer

Established microbial technologies, such as diagnostics, prophylaxes, drug production, wastewater treatment, agrobiologicals, processes to produce foods, food derivatives and supplements, etc., are powerful, applicable in many settings to solve diverse types of problem, and sustainable‐environmentally friendly. They prevent many millions of premature mortalities and unnecessary morbidities and suffering. Newer applications show great promise. However, evaluating applications in microbiome science, because of the extreme variation in human microbiomes, and the difficulty of establishing evidence for efficacy, are challenging (see also Zmora et al. 2018; Suez et al. 2018). Compounding the complexity of microbiota design and intervention is the complexity of causes of and risk factors influencing many problematic disorders, particularly those in which the environment plays a role (e.g., chronic intoxication by environmental pollutants), and especially neuropsychiatric conditions. Nevertheless, microbiota analysis in diagnostics, and microbiota intervention prophylaxis and therapies undoubtedly have significant potential. Some approaches are likely to be extremely successful, whereas others will be unsuccessful or only partially successful, applicable to a smaller proportion of medical conditions. Unfortunately, because there is considerable commercial interest in some developing technologies, some research progress is occasionally accompanied by handwaving and hype, which raises unwarranted expectations in the public that may not be fulfilled and that may ultimately lead to unhelpful scepticism. Cornerstones of scientific research are rigour and caution in interpretation and prediction: hype has no place and must be discouraged. What we have attempted to do in this discourse is to display the range of current microbial technologies that are applicable to current and future global challenges, and to give a flavour of the even greater diversity of applications in the pipeline. This does not mean that all pipeline possibilities will ultimately realise their current apparent potential.

## Concluding Remarks

The purpose of healthcare is to improve health and prevent avoidable disease, suffering and premature death. Considerations and efforts to improve human health tend to focus on individual aspects of healthcare as articulated in SDG 3 (‘reduce the global maternal mortality; end preventable deaths of newborns; end the epidemics; non‐communicable diseases through prevention and treatment; substance abuse; road traffic accidents; sexual and reproductive health‐care services’, and so on). Only at the end of the SDG 3 text is there a hint of the wider picture: ‘*3.d Strengthen the capacity of all countries, in particular developing countries, for early warning, risk reduction and management of national and global health risks’*. However, in order to confront effectively and comprehensively the most serious health issues on the horizon, it is imperative to pose the questions:

- What are the key nodes in multiparametric causes of preventable human suffering, disease and mortality?
- How can they be better managed?
- What are the key problems in present day delivery of healthcare?
- What are the greatest challenges healthcare will face in the future?
- What are the essential impediments/bottlenecks to confronting these challenges?
- How can barriers to societal inequities in healthcare and to efforts to reduce human suffering be lowered?
- What are the pathways to sustainbility in healthcare?

Global warming and associated climate change and extreme weather events are certainly a major, if not the greatest challenge, having pervasive negative impacts on health, ranging from direct effects of heat exposure, to loss of agricultural land resulting in reduced food production, ecosystem perturbations and losses, loss of living space causing human displacements to more crowded settlements, and so forth. When humanity goes extinct, and going extinct will be very much a health issue, global warming will be the most likely cause. Efforts to confront the multitude of health challenges of global warming obviously have to focus on reducing GHG emissions, increasing carbon capture and mitigating continued damage from the present climate threats.

The other major challenge is the increasing human population, which is also intertwined with global warming and is associated with a number of health issues, including those due to malnutrition resulting from insufficient availability of food. This means that food productivity needs to increase but, as we have indicated above, agrochemical‐based increases in food production creates further health problems and is unsustainable. Humans will increasingly live more densely in urban settings, especially megacities, and will suffer from overcrowding, increasing susceptibility to infection and other diseases, increasing exposure to pollution, reducing microbiome diversity, etc., all of which will reduce their resilience to stress and disease (https://www.weforum.org/publications/global‐risks‐report‐2024/digest/).

There are further important issues that need to share centre stage with the challenges of global warming and the increasing human population:

- Global and regional health inequities, with all their ramifications
- Neuropsychiatric diseases are increasing in prevalence (Ferrari et al. 2022; Wu et al. 2023), particularly among the young (Kieling et al. 2024), but a global plan to address this is elusive.
- Although the human lifespan is increasing, the human healthspan is not because the onset of age‐associated diseases has not markedly changed and increases in longevity have mostly been achieved by extension of the lives of those already suffering from disease (Crimmins 2015), ‘the pandemic of chronic diseases afflicting a growing older population’ (Garmany, Yamada, and Terzic 2021).
- While women live longer than men, they have a higher burden of morbidity‐driven conditions (Nusselder et al. 2019; Patwardhan et al. 2024).
- Despite the fact that an increasing number of individuals are becoming ever more fitness conscious, and increasing their engagement in physical exercise and ‘healthy food’, there is a global increase in anxiety and related mental issues and a decreasing microbiome diversity, all of which impact the ecology of human barriers to disease in different ways, including fertility. The challenge in future therefore will be to create health‐improving pathways that leverage this consciousness and determination to lead healthier lives, to attain a new level that integrates microbial technologies that maintain or increase microbiome diversity and create optimal microbiome functionalities associated with good metabolic, immune and mental health.

The aim of this discourse is not to be comprehensive and explore all possible microbial technologies that can help raise barriers to factors that promote human suffering. Rather it is to emphasise the exceptional range of challenges facing humanity and the applications and intervention opportunities available that can make a material difference in disease prevention, and to map them on the spectrum of processes that negatively impact human health, of the varied aspects of sustainability, ecosystem and planetary health, societal inequality, and of human activities and endeavours. It is imperative that current, and especially future, efforts are based on a much broader view of what negatively impacts health and how it can be countered. There is an urgent need to integrate comprehensively the diverse contributions to morbidity and mortality and the pivotal role of microbial technologies in healthcare strategies.

Microbial technologies are available to address many of the issues articulated in this discourse and indeed others. *However, and crucially, it is essential to recognise that advances in medicine* per se *are important but will not significantly address the more fundamental challenges facing humanity* (see also https://www.goinvo.com/vision/determinants‐of‐health/). Of course, although microbial technologies are key enablers of improved health for all, they can only achieve their potential in a conducive political, legislative and societal framework that *inter alia* seeks to protect the environment and biodiversity, and to conserve planetary health and resources (e.g., see Timmis and Ramos 2021; Gupta et al. 2024). *To be perfectly explicit: microbial technologies save lives and reduce human suffering; increasing their deployment will save more lives; not increasing their deployment—either though ignorance or design—is condemning humans to unnecessary suffering with the personal responsibility that such decisions embody*.

## Conflicts of Interest

G.C. received honoraria from Janssen, Probi, Boehringer Ingelheim and Apsen as an invited speaker, and research funding from Pharmavite, Reckitt, Tate and Lyle, Nestle, Fonterra and is or has been a paid consultant for Heel Pharmaceuticals, Bayer Healthcare, Yakult and Zentiva. The other authors declare no conflicts of interest.

## Data Availability

Data sharing not applicable to this article as no datasets were generated or analysed during the current study.
